# CENTRA: knowledge-based gene contextuality graphs reveal functional master regulators by centrality and fractality

**DOI:** 10.1093/nargab/lqaf196

**Published:** 2025-12-19

**Authors:** Frank Hause, Alice Wedler, René Keil, Laura Schian, Markus Glaß, Wiebke F Grimm, Oleksandr Sorokin, Wolfgang Hoehenwarter, Andrea Sinz, Stefan Hüttelmaier

**Affiliations:** Institute of Molecular Medicine, Section for Molecular Cell Biology, Faculty of Medicine, Martin Luther University Halle–Wittenberg, 06120 Halle, Germany; Center for Structural Mass Spectrometry, Martin Luther University Halle–Wittenberg, 06120 Halle, Germany; Department of Pharmaceutical Chemistry and Bioanalytics, Martin Luther University Halle–Wittenberg, 06120 Halle, Germany; Institute of Molecular Medicine, Section for Molecular Cell Biology, Faculty of Medicine, Martin Luther University Halle–Wittenberg, 06120 Halle, Germany; Institute of Molecular Medicine, Section for Molecular Cell Biology, Faculty of Medicine, Martin Luther University Halle–Wittenberg, 06120 Halle, Germany; Institute of Molecular Medicine, Section for Molecular Cell Biology, Faculty of Medicine, Martin Luther University Halle–Wittenberg, 06120 Halle, Germany; Institute of Molecular Medicine, Section for Molecular Cell Biology, Faculty of Medicine, Martin Luther University Halle–Wittenberg, 06120 Halle, Germany; Center for Structural Mass Spectrometry, Martin Luther University Halle–Wittenberg, 06120 Halle, Germany; Department of Pharmaceutical Chemistry and Bioanalytics, Martin Luther University Halle–Wittenberg, 06120 Halle, Germany; Center for Structural Mass Spectrometry, Martin Luther University Halle–Wittenberg, 06120 Halle, Germany; Department of Pharmaceutical Chemistry and Bioanalytics, Martin Luther University Halle–Wittenberg, 06120 Halle, Germany; Center for Structural Mass Spectrometry, Martin Luther University Halle–Wittenberg, 06120 Halle, Germany; Department of Pharmaceutical Chemistry and Bioanalytics, Martin Luther University Halle–Wittenberg, 06120 Halle, Germany; Center for Structural Mass Spectrometry, Martin Luther University Halle–Wittenberg, 06120 Halle, Germany; Department of Pharmaceutical Chemistry and Bioanalytics, Martin Luther University Halle–Wittenberg, 06120 Halle, Germany; Institute of Molecular Medicine, Section for Molecular Cell Biology, Faculty of Medicine, Martin Luther University Halle–Wittenberg, 06120 Halle, Germany

## Abstract

Deciphering gene function via context-aware approaches is limited by various means. Especially, static gene sets used in enrichment analyses and the lack of single-gene resolution restrain flexible association of genes with specific contexts. Here, we introduce CENTRA (Centrality-Based Exploration of Network Topologies from Regulatory Assemblies), a framework that models gene contextuality through topic-specific gene co-occurrence networks derived from curated gene sets and associated literature. Using latent dirichlet allocation on 12 045 abstracts linked to Molecular Signatures Database C2 gene sets, we uncover 27 biological topics and construct corresponding topic-specific networks reflecting distinct biological states, perturbation conditions, and disease-related regulatory programs. Graph-topological metrics, including centrality, local fractality, and perturbation sensitivity, were computed for each gene to capture structural relevance within these topic-specific networks. We show that topological profiles distinguish well-characterized regulators, identify emerging functional candidates, and reveal context-specific roles. Our framework prioritizes understudied genes by assessing the robustness of their topological signatures across topic-specific networks. To support exploration of these results, we developed a publicly accessible interactive browser, CENTRA, enabling dynamic navigation of networks and functional annotations. CENTRA provides an interpretable, scalable framework for investigating context-dependent gene function and hypothesis generation, offering a novel entry point beyond traditional enrichment approaches.

## Graphical Abstract

**Graphical Abstract:**
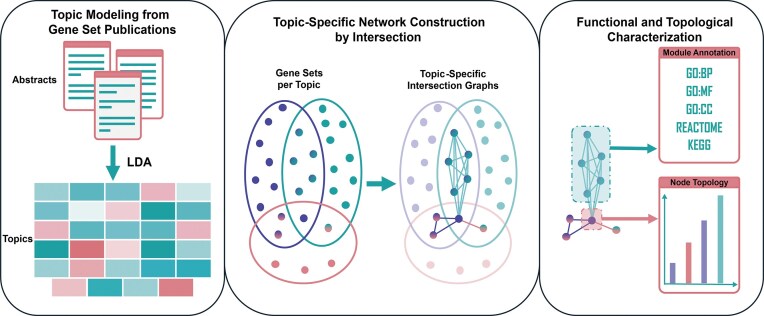

## Introduction

In most biological systems, genes and their products usually do not exhibit only one function exclusively. Gene function is rather highly context-dependent, shaped by cell type, developmental stage, physiological state, and disease condition [1, 2]. A canonical example of such context dependency is *TP53*, which under physiological conditions serves as a tumor suppressor by orchestrating senescence, DNA repair, and apoptosis [3, 4]. In contrast, when mutated in a tumorigenic context, the same gene can acquire gain-of-function properties that actively promote tumor progression, invasion, and resistance to therapy [5–7]. This functional duality underscores the necessity of evaluating gene activity within the specific biological and pathological context in which it occurs.

The context-dependence of gene function presents a critical challenge for widely used functional enrichment analyses [8], particularly overrepresentation analysis [9] (ORA) and gene set enrichment analysis [10] (GSEA). While both methods have become standard tools for the downstream interpretation of differential gene or protein abundance data, they share two fundamental limitations: (i) They require a predefined set of genes to infer functional or pathway involvement, and (ii) they do not explicitly account for the biological context in which this set, or any individual gene, is relevant. ORA assigns functional significance by testing whether predefined gene sets, reportedly associated with specific pathways or conditions, are statistically overrepresented among a list of genes of interest, under the assumption that these genes act in concert within a shared biological process. GSEA, by contrast, assesses whether genes from predefined sets are disproportionately enriched at the top or bottom of a ranked list, typically ordered by fold-change, thus assigning functional relevance based on gene abundance shifts in a yet context-dependent experimental setting.

Both ORA and GSEA rely on reference gene sets that have been experimentally linked to specific perturbations, pathways, or disease states. These gene sets are curated in public repositories such as the Molecular Signatures Database (MSigDB) [11, 12], particularly its C2 collection, which contains curated gene sets from canonical pathways (CP) as well as chemical and genetic perturbations (CGP). While these resources are invaluable for capturing broad biological themes, they are not designed to offer contextual relevance of individual genes. While researchers have the possibility to consult gene set annotations from different repositories when performing ORA or GSEA, the resulting enrichment terms are sometimes broad or generic, making biological interpretation difficult. This challenge is compounded by genes appearing in multiple gene sets linked to different pathways, blurring their specific functional context. More importantly, both approaches are fundamentally designed to assign function to sets of genes that show coordinated alteration under specific conditions, rather than to individual genes. As such, they cannot directly inform on the specific role or relevance of a single gene within a particular biological context. Gaining such gene-level insight typically requires integrating prior knowledge from literature; a process that is time-consuming, fragmented, and difficult to formalize within standard enrichment frameworks.

Topic modeling provides a computational strategy to systematically analyze large bodies of text and extract thematic patterns without relying on predefined categories or prior assumptions [13, 14]. It can be applied to entire publications or specific sections, such as abstracts or methods, to uncover latent semantic structures that reflect underlying biological concepts. A widely used approach in this domain is latent dirichlet allocation (LDA) [15, 16], a generative probabilistic model that represents each document as a mixture of topics, and each topic as a probability distribution over words. By inferring these topic distributions, LDA identifies hidden thematic groupings that link related concepts across large and heterogeneous corpora.

In this study, we leverage LDA to annotate curated MSigDB gene sets with latent biomedical themes, using these topic assignments to construct topic-specific gene co-occurrence networks, where co-occurrence refers to the joint presence of genes within curated gene sets of a given topic. Within each topic-specific network, genes are connected according to these co-membership patterns, enabling the computation of structural properties such as centrality, local fractality, and robustness measures. This topological embedding reflects a gene’s relative contextual importance, revealing both well-established regulators and understudied genes whose structural roles suggest latent functional relevance. The resulting framework not only addresses a critical gap in the functional annotation of genes under specific perturbations or disease states but also offers an accessible platform, CENTRA (Centrality-Based Exploration of Network Topologies from Regulatory Assemblies), for hypothesis generation and exploratory analysis. We anticipate that this approach will support a deeper understanding of context-specific gene function and guide future studies in systems biology, network medicine, and translational research.

## Materials and methods

### Construction of topic-specific gene co-occurrence networks

Gene sets and the abstracts of the reporting publications [for PubMed IDs (PMIDs) of included abstracts, see Supplementary File S1] were retrieved from the Curated Gene Set Collection C2 (CP and CGP) of the MSigDB (v2023.1.Hs) using the msigdbr package (Dolgalev, 2025). To compensate for missing PMIDs, gene set descriptions from Reactome [17], the Kyoto Encyclopedia of Genes and Genomes [18] (KEGG), and WikiPathways [19] were retrieved using their respective application programming interfaces (APIs) or web services.

Abstracts corresponding to the annotated PMIDs were retrieved from PubMed using the NCBI E-utilities (rentrez package) (Winter, 2017). In case no abstract was available, the original gene set description was used. If multiple abstract annotations were present for a single gene set, all corresponding abstracts were concatenated into a single document prior to downstream processing. Abstracts and descriptions (hereafter referred to as documents) underwent a preprocessing pipeline using tm [20] including (i) character normalization, (ii) removal of Greek and other non-Latin alphabet residues, (iii) lowercasing, (iv) punctuation and number filtering, (v) whitespace trimming, and (vi) stemming. Custom stopword removal was applied using a manually curated stopword list (see Supplementary File S2). Rare token (frequency <5) were iteratively removed.

From all preprocessed documents, a document-term matrix was constructed. LDA [15, 16] was used to infer latent topics across the corpus. The number of topics was estimated following Deveaud *et al.* [21], who proposed the Jensen-Shannon Information Divergence criterion for topic number selection. This metric suggested an optimal range between 15 and 30 topics (see Supplementary Fig. S1). Within this range, several models were generated and evaluated based on the top 100 β-weighted terms per topic, which were assessed manually for interpretability and internal consistency. Multiple rounds of model refinement were performed, during which the topic partitioning proved stable despite the stochastic nature of LDA. The final model was set to *k* = 27 topics. Topics were then assigned human-readable labels based on the top 100 β-weighted terms from each topic. A confidence threshold for the document-to-topic assignment was initially coupled to the number of topics, which for *k* = 27 would have retained roughly two thirds of all documents. Furthermore, constant thresholds of 0.1, 0.2, and 0.3 were tested, and a final value of 0.2 was chosen as the best balance between document retention and assignment specificity.

For each topic, pairwise co-occurrence of gene symbols was defined as their joint membership in at least one pair of gene sets assigned to that topic. Pairwise intersections were computed across all distinct gene set pairs within a topic; gene pairs identified in shared sets were represented as undirected and unweighted edges, with nodes corresponding to genes and edges reflecting set-based co-membership, using igraph [22]. Multiple overlaying edges were collapsed to unique connections to eventually form a knowledge-based topic-specific gene co-occurrence graph.

### Network-level analysis

Each topic-specific network was characterized by the following global topological metrics: node and edge counts, average degree, clustering coefficient, graph density, diameter, average path length, Louvain modularity, average betweenness, and closeness centrality.

To compare the structural similarity of networks not sharing the same node structure, spectral similarity was assessed using Laplacian eigenvalues [23] (top 20 components) of each network. Pairwise Euclidean distances between truncated spectra were computed to generate the corresponding distance matrix.

To assess node-level betweenness robustness (see below), local edge perturbation was simulated across 1 000 iterations (1‰ random edge rewiring per iteration) per network. This perturbation rate was chosen such that, on average, ~25% of nodes are affected in any given iteration (ranging from ~5% in the smallest networks to ~40% in the largest), ensuring sufficient opportunities for each node to gain and lose edges across iterations. For each iteration, betweenness centrality was recalculated. Per-gene variances in this metric were recorded, capturing the topological stability under minimal structural change possibly hinting at important roles of genes in a topic-specific network due to limited availability of those genes in the assigned gene sets. This procedure is sufficient to assess the robustness of the knowledge-based graphs constructed here but does not intend to make any propositions regarding the perturbation of gene regulatory or other molecular networks.

### Module-level analysis

Louvain clustering was used to define modules within each topic network. For each module, the following topological metrics were computed: node and edge counts, average degree, graph density, and average path length.

Module-level functional enrichment was performed using the ORA implementation of g:Profiler [24], restricted to annotations from Gene Ontology [25, 26] (GO), Reactome [17], and KEGG [18]. Enrichment results were computed with Benjamini-Hochberg-adjusted [27] *P*-values (false discovery rate <0.05).

To visualize functional relationships among topics based on their most significant biological processes, a semantic similarity map was constructed using the top enriched GO:BP terms per topic. For each topic, the 100 most significant biological process terms (based on adjusted *P*-values) were selected. These terms were mapped into a two-dimensional space using classical multidimensional scaling (MDS) of pairwise semantic similarity scores derived from GOSemSim [28] analysis.

The resulting MDS coordinates were used to calculate a centroid position for each topic, defined as the average semantic coordinates of its top 100 enriched GO terms. These centroids provide a summary representation of the functional position of each topic within the semantic “space” of the annotated network.

### Node-level analysis

Node-level analyses included topological metrics calculated for unweighted, undirected networks, specifically betweenness and eigenvector centrality. In addition, local fractal dimension (LFD) was estimated on a per-node basis following Xiao, Chen, and Bogdan [29]. The robustness of betweenness centrality was further evaluated through an edge perturbation simulation using local edge perturbations (see above). To facilitate comparability of metrics across different networks independent of their size or other structural properties, all node-level values were rescaled to the interval (0, 1) per topic-specific network.

### CENTRA implementation

To provide an accessible and interactive interface for exploring the resulting topic-specific networks, modules, and gene-level metrics, CENTRA was developed, a Shiny-based web browser application. CENTRA enables users to intuitively navigate and interpret the structural and functional properties of topic-specific co-occurrence networks derived from the curated gene set analysis. Designed to be low-barrier and hypothesis-generating, CENTRA allows researchers without advanced computational expertise to explore the topological metrics of different genes across diverse biological themes.

The application integrates the precomputed LDA topic assignments, gene co-occurrence networks, module clustering, functional enrichment results, and topological metrics at the network, module, and gene level. Users can interactively select topics, explore functional module enrichment, and comparatively inspect gene-specific metric profiles across the topic-specific networks. All visualizations are powered by networkD3 to ensure smooth interaction and responsive layout behavior.

CENTRA has been independently tested by several scientists with diverse backgrounds to evaluate its usability and interpretability. The application is freely accessible without registration at: https://ngs-info.medizin.uni-halle.de/shiny/CENTRA/.

## Results

### General analysis strategy

**Figure 1.**
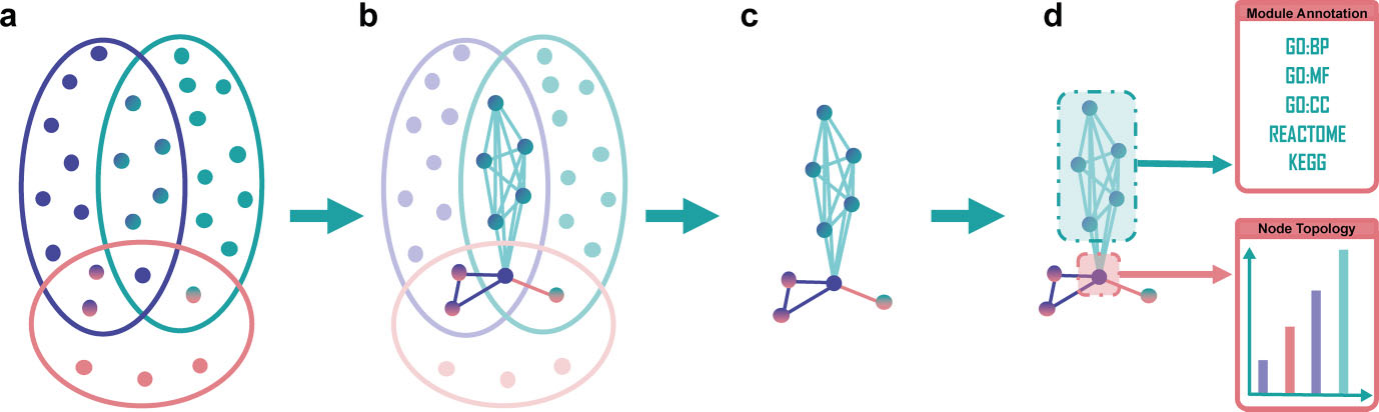
Construction of topic-specific gene co-occurrence networks. (**A**) Gene sets (colored ellipses) assigned to a topic via LDA exhibit partial overlaps in their constituent genes (colored dots). (**B**) Pairwise intersections were computed across all gene sets within a topic. Genes shared between sets were connected by undirected edges in an unweighted graph. (**C**) Recurrent edges from multiple overlapping gene sets were collapsed into single, unique interactions to generate a knowledge-based topic-specific gene co-occurrence network. (**D**) The resulting graph was further analyzed by identifying modules, performing functional annotation along with quantification of topological features at the node level.

To establish a method that, unlike ORA and GSEA, supports functional annotation with single-gene granularity and contextual relevance, we leveraged the gene set-reporting literature associated with the MSigDB C2 cluster of curated gene sets. Specifically, we applied LDA to cluster the abstracts linked to these gene sets into distinct biomedical topics, each representing a characteristic biological process, condition, or functional theme. This clustering enabled us to reorganize gene sets and their constituent genes based on co-occurrence within shared topical contexts. By assigning each gene occurrence to one or more topics through its inclusion in gene sets with overlapping textual associations, we constructed knowledge-based, topic-specific gene co-occurrence networks, in which nodes represent genes and edges denote co-occurrence within sets assigned to the same topic (see Fig. 1A–C).

The topological position of a gene within a topic-specific network serves as a proxy for its functional relevance within a given biological context [30]. To capture this, we computed a set of interpretable graph metrics that reflect different aspects of a gene’s potential role within a particular context: A high betweenness centrality highlights genes that act as bridges between otherwise unconnected regions of the network, pointing to potential integrators, regulators, or “bottlenecks” across functional modules. Eigenvector centrality extends the idea of gene’s importance characterized by a short overall distance to other genes by not only counting connections but weighing them by the importance of neighboring genes, capturing influence within the broader network hierarchy. Finally, high values of LFD reflect an increasing structural complexity in the vicinity of a gene, highlighting genes embedded in tightly organized or hierarchically structured neighborhoods [31].

Together, these metrics offer a multifaceted characterization of a gene’s contextual role, whether it is broadly connected, strategically positioned, or deeply embedded in a functionally cohesive cluster (see Fig. 1D). These features can signal different forms of biological importance, from hub-like activity to local specialization.

To account for genes that appear in only a few gene sets but may nonetheless play important roles within a given topic-specific network, the structural stability of their topological metrics was assessed through topic-specific random edge rewiring. In this procedure, networks were perturbed by randomly reassigning a small fraction of edges, followed by recalculation of betweenness centrality. This approach enabled the estimation of each gene’s topological robustness across perturbations, serving as a proxy for stability, and thus potential reliability, of its inferred contextual relevance.

Importantly, the variance of key metrics such as betweenness centrality across perturbed networks may offer predictive cues for interpretation. For example, low absolute centrality values combined with high variance suggest that a gene’s network position is highly sensitive to small changes in connectivity, such as a few edges being added or removed. This indicates that the gene can occasionally assume a central or regulatory role depending on minor alterations in the network structure, potentially reflecting a functionally relevant but understudied gene in the given topic. Conversely, high metric values with high variance may point to an unstable topological position, where apparent centrality arises from configurations that are easily disrupted. Such cases call for cautious interpretation, as network prominence may not be reliably sustained. In contrast, genes with consistently high metric values and low variance are more likely to represent robust and biologically meaningful regulators within their respective contexts.

To make these data and insights widely accessible, we developed CENTRA, a publicly available, interactive web application that allows users to explore these networks and gene metrics in an intuitive manner. CENTRA supports topic selection, enrichment exploration, and node-level metric visualization offering a novel entry point for researchers interested in the context-dependent roles of genes in health and disease.

### Probabilistic clustering of gene set documents reveals topic-specific network structures

**Figure 2.**
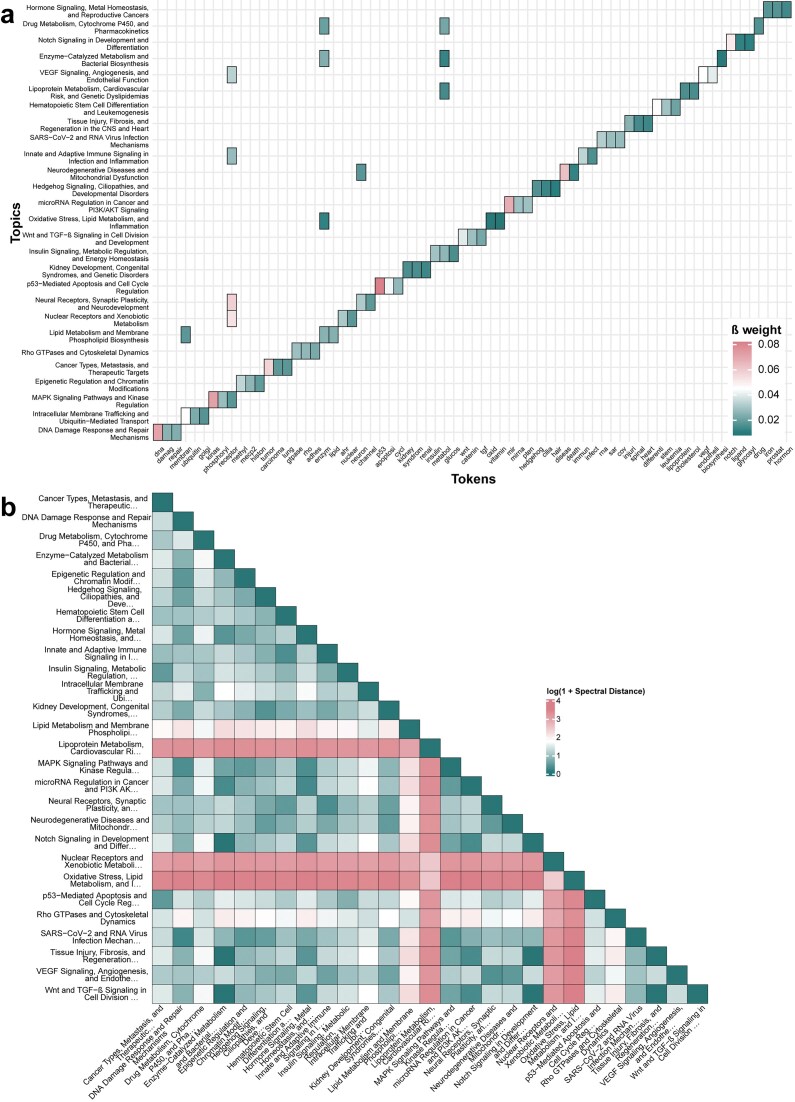
Topic modeling and network-level stratification of MSigDB C2 gene sets. (**A**) Token-topic distribution for the 27 topics derived from LDA modeling of 12 045 abstracts and gene set descriptions from the MSigDB C2 collection. Shown are the three highest β-weighted tokens per topic across all topics, with cell color representing the respective β-weight within the LDA model. For a more detailed overview, Supplementary File S3 provides the β-weights for the top 1 000 tokens per topic. (**B**) Spectral distance matrix indicating the structural similarity of the topic-specific gene co-occurrence networks. Values reflect log-transformed spectral distances, highlighting both closely related and structurally divergent topic-specific networks.

To uncover latent thematic structure within the corpus of gene set-associated literature, probabilistic LDA topic modeling was applied to 12 045 abstracts associated with 6 366 gene sets from the C2 collection of MSigDB. This nondeterministic approach yielded 27 distinct topics reflecting diverse biological processes, perturbation conditions, and disease-related regulatory programs. These topics are characterized by distinct token distributions that capture their semantic composition (see Fig. 2A). As a surrogate for this semantic composition, the β-weight reflects the relative importance of a token within a given topic. For example, in the topic “DNA Damage Response and Repair Mechanisms” the tokens “repair,” “damage,” and “DNA” yield the highest β-weights; “Lipid Metabolism and Membrane Phospholipid Biosynthesis” is enriched for “lipid,” “phospholipid,” and “metabolism;” while “Neurodegenerative Diseases and Mitochondrial Dysfunction” prominently feature “mitochondria,” “degeneration,” and “neuron.”

Documents were assigned to topics according to their posterior probability distribution in the LDA model. A threshold of 0.2 for the maximum posterior probability was chosen, balancing interpretability of topic content with the number of documents confidently assigned. Using this criterion, 2 052 documents were assigned to a respective topic. Topic assignment was unevenly distributed, with the largest number of documents assigned to “Cancer Types, Metastasis, and Therapeutic Targets” (*n* = 276), and the fewest to “Oxidative Stress, Lipid Metabolism, and Inflammation” (*n* = 12). It can be assumed that these stark differences mirror broader trends in biomedical research, where cancer continues to represent a dominant focus of scientific activity. A principal component analysis of the document-topic assignment confidence matrix further supports the discriminability of topic structures, with documents forming partially separable clusters in 2D space according to their topic assignments (see Supplementary Fig. S2). This degree of separation is consistent with expectations for LDA, which models topics as distinct yet potentially overlapping distributions. Indeed, while topics are clearly distinguishable, they are not fully orthogonal, a property that reflects the shared thematic content of many biological abstracts. This is further illustrated at the token level, where token such as “receptor” rank among the top three β-weights in five different topics, underscoring the biological interconnectedness of the thematic structures inferred.

Within each topic, gene co-occurrence networks were constructed by identifying shared genes across all pairwise combinations of gene sets assigned to that topic. These topic-specific networks varied considerably in size and topology, with key properties including node count (range: 48–12 922), edge count (range: 248–2 531 635), edge density (range: 0.017–0.537), and average degree (range: 10.3–391.8) (see Supplementary Table S1). Structural divergence between networks was quantified using pairwise spectral distances (see Fig. 2B). Topics with overlapping biological scope, such as “Lipid Metabolism and Membrane Phospholipid Biosynthesis” and “Lipoprotein Metabolism, Cardiovascular Risk, and Genetic Dyslipidemias,” exhibited low spectral distances, indicating structurally similar architectures. In contrast, networks derived from topically and mechanistically divergent contexts, e.g. “Oxidative Stress, Lipid Metabolism, and Inflammation” versus “Wnt and TGF-β Signaling in Cell Division and Development,” showed pronounced spectral separation. These findings demonstrate that meaningful gene co-occurrence networks can be derived by clustering the gene set documents using LDA, resulting in unique networks that inherently reflect the varying degrees of gene co-occurrence, regulatory modularity, and pathway specificity and thereby preserving the structural relationships of the embedded gene sets.

### Module detection reveals functionally unique assemblies within topic-specific networks

To assess whether topic-specific gene co-occurrence networks exhibit internally coherent substructures, Louvain community detection was applied to each network. This algorithm partitions the network into modules, densely interconnected groups of genes, based solely on topological properties. As the method is unsupervised and independent of prior biological knowledge, the resulting modules reflect purely emergent structural patterns derived from gene co-occurrence within each topic.

**Figure 3.**
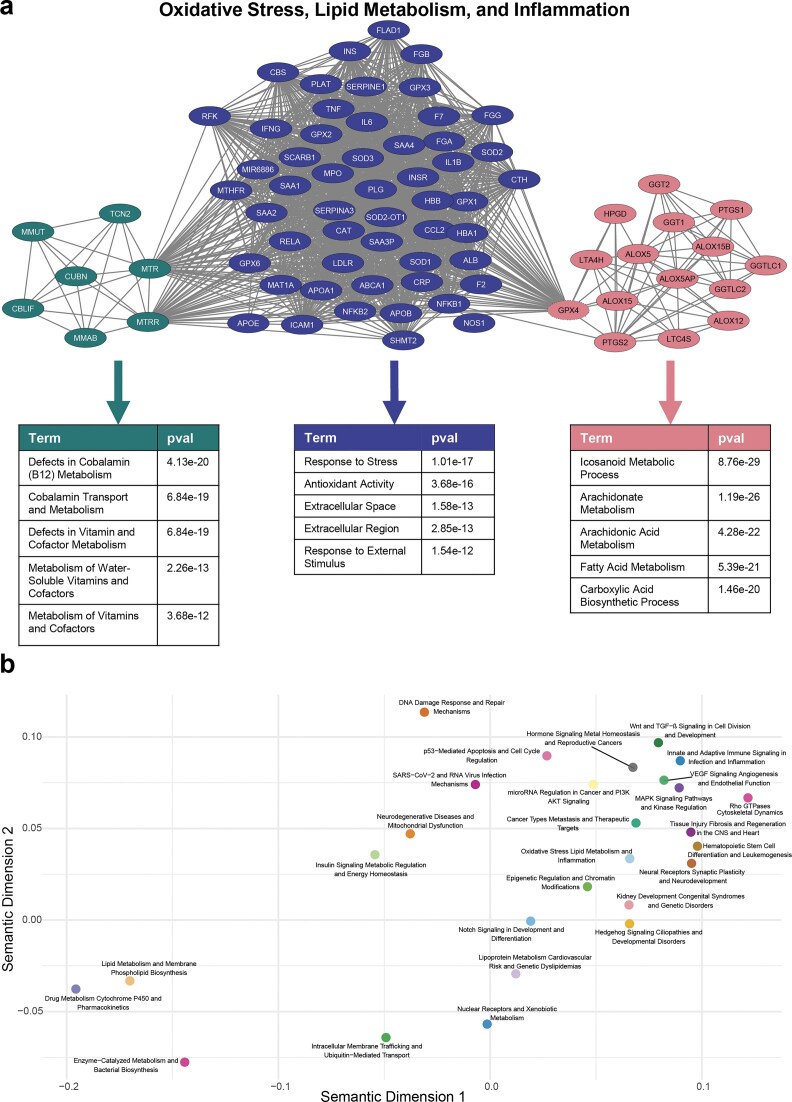
Functional annotation of Louvain-derived modules across topic-specific networks. (**A**) Example of a topic-specific network for “Oxidative Stress, Lipid Metabolism, and Inflammation,” the smallest network in the dataset (75 genes). Louvain clustering identifies three distinct modules, highlighted in different colors: the green module is functionally enriched for vitamin metabolism, the red module for fatty acid metabolism with a focus on inflammation, and the purple module for cellular stress response. (**B**) Semantic similarity map of the 27 topics based on the 100 most significant GO:BP terms of their modules. Each point represents one topic-specific network, positioned using multidimensional scaling of semantic distances among enriched terms.

Despite substantial variation in size and connectivity across topic networks, modular structure was consistently detected. An illustrative example is provided in Fig. 3A, which displays the network “Oxidative Stress, Lipid Metabolism, and Inflammation.” This network comprises 75 genes that are part of intersections among 12 gene sets assigned to this topic, making it the smallest of the 27 topic-specific graphs in terms of contributing documents. Nonetheless, three distinct modules were identified, each representing a subset of genes that exhibit a particular connection pattern within the topic-specific network.

Following module detection, functional characterization was performed using ORA on the genes within each module, utilizing gene sets from GO [25, 26], Reactome [17], and KEGG [18]. The semantic similarity map in Fig. 3B reveals clear separation of topics based on the functional profiles of their annotated modules. Each point represents the centroid of a topic, summarizing its most enriched GO biological process terms. The spatial distribution highlights distinct clusters of biologically related topics: for example, “Lipid Metabolism and Membrane Phospholipid Biosynthesis” appears near “Drug Metabolism Cytochrome P450 and Pharmacokinetics,” reflecting shared metabolic processes, while signaling and development-related topics form a separate cluster encompassing “MAPK Signaling Pathways and Kinase Regulation,” “Wnt and TGF-β Signaling in Cell Division and Development,” and “Innate and Adaptive Immune Signaling in Infection and Inflammation.” This separation reflects the functional coherence and diversity captured by the modular annotations and underscores the biological specificity embedded in each topic’s network structure.

### Centrality and fractality metrics uncover context-dependent importance of genes across topic-specific networks

The structural position of a gene within a topic-specific co-occurrence network provides a proxy for its contextual importance. It is assumed that this topological embedding reflects how prominently a gene contributes to the functional architecture within a given biological theme. To quantify this embedding, a suite of node-level topological metrics was calculated across all topic networks, including betweenness centrality (“bottleneck” genes), eigenvector centrality (hubs of important genes), and LFD (hierarchical regulators). In addition, the robustness of selected metrics was estimated by their variances under perturbations by randomized edge rewiring, capturing the structural stability of a gene’s influence.

**Figure 4.**
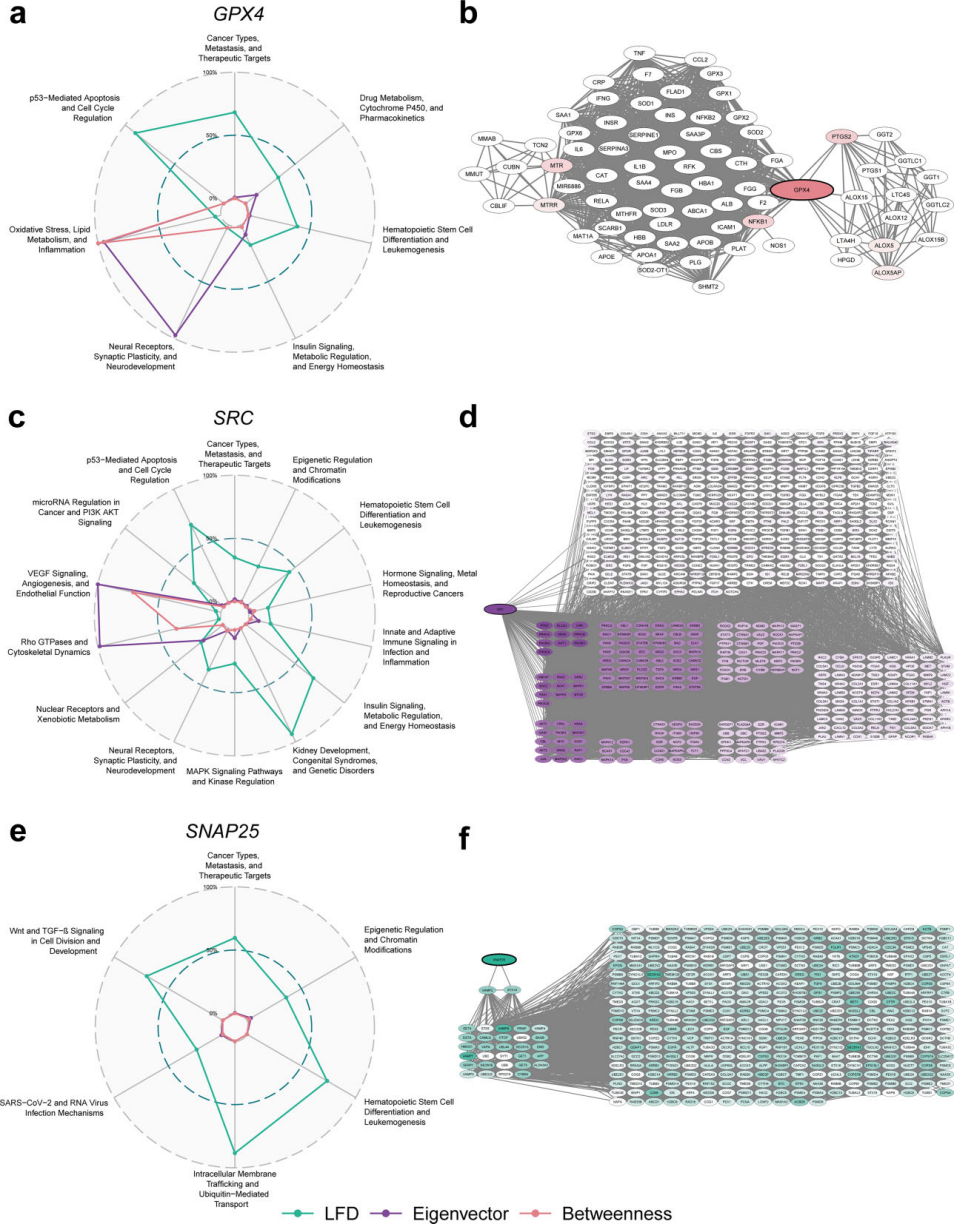
Topological metrics across topic-specific networks reveal context-dependent gene relevance. For each of the three selected metrics, betweenness, eigenvector centrality, and LFD, one representative gene with exceptionally high values in a specific network is shown. The left panels display radar plots of the metric values for the gene across all topic-specific networks, highlighting its contextual specificity; these plots can also be accessed interactively via the CENTRA web application. The right panels depict the local network neighborhood of the gene within the respective topic, colored by topology metric where a more intense color accounts for a higher metric score. (**A, B**) *GPX4* shows high betweenness in the “Oxidative Stress, Lipid Metabolism, and Inflammation” network, acting as a bottleneck bridging two main node communities. (**C, D**) *SRC* is a hub of eigenvector centrality in the network “VEGF Signaling, Angiogenesis, and Endothelial Function,” indicating central influence through connections to other highly central nodes. (**E, F**) *SNAP25* has the highest LFD in “Intracellular Membrane Trafficking and Ubiquitin-Mediated Transport,” embedded in a highly branched neighborhood with exponential growth in local connectivity as distance from *SNAP25* increases linearly.

To illustrate the interpretive value of node-level topological metrics, we now discuss three representative genes that each exhibit a pronounced signal in one of the assessed metrics:

*GPX4* displays exceptionally high betweenness centrality in the network “Oxidative Stress, Lipid Metabolism, and Inflammation” (see Fig. 4A and B), highlighting its role as a topological bottleneck that mediates communication between otherwise weakly connected regions. This elevated betweenness suggests that *GPX4* frequently lies on the shortest paths between other genes, enabling it to coordinate processes across distinct network modules. Consistently, *GPX4* connects modules related to oxidative stress response and fatty acid metabolism (see Fig. 3A), reflecting its functional role as a glutathione peroxidase that reduces lipid hydroperoxides and protects cells from ferroptotic death [32]. Likewise remarkable and consistent is the exceedingly high eigenvector centrality of *GPX4* in the network “Neural Receptor, Synaptic Plasticity, and Neurodevelopment” (see Fig. 4A). This suggests a high relevance of the gene in neural development, function and response to injury. In agreement, *GPX4* upregulation is observed in astrocytes upon brain injury and appears to protect cells from apoptosis [33].

*SRC* displays prominently high eigenvector centrality in the network “VEGF Signaling, Angiogenesis, and Endothelial Function” (see Fig. 4C). This metric reflects not just the number of connections, but the influence of those connections: *SRC* is linked predominantly to other genes with a high eigenvector centrality rather than less central nodes (see Fig. 4D). This positions *SRC* within the network’s core of regulatory influence, suggesting a central role in propagating biological signals through densely connected hubs. This is consistent with *SRC*’s well-characterized function as a nonreceptor tyrosine kinase that integrates growth factor signaling and promotes angiogenic responses, endothelial cell migration, and vascular remodeling, which are hallmark processes of the biological theme captured by this network [34, 35].

*SNAP25* demonstrates a uniquely high LFD within the network representing “Intracellular Membrane Trafficking and Ubiquitin-Mediated Transport” (see Fig. 4E). Unlike classical centrality metrics, LFD captures the local structural complexity around a node, quantifying the density and scaling behavior of its immediate topological environment. *SNAP25* resides in a region where connectivity expands rapidly with distance: from just two direct neighbors to 29 at two steps and 373 at three steps corresponding to an exponential neighborhood growth (see Fig. 4F). This fractal-like embedding supports *SNAP25*’s function in vesicle trafficking and membrane fusion, processes that require tight coordination across multiple functional scales within the endomembrane system. While classically associated with synaptic function, *SNAP25* also plays broader roles in intracellular vesicle docking and transport, aligning with its structural position in this network [36].

To infer the functional relevance of genes in understudied or ambiguous contexts, network perturbation analysis was used to identify structurally sensitive nodes whose importance may be concealed under static conditions. Genes of particular interest in this framework are those with low degree, low baseline values in centrality or complexity measures, but high variance under minimal edge rewiring. This pattern suggests that the gene’s topological position is highly responsive to minor structural changes in the network, indicating latent importance in specific contexts and potentially reflecting gaps in existing biological knowledge or annotation. Such genes may serve context-specific roles that are easily disrupted or overlooked in bulk analyses, making topological variance a useful proxy for identifying underexplored functional relevance.

**Figure 5.**
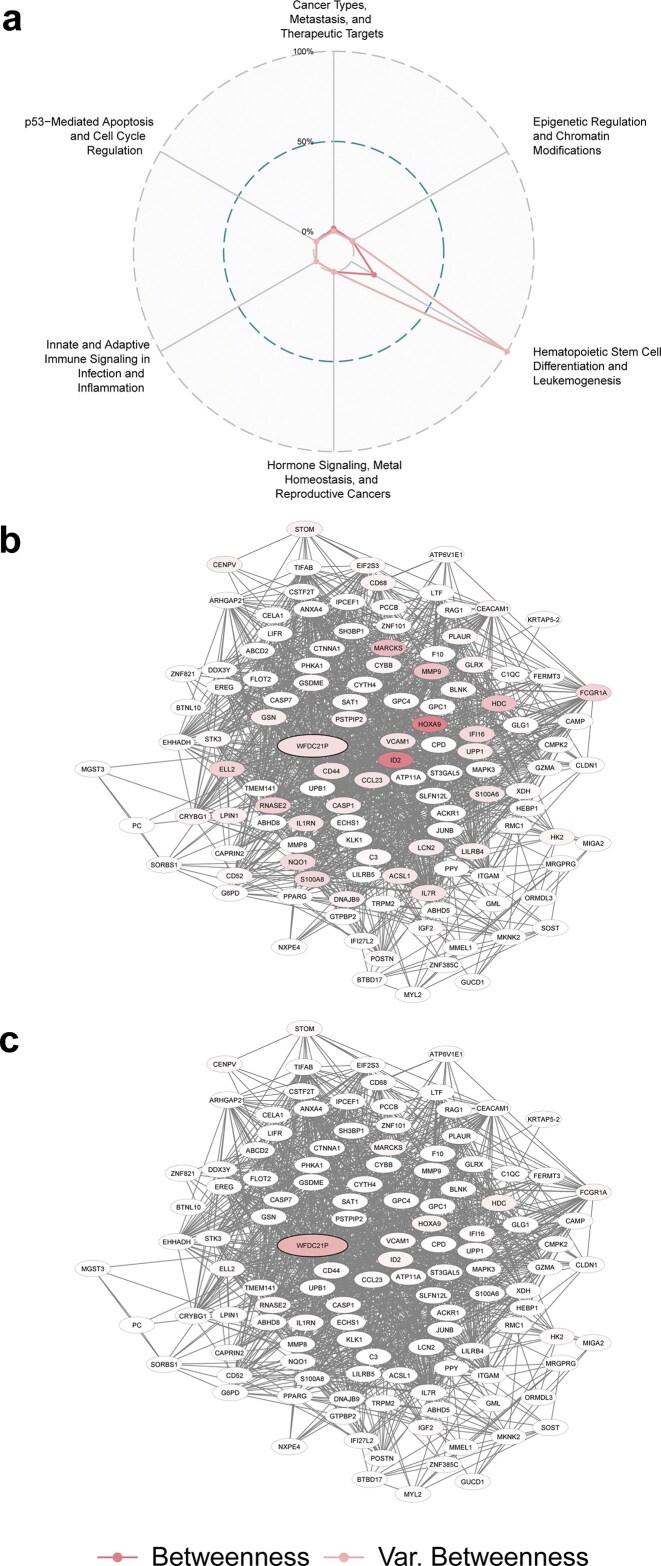
Topological variance reveals latent functional relevance of structurally sensitive genes. While conventional centrality metrics highlight statically embedded hubs, variance-based network perturbation analysis identifies genes whose importance is structurally unstable, i.e. low in static centrality but highly responsive to subtle changes in network topology. This pattern suggests potential functional relevance in specific or under-annotated contexts. WFDC21P, a pseudogene-derived lncRNA, exhibits low betweenness centrality but high betweenness variance in the network “Hematopoietic Stem Cell Differentiation and Leukemogenesis.” (**A**) Radar plot shows metric and variance values across all networks; (**B**) network embedding of WFDC21P colored by betweenness; (**C**) same network colored by variance of betweenness under edge rewiring.

*WFDC21P* (see Fig. 5), a pseudogene-derived long noncoding RNA (lncRNA) with minimal annotation, shows low betweenness centrality but high betweenness variance in “Hematopoietic Stem Cell Differentiation and Leukemogenesis.” This suggests a structurally unstable role, potentially acting as a transient bridge between regulatory modules. As a member of the WFDC family, known for roles in immune modulation and cancer [37–39], *WFDC21P* may function as a noncoding regulator, such as a miRNA sponge or scaffold for RNA-protein complexes. In line, *WFDC21P* has been reported to impact glycolysis and STAT3 signaling in cancer [40]. Its topological volatility may imply latent involvement in epigenetic and transcriptional regulation or the modulation of metabolic control during differentiation or transformation.

The overall single-gene topological metrics were benchmarked against selected Hallmark gene sets from MSigDB. In this analysis, metrics were normalized to intersection size and network size to account for differences in overlap and network scale. The benchmarking revealed particularly elevated metric values for Hallmark gene sets that are strongly aligned with specific topics (e.g. Notch signaling), as well as for sets that are more loosely related, such as the Hallmark gene set Estrogen Response Early in the context of the “Hormone Signaling, Metal Homeostasis, and Reproductive Cancers” network (see Supplementary Fig. S3). These findings extend the single-node-based strategy to established gene sets, indicating that the inferred topological properties capture both direct and indirect thematic associations within curated biological frameworks.

To facilitate broad access to the contextual and topological insights generated by this framework, all analyses have been integrated into CENTRA, an interactive web browser application designed to support exploratory investigation. CENTRA enables researchers to navigate topic-specific networks, inspect the modular architecture of functional annotations, and examine topological properties of individual genes across both well characterized and understudied contexts. Additionally, the app allows users to display global network characteristics, browse enrichment tables linking modules to functional terms from GO [25, 26], Reactome [17], and KEGG [18]. At the gene level, comprehensive topology metrics can be filtered, visualized, and downloaded for all genes across all networks. Furthermore, high-resolution radar plots summarizing gene-specific topological profiles can be interactively generated, customized by selected metrics and networks, and exported for external use. By making these layers of information accessible through an intuitive interface, CENTRA aims to support hypothesis generation, guide experimental prioritization, and encourage a more nuanced understanding of gene function as it emerges from literature-derived, context-aware network structures.

## Discussion

This study presents a scalable and interpretable framework for modeling the contextual relevance of genes based on literature-derived, topic-specific co-occurrence networks. By constructing networks where edges reflect shared occurrence across curated gene sets, and by calculating graph metrics such as centrality, fractality, and perturbation robustness, the approach systematically captures how genes contribute to distinct biological themes. The analysis demonstrates that well-established regulators exhibit interpretable topological signatures, while topological variance highlights potentially understudied genes with likely context-specific roles. To make these results accessible, the browser application CENTRA enables researchers to interactively explore the networks, functional modules, and gene-level metrics presented here, and to generate new hypotheses beyond the original analysis. Unlike conventional approaches, such as ORA and GSEA, which operate on static gene lists and predefined annotations, this framework and its implementation in CENTRA offer a dynamic, topology-aware, and context-sensitive view on potential functions of single genes and proteins.

LDA is a generative probabilistic model designed to identify hidden (i.e. latent) thematic structures within large collections of data. Originally developed for topic modeling in natural language processing, LDA assumes that documents are mixtures of topics, and that topics are distributions over words [15]. By inferring these latent topic structures, LDA can reveal semantic groupings without requiring prior labeling or supervision. In biomedical research, LDA has been applied to organize and analyze large bodies of scientific literature, allowing for the discovery of coherent biological themes across abstracts, full texts, or curated document sets [41–44]. In the present analysis, LDA was employed to cluster the abstracts and descriptions associated with MSigDB C2 gene sets, thereby inferring biologically meaningful topics based on the semantic content of gene set documents rather than pre-assigned functional labels.

More recently, LDA and related topic modeling approaches have been adapted to single-cell transcriptomic data. Here, the aim is to uncover latent expression programs that define distinct cellular states, differentiation trajectories, or disease microenvironments [45–47]. By interpreting cells as distributions over latent expression programs, and genes as features contributing to these programs (rather than documents as distributions over word topics), LDA can uncover hidden biological processes that govern cellular states, developmental trajectories, or pathological transitions. Although alternative methods such as nonnegative matrix factorization [48, 49], probabilistic latent semantic analysis [50], or embedding-based models such as BERTopic [51, 52] are available, we chose LDA because it is widely established in biomedical topic modeling and produces explicit topic-term and document-topic distributions that align with CENTRA’s knowledge-centric design. Importantly, LDA enables a straightforward mapping of training documents to the topics identified by the same model, which we exploit to link curated gene sets to topics and construct topic-specific co-occurrence networks. The resulting networks exhibit biologically coherent structures at both the module and node levels, with canonical genes such as *SRC* showing the expected prominence. While LDA is stochastic, potential variability in document-topic assignments is attenuated in CENTRA by using undirected, unweighted networks and by restricting classification to documents above a defined confidence threshold. Through this approach, CENTRA enables researchers to explore gene function within dynamically inferred thematic landscapes, bridging the strengths of probabilistic topic modeling and topological network analysis.

While LDA provides a useful probabilistic framework for clustering based on textual content, it is not the only method available for organizing functional biological information into coherent themes. Semantic similarity measures, particularly those based on GO [25, 26], offer an alternative approach by quantifying the functional relatedness between biological terms based on the structure of curated ontologies. GoSemSim [28] is a widely used tool for this purpose. It computes the semantic similarity between GO terms by analyzing their position within the hierarchical GO graph and measuring the degree of shared ancestry between terms. Rather than relying on direct gene overlap, GoSemSim [28] evaluates how much biological meaning is shared between two processes based on their common ancestors, thus allowing for the comparison of distinct but related annotations.

In the present study, GoSemSim [28] was employed to map the functional relationships between topic-specific networks. Specifically, for each topic, the most significantly enriched GO:BP terms were extracted from module-level annotations, and pairwise semantic similarities were calculated. Multidimensional scaling of these similarities allowed the construction of a semantic landscape that visualizes how topics are positioned relative to one another based on their biological content. This analysis was performed to illustrate that the topic-specific gene co-occurrence networks, constructed via latent topic modeling and gene set co-occurrence, lead to functionally distinct assemblies. The centroids representing each topic were computed based on the semantic coordinates of their top enriched GO terms, providing a summary view of the biological coherence and separation achieved by the underlying network construction.

While GoSemSim [28] provides an effective means to compare the semantic proximity of biological processes, the present framework and CENTRA extend beyond purely functional similarity. By embedding genes into topic-specific co-occurrence networks and analyzing their topological properties, the analysis captures not only what biological processes are enriched, but also how individual genes are structurally organized within these contexts. This integration of functional annotation and network architecture enables a deeper, context-aware exploration of gene function that cannot be achieved through semantic similarity measures alone.

Other tools have previously explored the intersection of literature mining and gene function interpretation yet differ notably in scope and resolution. GeneTopics [53], for instance, applies topic modeling to gene-associated literature to identify dominant semantic themes and assign relevancy scores, offering an alternative to curated annotations through automated topic-based summaries. However, its primary focus lies in summarizing gene sets through representative keywords and literature, without modeling network structure or providing single-gene metrics. Similarly, the Gene-set Cohesion Analysis Tool (GCAT) [54] employs Latent Semantic Indexing to assess the functional coherence of gene sets, generating cohesion scores based on textual similarity. While it displays network graphs, its core utility lies in measuring the overall functional coherence of the gene set as a whole, rather than providing granular topological metrics, such as centrality or fractality, for individual genes to characterize their precise roles within distinct, topic-specific contexts. While effective for evaluating overall group consistency, GCAT does not offer structural insights at the gene level or explore context-dependent roles across multiple biological themes. Another framework for deriving gene-function relations, ProtSemNet [55], constructs bipartite semantic networks with proteins and topics as vertices to evaluate protein group coherence, capturing topic-level bridging patterns qualitatively. Yet, it lacks the granularity needed to assess the topological relevance of individual genes within diverse functional contexts, as its primary metrics are focused on the overall coherence of a gene set as measured by shortest-distance subgraphs rather than a suite of systematic node-level topological measures for individual genes. In contrast, CENTRA directly embeds genes into topic-specific co-occurrence networks, quantifies their topological roles using a comprehensive set of graph metrics, and integrates this with functional enrichment to support hypothesis generation at single-gene resolution. This combination of semantic modeling and structural profiling offers a flexible and interpretable framework for exploring gene function beyond the group-level focus of earlier approaches.

Although the CENTRA framework uses modular enrichment on networks constructed from annotated gene sets, the analytical approach does not reiterate or recycle the original annotations. Instead, it introduces two independent abstraction layers: first, gene sets are clustered based on the semantic content of their reporting abstracts using unsupervised topic modeling, not by curated functional categories; second, co-occurrence networks are formed purely from structural gene intersections within each topic, without reference to any annotation. Louvain clustering is then applied to the resulting graphs—again independently of functional input—and enrichment is conducted only afterward using a distinct set of knowledge resources (GO [25, 26], Reactome [17], KEGG [18]). Functional patterns thus emerge post hoc from structurally inferred gene modules and reflect the organization of genes in a latent, topic-specific context. This decoupling between source, structure, and annotation allows the recovery of both known and novel associations without direct reuse of the original semantic content.

While the integration of functional annotation with topic-specific co-occurrence networks offers a scalable view of gene contextuality, some limitations of the current framework must be acknowledged. First, the current analysis is based on gene sets and abstracts from curated databases, limiting coverage to documented biological processes. Although incorporating additional MSigDB [11, 12] collections such as Hallmark, Immune Signatures, or GO-derived sets could broaden thematic diversity, the resulting networks would still be constrained by literature availability and reporting bias. However, the CENTRA framework is designed to uncover latent, hidden structures within the existing body of knowledge, supporting hypothesis generation even in contexts where explicit annotations are sparse. Second, topological metric robustness was assessed by random edge rewiring, capturing general sensitivity of node-level betweenness centrality but not modeling regulatory changes such as differential expression, alternative splicing, or post-translational modification. More biologically realistic perturbation, such as introducing pseudo-nodes, assigning edge weights, or using directed interactions, could better reflect biological dynamics. Yet, such modifications would fundamentally alter the intuitive simplicity of topic-specific gene co-occurrence networks, potentially obscuring their accessibility for exploratory hypothesis generation, which remains a primary aim of the present framework. Third, the quality of gene set annotations is heterogeneous. To reduce semantic noise, the analysis included only those abstracts that could be confidently assigned to topics. While this approach inevitably excludes some biologically meaningful but ambiguously described gene sets, stricter inclusion criteria were favored to maximize clarity and robustness in the interpretation of topological measures across topics. By emphasizing precision over inclusivity, the resulting networks better support coherent functional interpretation at the cost of descriptive depth. Fourth, empirical validation and integration of experimental data remain open challenges. In this study, “context specificity” is defined by the thematic focus of a topic, derived from latent biomedical themes identified by LDA across curated gene sets. While this differs from experimental condition-specificity, it may offer a structured, knowledge-based approximation of biological contexts in which genes recurrently co-occur. The current analysis provides opportunities for plausible hypotheses regarding context-dependent gene importance, particularly for poorly annotated genes, based on their structural properties and perturbation sensitivity within topic-specific gene co-occurrence networks. However, these insights remain computational inferences and require experimental validation.

Adapting the framework to incorporate user-provided experimental data would not constitute a simple extension of CENTRA. It would necessitate the construction of fundamentally different network architectures, moving from unweighted, topic-specific gene co-occurrence networks toward weighted, data-integrated systems. Such an approach would be incommensurable with the current model, which prioritizes interpretability, intuitive navigation, and consistency with curated biological knowledge. Moreover, customized gene sets typically lack the descriptive annotation required to assign them to topic-specific networks or to reliably connect them to latent biomedical topics identified within the current framework. For such sets, contextualization through enrichment analyses (e.g. ORA or GSEA) may be more meaningful, whereas CENTRA as presented here is deliberately designed as a knowledge-centered exploration platform, providing information at the single-gene level and supporting hypothesis generation rather than direct experimental integration. Furthermore, the current framework relies exclusively on human gene sets, which limits comparability with established model organism studies and xenografting experiments. Future developments may therefore include the introduction of edge weighting, bipartite graph representations, or the construction of networks for non-human species, thereby broadening the applicability of the framework and facilitating integration with experimental data.

In summary, the present analysis and CENTRA establish a flexible, interpretable, and accessible framework for context-aware exploration of gene function. By integrating semantic topic modeling with topological structure, the approach introduces a novel analytic dimension that moves beyond static gene lists toward literature-derived, network-based representations of biological knowledge. As functional genomics continues to scale in complexity, tools like CENTRA offer a conceptual bridge between curated domain knowledge and data-driven discovery. Looking ahead, this framework contributes to the groundwork for integrative models that combine prior knowledge with experimental data, enabling future platforms to dynamically synthesize context, structure, and function into interpretable biological insights. CENTRA thus marks a step toward a new class of exploratory tools that treat gene function not as fixed annotation, but as an emergent property of particular biological contexts.

## Supplementary Material

lqaf196_Supplemental_Files

## Data Availability

CENTRA is publicly accessible without login and free of charge at https://ngs-info.medizin.uni-halle.de/shiny/CENTRA/.
