# Supplementary material for: CENTRA: knowledge-based gene contextuality graphs reveal functional master regulators by centrality and fractality
Source: NAR Genom Bioinform. 2025 Dec 19;7(4):lqaf196. doi: 10.1093/nargab/lqaf196 (PMC12714692; doi:10.1093/nargab/lqaf196)
Supplement: lqaf196_Supplemental_Files [file lqaf196_supplemental_files.zip › Figures S1-S3.pdf]

Supplementary Figure S1: Selection of the optimal number of topics in LDA models. Jensen-Shannon Information Divergence across different numbers of topics k. Models with higher divergence values indicate stronger separability of topics. The shaded area marks the range of topic numbers considered in detail. Based on peak performance and subsequent classification stability, a model with k = 27 topics was selected for downstream analyses.

Supplementary Figure S2: Principal Component Analysis (PCA) of the document-topic assignment matrix. Each dot in the PCA represents one document, colored by its dominant topic, and positioned based on the full probability distribution of this particular document across all 27 topics. Ellipses indicate 95% confidence intervals for each topic cluster.

Supplementary Figure S3: Normalized network metrics for Hallmark gene sets across topic-specific networks. For each of 10 Hallmark gene sets, four node-level measures were evaluated in 27 topic-specific networks: betweenness centrality, eigenvector centrality, local fractal dimension, and variance of betweenness centrality. All values were normalized to intersection size and network size of the respective topic-specific network to allow cross-topic comparison. In each case, Hallmark gene sets reach the highest scores in networks functionally related to their biological theme, reflecting coherent associations between topics and curated gene sets.

Supplementary File S1: PubMed IDs of publications included in topic modeling.

Supplementary File S2: Custom stop words used for preprocessing in topic modeling.

Supplementary File S3: Top 1,000 β-weights of tokens per topic for k = 27.

Supplementary Table S1: Topological properties of the 27 topic-specific gene co-occurrence networks. Each network was constructed by identifying shared genes across all pairwise combinations of gene sets within a given topic.

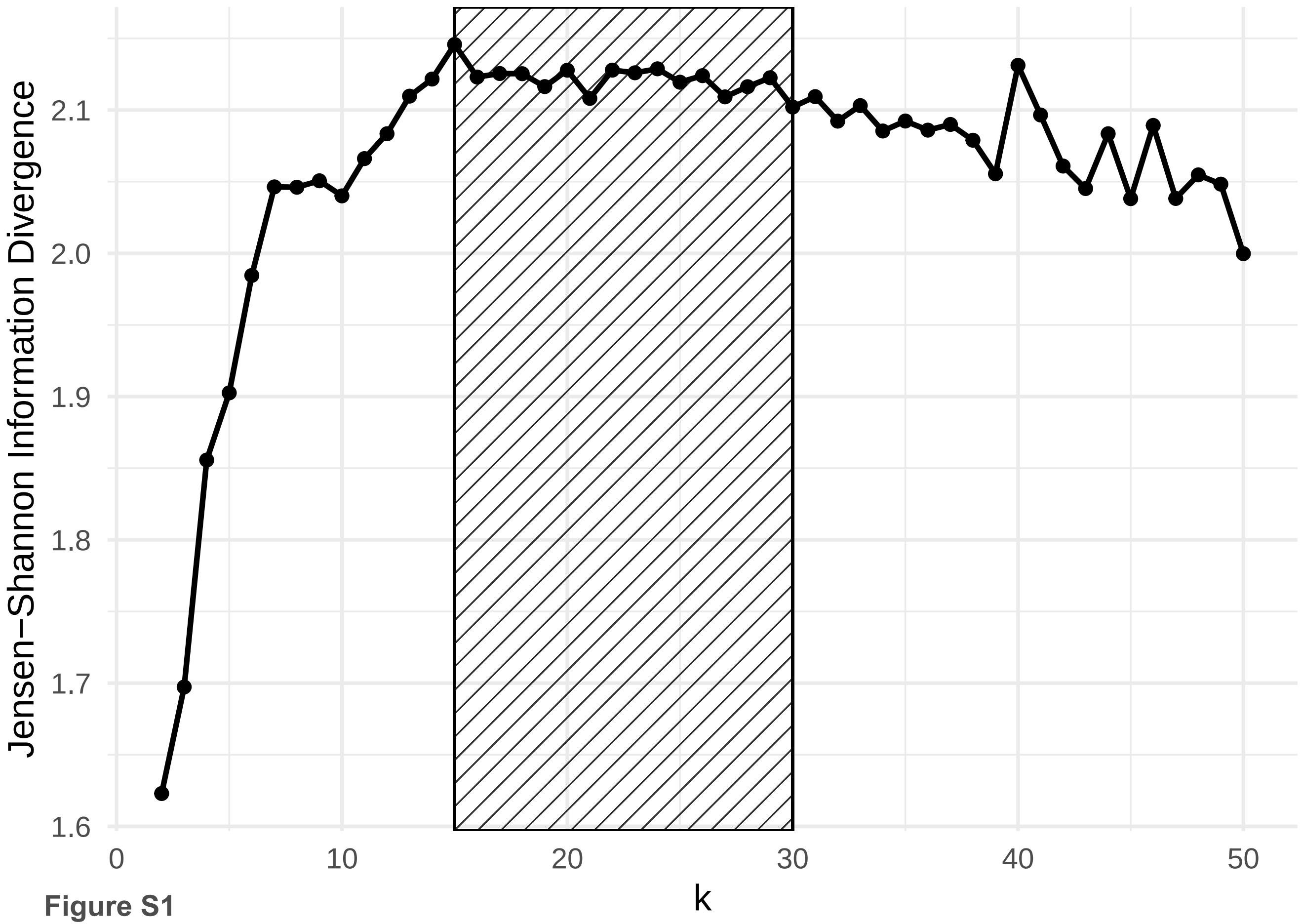
Jensen−Shannon Information Divergence
2.1
2.0
1.9
1.8
1.7
1.6
0
10
20
30
40
50
k

Figure S1

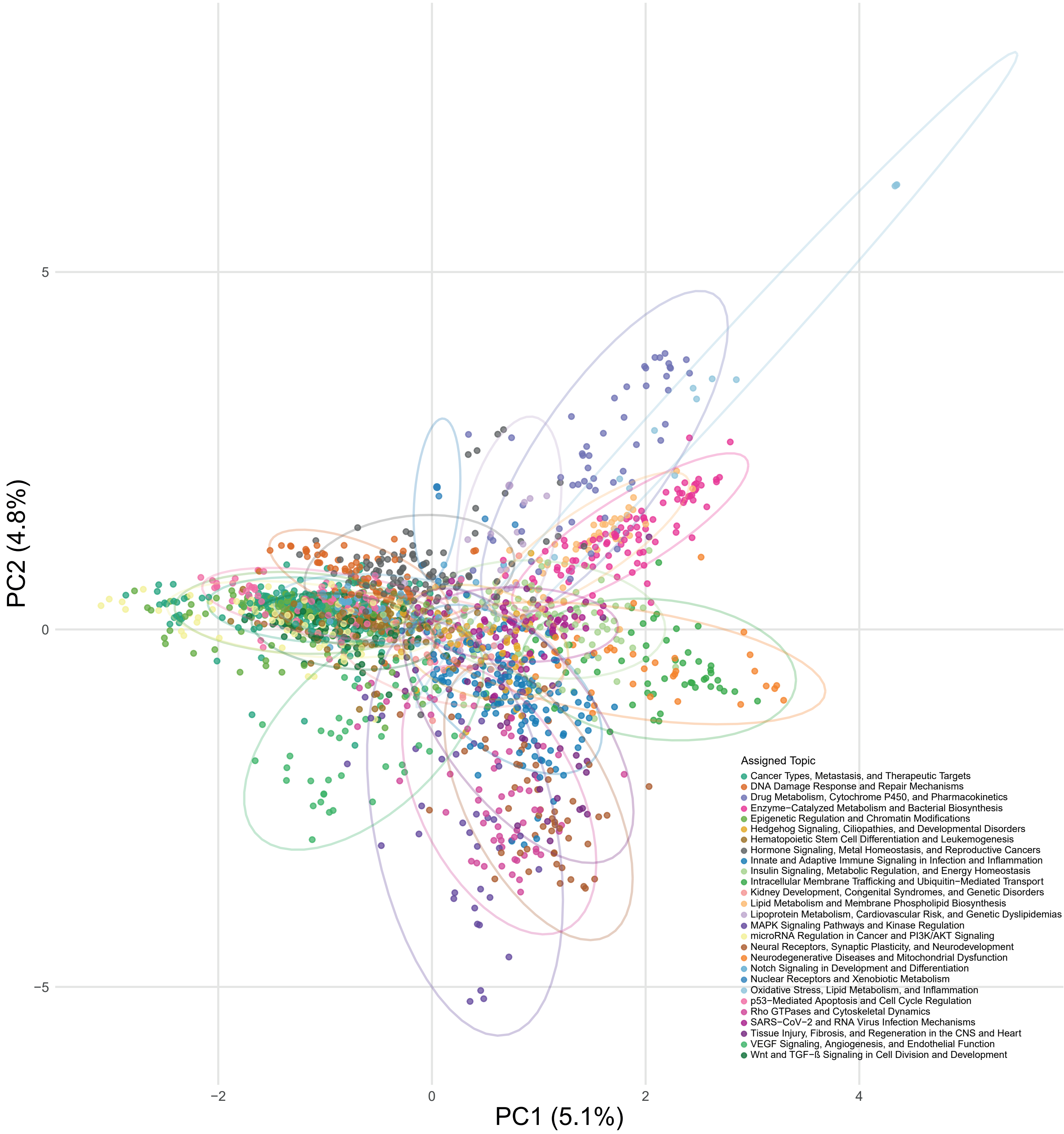
PC2 (4.8%)
PC1 (5.1%)
5
0
−5
−2
0
2
4
Assigned Topic
Cancer Types, Metastasis, and Therapeutic Targets
DNA Damage Response and Repair Mechanisms
Drug Metabolism, Cytochrome P450, and Pharmacokinetics
Enzyme−Catalyzed Metabolism and Bacterial Biosynthesis
Epigenetic Regulation and Chromatin Modifications
Hedgehog Signaling, Ciliopathies, and Developmental Disorders
Hematopoietic Stem Cell Differentiation and Leukemogenesis
Hormone Signaling, Metal Homeostasis, and Reproductive Cancers
Innate and Adaptive Immune Signaling in Infection and Inflammation
Insulin Signaling, Metabolic Regulation, and Energy Homeostasis
Intracellular Membrane Trafficking and Ubiquitin−Mediated Transport
Kidney Development, Congenital Syndromes, and Genetic Disorders
Lipid Metabolism and Membrane Phospholipid Biosynthesis
Lipoprotein Metabolism, Cardiovascular Risk, and Genetic Dyslipidemias
MAPK Signaling Pathways and Kinase Regulation
microRNA Regulation in Cancer and PI3K/AKT Signaling
Neural Receptors, Synaptic Plasticity, and Neurodevelopment
Neurodegenerative Diseases and Mitochondrial Dysfunction
Notch Signaling in Development and Differentiation
Nuclear Receptors and Xenobiotic Metabolism
Oxidative Stress, Lipid Metabolism, and Inflammation
p53−Mediated Apoptosis and Cell Cycle Regulation
Rho GTPases and Cytoskeletal Dynamics
SARS−CoV−2 and RNA Virus Infection Mechanisms
Tissue Injury, Fibrosis, and Regeneration in the CNS and Heart
VEGF Signaling, Angiogenesis, and Endothelial Function
Wnt and TGF−ß Signaling in Cell Division and Development

Figure S2

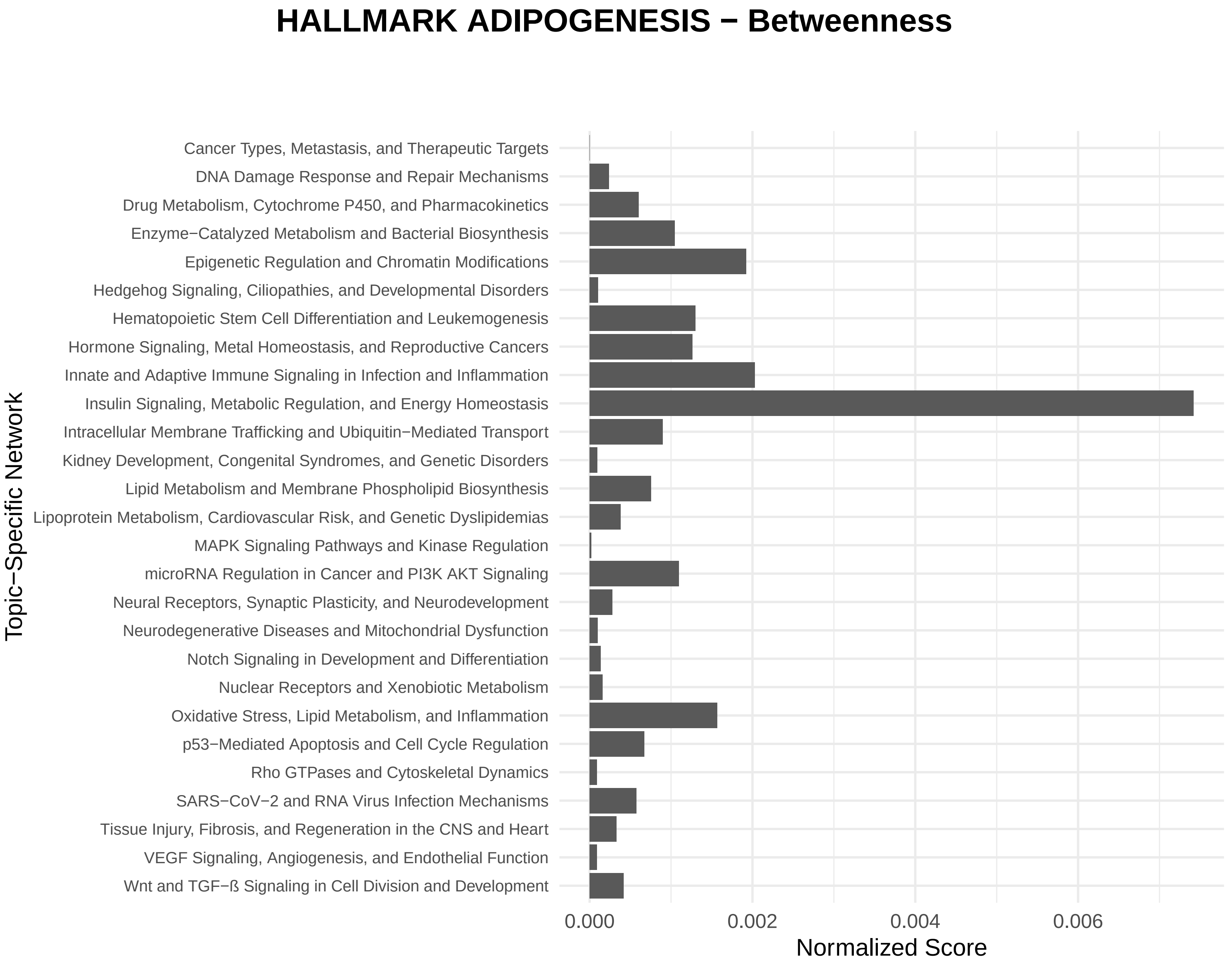
HALLMARK ADIPOGENESIS − Betweenness
Cancer Types, Metastasis, and Therapeutic Targets
DNA Damage Response and Repair Mechanisms
Drug Metabolism, Cytochrome P450, and Pharmacokinetics
Enzyme−Catalyzed Metabolism and Bacterial Biosynthesis
Epigenetic Regulation and Chromatin Modifications
Hedgehog Signaling, Ciliopathies, and Developmental Disorders
Hematopoietic Stem Cell Differentiation and Leukemogenesis
Hormone Signaling, Metal Homeostasis, and Reproductive Cancers
Innate and Adaptive Immune Signaling in Infection and Inflammation
Insulin Signaling, Metabolic Regulation, and Energy Homeostasis
Intracellular Membrane Trafficking and Ubiquitin−Mediated Transport
Kidney Development, Congenital Syndromes, and Genetic Disorders
Lipid Metabolism and Membrane Phospholipid Biosynthesis
Lipoprotein Metabolism, Cardiovascular Risk, and Genetic Dyslipidemias
MAPK Signaling Pathways and Kinase Regulation
microRNA Regulation in Cancer and PI3K AKT Signaling
Neural Receptors, Synaptic Plasticity, and Neurodevelopment
Neurodegenerative Diseases and Mitochondrial Dysfunction
Notch Signaling in Development and Differentiation
Nuclear Receptors and Xenobiotic Metabolism
Oxidative Stress, Lipid Metabolism, and Inflammation
p53−Mediated Apoptosis and Cell Cycle Regulation
Rho GTPases and Cytoskeletal Dynamics
SARS−CoV−2 and RNA Virus Infection Mechanisms
Tissue Injury, Fibrosis, and Regeneration in the CNS and Heart
VEGF Signaling, Angiogenesis, and Endothelial Function
Wnt and TGF−ß Signaling in Cell Division and Development
Topic−Specific Network
0.000
0.002
0.004
0.006
Normalized Score

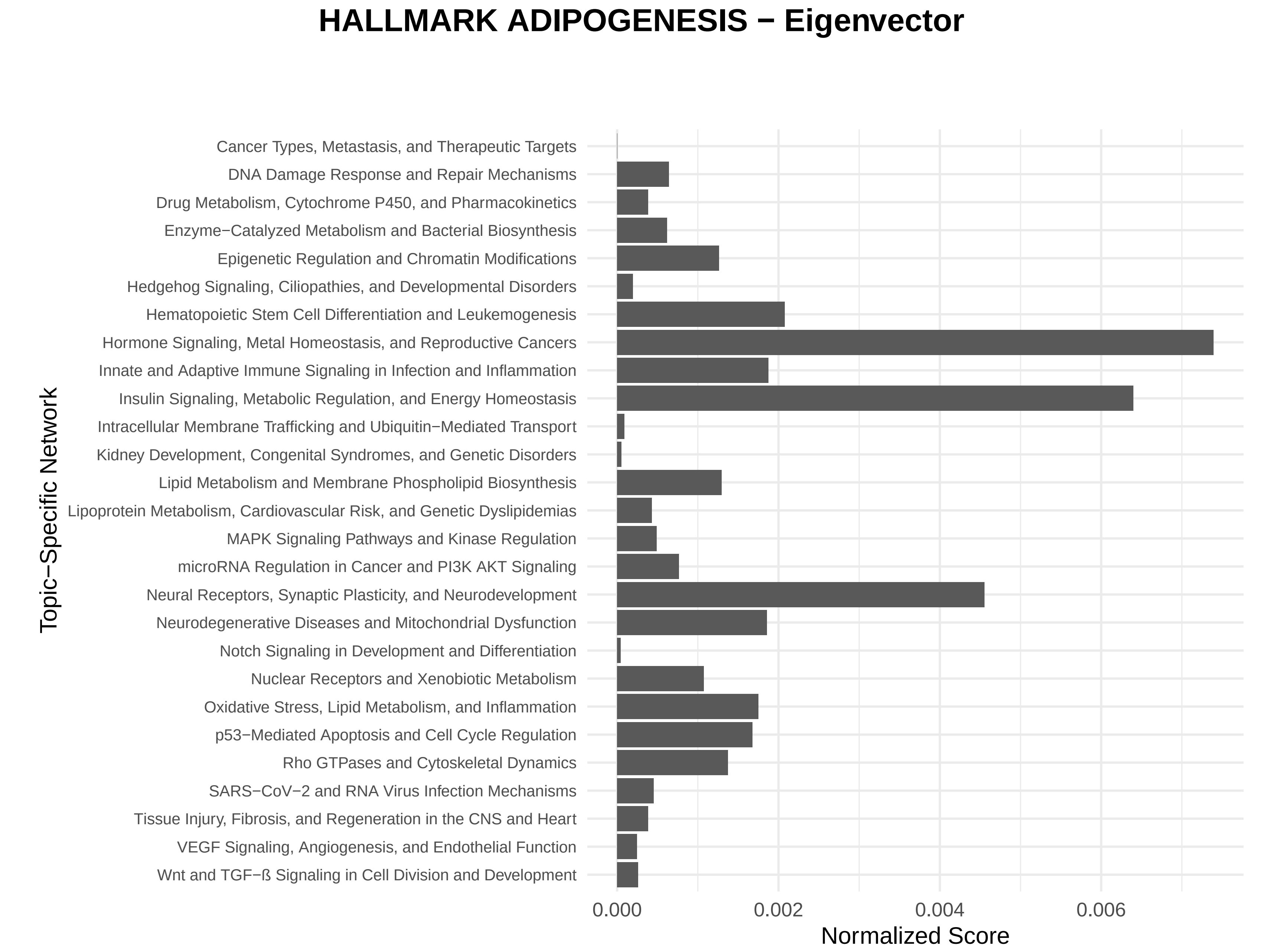
HALLMARK ADIPOGENESIS − Eigenvector
Topic−Specific Network
Cancer Types, Metastasis, and Therapeutic Targets
DNA Damage Response and Repair Mechanisms
Drug Metabolism, Cytochrome P450, and Pharmacokinetics
Enzyme−Catalyzed Metabolism and Bacterial Biosynthesis
Epigenetic Regulation and Chromatin Modifications
Hedgehog Signaling, Ciliopathies, and Developmental Disorders
Hematopoietic Stem Cell Differentiation and Leukemogenesis
Hormone Signaling, Metal Homeostasis, and Reproductive Cancers
Innate and Adaptive Immune Signaling in Infection and Inflammation
Insulin Signaling, Metabolic Regulation, and Energy Homeostasis
Intracellular Membrane Trafficking and Ubiquitin−Mediated Transport
Kidney Development, Congenital Syndromes, and Genetic Disorders
Lipid Metabolism and Membrane Phospholipid Biosynthesis
Lipoprotein Metabolism, Cardiovascular Risk, and Genetic Dyslipidemias
MAPK Signaling Pathways and Kinase Regulation
microRNA Regulation in Cancer and PI3K AKT Signaling
Neural Receptors, Synaptic Plasticity, and Neurodevelopment
Neurodegenerative Diseases and Mitochondrial Dysfunction
Notch Signaling in Development and Differentiation
Nuclear Receptors and Xenobiotic Metabolism
Oxidative Stress, Lipid Metabolism, and Inflammation
p53−Mediated Apoptosis and Cell Cycle Regulation
Rho GTPases and Cytoskeletal Dynamics
SARS−CoV−2 and RNA Virus Infection Mechanisms
Tissue Injury, Fibrosis, and Regeneration in the CNS and Heart
VEGF Signaling, Angiogenesis, and Endothelial Function
Wnt and TGF−ß Signaling in Cell Division and Development
0.000
0.002
0.004
0.006
Normalized Score

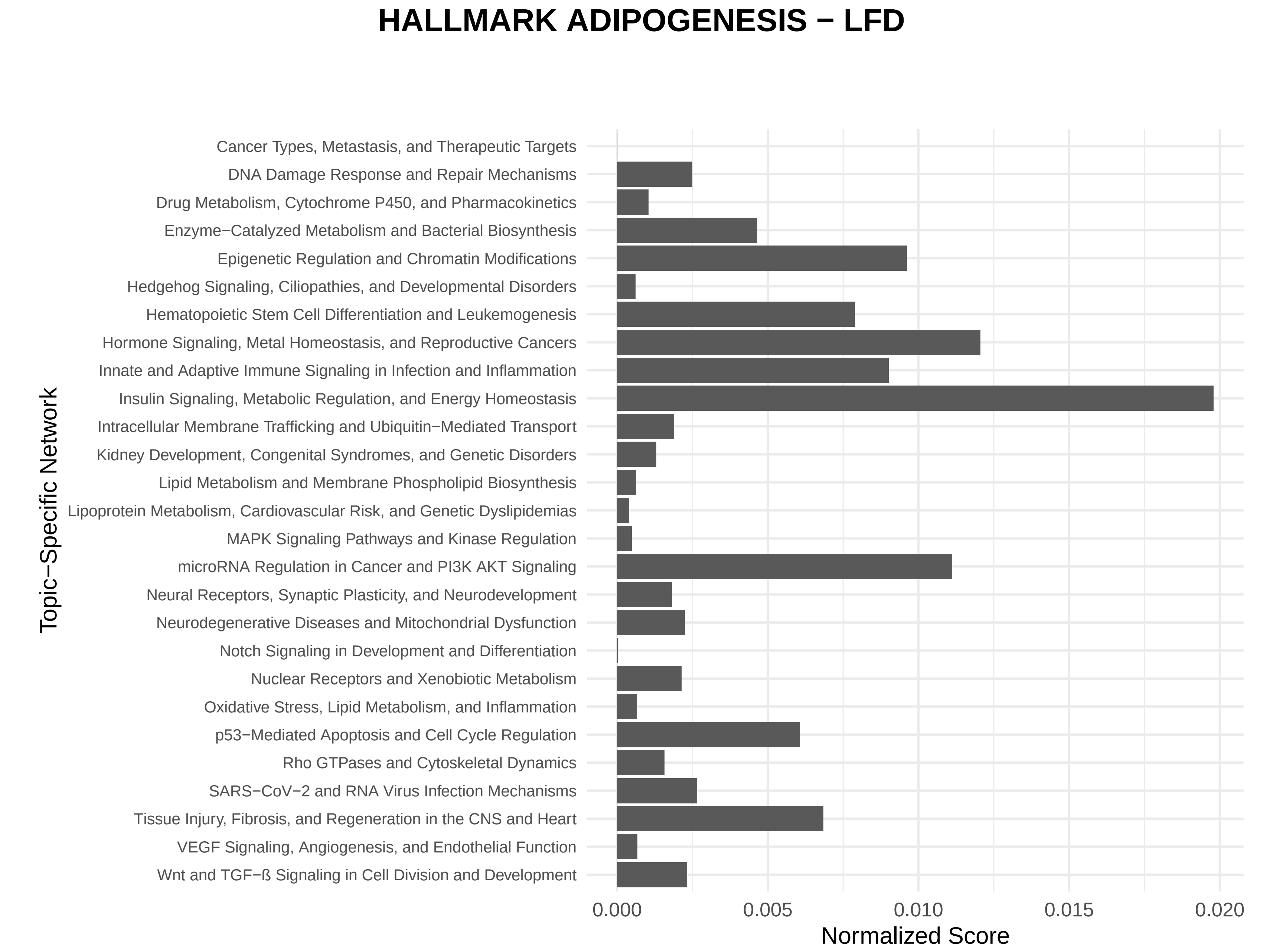
HALLMARK ADIPOGENESIS − LFD
Topic−Specific Network
Cancer Types, Metastasis, and Therapeutic Targets
DNA Damage Response and Repair Mechanisms
Drug Metabolism, Cytochrome P450, and Pharmacokinetics
Enzyme−Catalyzed Metabolism and Bacterial Biosynthesis
Epigenetic Regulation and Chromatin Modifications
Hedgehog Signaling, Ciliopathies, and Developmental Disorders
Hematopoietic Stem Cell Differentiation and Leukemogenesis
Hormone Signaling, Metal Homeostasis, and Reproductive Cancers
Innate and Adaptive Immune Signaling in Infection and Inflammation
Insulin Signaling, Metabolic Regulation, and Energy Homeostasis
Intracellular Membrane Trafficking and Ubiquitin−Mediated Transport
Kidney Development, Congenital Syndromes, and Genetic Disorders
Lipid Metabolism and Membrane Phospholipid Biosynthesis
Lipoprotein Metabolism, Cardiovascular Risk, and Genetic Dyslipidemias
MAPK Signaling Pathways and Kinase Regulation
microRNA Regulation in Cancer and PI3K AKT Signaling
Neural Receptors, Synaptic Plasticity, and Neurodevelopment
Neurodegenerative Diseases and Mitochondrial Dysfunction
Notch Signaling in Development and Differentiation
Nuclear Receptors and Xenobiotic Metabolism
Oxidative Stress, Lipid Metabolism, and Inflammation
p53−Mediated Apoptosis and Cell Cycle Regulation
Rho GTPases and Cytoskeletal Dynamics
SARS−CoV−2 and RNA Virus Infection Mechanisms
Tissue Injury, Fibrosis, and Regeneration in the CNS and Heart
VEGF Signaling, Angiogenesis, and Endothelial Function
Wnt and TGF−ß Signaling in Cell Division and Development
0.000
0.005
0.010
0.015
0.020
Normalized Score

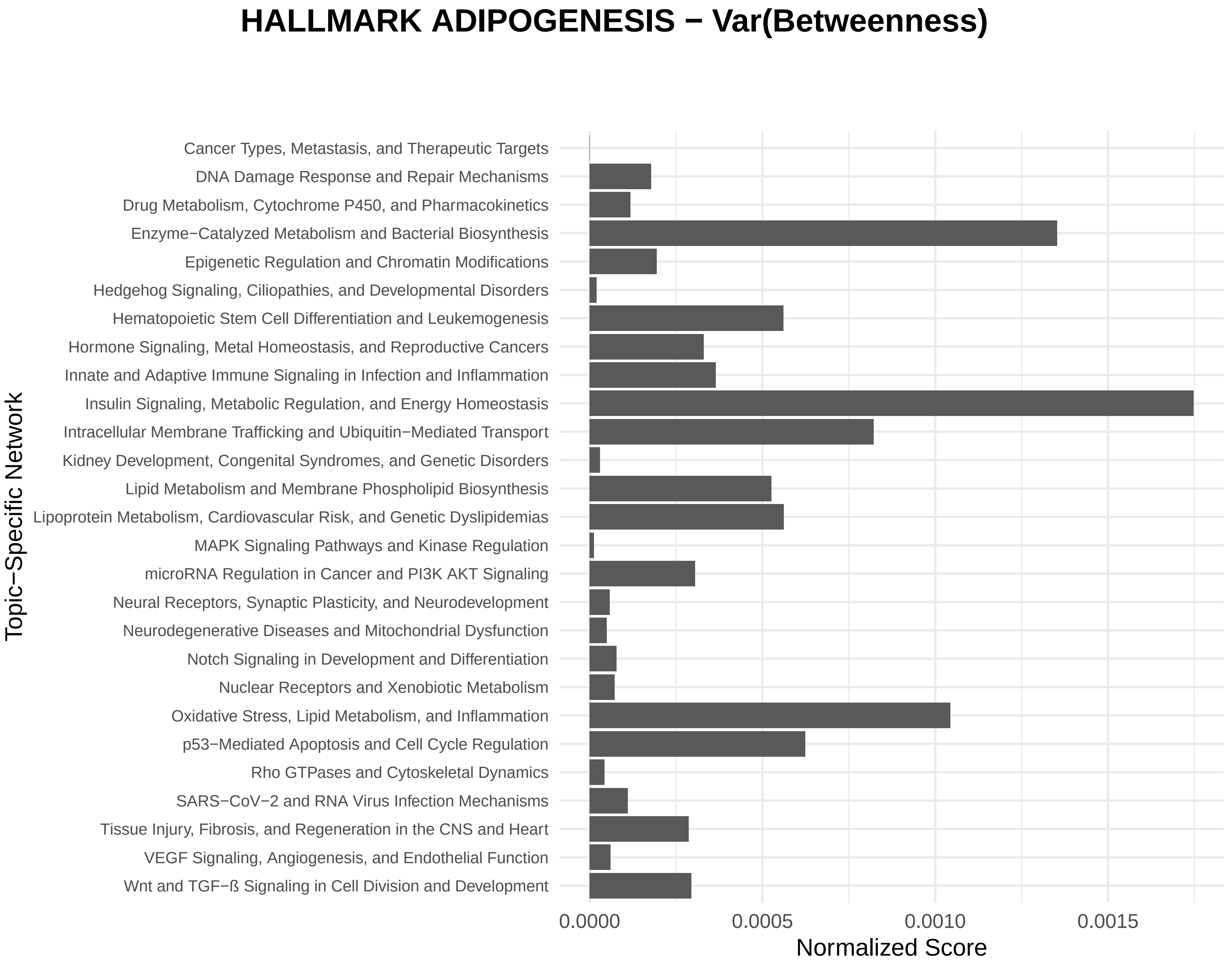
HALLMARK ADIPOGENESIS − Var(Betweenness)
Topic−Specific Network
Cancer Types, Metastasis, and Therapeutic Targets
DNA Damage Response and Repair Mechanisms
Drug Metabolism, Cytochrome P450, and Pharmacokinetics
Enzyme−Catalyzed Metabolism and Bacterial Biosynthesis
Epigenetic Regulation and Chromatin Modifications
Hedgehog Signaling, Ciliopathies, and Developmental Disorders
Hematopoietic Stem Cell Differentiation and Leukemogenesis
Hormone Signaling, Metal Homeostasis, and Reproductive Cancers
Innate and Adaptive Immune Signaling in Infection and Inflammation
Insulin Signaling, Metabolic Regulation, and Energy Homeostasis
Intracellular Membrane Trafficking and Ubiquitin−Mediated Transport
Kidney Development, Congenital Syndromes, and Genetic Disorders
Lipid Metabolism and Membrane Phospholipid Biosynthesis
Lipoprotein Metabolism, Cardiovascular Risk, and Genetic Dyslipidemias
MAPK Signaling Pathways and Kinase Regulation
microRNA Regulation in Cancer and PI3K AKT Signaling
Neural Receptors, Synaptic Plasticity, and Neurodevelopment
Neurodegenerative Diseases and Mitochondrial Dysfunction
Notch Signaling in Development and Differentiation
Nuclear Receptors and Xenobiotic Metabolism
Oxidative Stress, Lipid Metabolism, and Inflammation
p53−Mediated Apoptosis and Cell Cycle Regulation
Rho GTPases and Cytoskeletal Dynamics
SARS−CoV−2 and RNA Virus Infection Mechanisms
Tissue Injury, Fibrosis, and Regeneration in the CNS and Heart
VEGF Signaling, Angiogenesis, and Endothelial Function
Wnt and TGF−ß Signaling in Cell Division and Development
0.0000
0.0005
0.0010
0.0015
Normalized Score

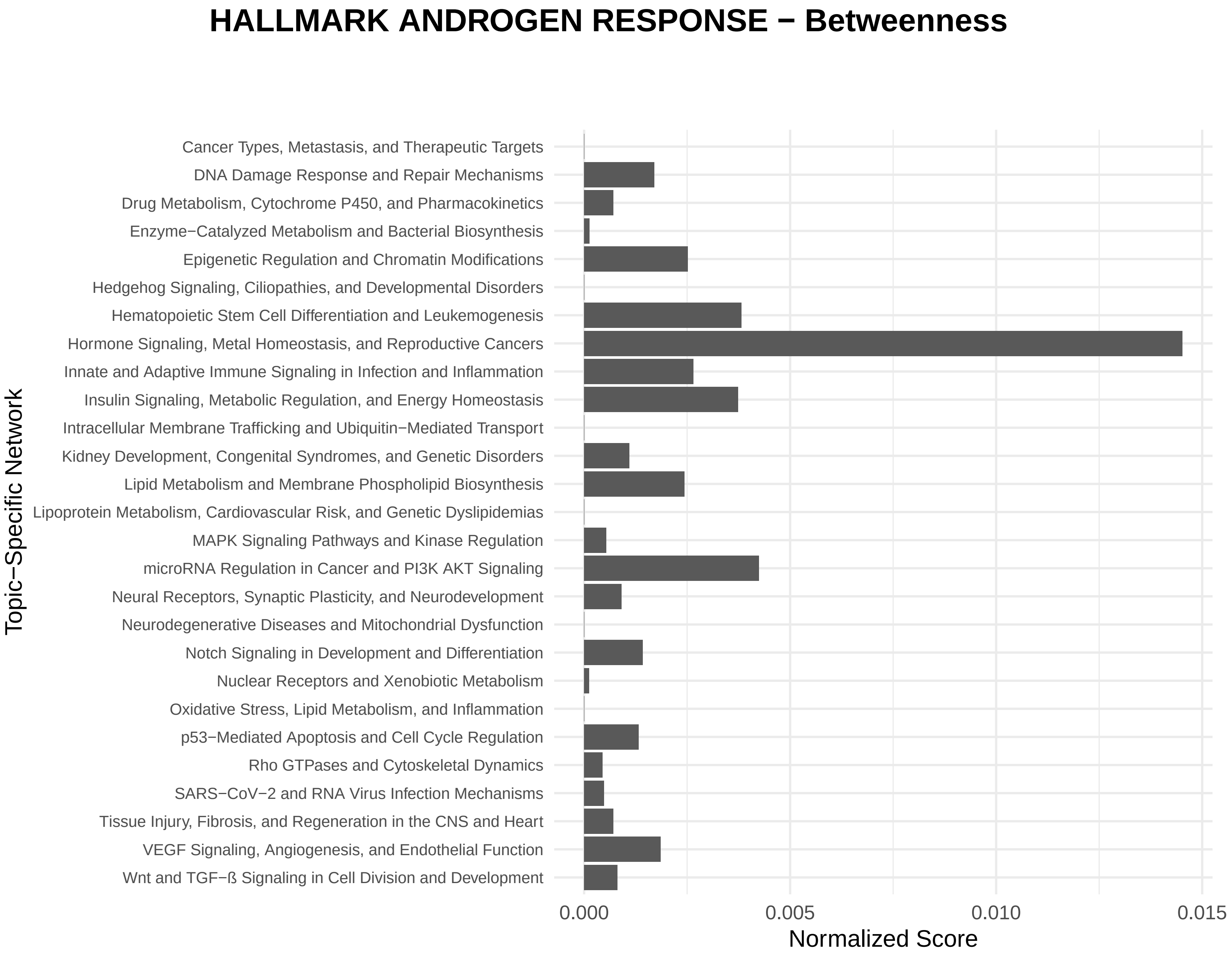
HALLMARK ANDROGEN RESPONSE − Betweenness
Topic−Specific Network
Cancer Types, Metastasis, and Therapeutic Targets
DNA Damage Response and Repair Mechanisms
Drug Metabolism, Cytochrome P450, and Pharmacokinetics
Enzyme−Catalyzed Metabolism and Bacterial Biosynthesis
Epigenetic Regulation and Chromatin Modifications
Hedgehog Signaling, Ciliopathies, and Developmental Disorders
Hematopoietic Stem Cell Differentiation and Leukemogenesis
Hormone Signaling, Metal Homeostasis, and Reproductive Cancers
Innate and Adaptive Immune Signaling in Infection and Inflammation
Insulin Signaling, Metabolic Regulation, and Energy Homeostasis
Intracellular Membrane Trafficking and Ubiquitin−Mediated Transport
Kidney Development, Congenital Syndromes, and Genetic Disorders
Lipid Metabolism and Membrane Phospholipid Biosynthesis
Lipoprotein Metabolism, Cardiovascular Risk, and Genetic Dyslipidemias
MAPK Signaling Pathways and Kinase Regulation
microRNA Regulation in Cancer and PI3K AKT Signaling
Neural Receptors, Synaptic Plasticity, and Neurodevelopment
Neurodegenerative Diseases and Mitochondrial Dysfunction
Notch Signaling in Development and Differentiation
Nuclear Receptors and Xenobiotic Metabolism
Oxidative Stress, Lipid Metabolism, and Inflammation
p53−Mediated Apoptosis and Cell Cycle Regulation
Rho GTPases and Cytoskeletal Dynamics
SARS−CoV−2 and RNA Virus Infection Mechanisms
Tissue Injury, Fibrosis, and Regeneration in the CNS and Heart
VEGF Signaling, Angiogenesis, and Endothelial Function
Wnt and TGF−ß Signaling in Cell Division and Development
0.000
0.005
0.010
0.015
Normalized Score

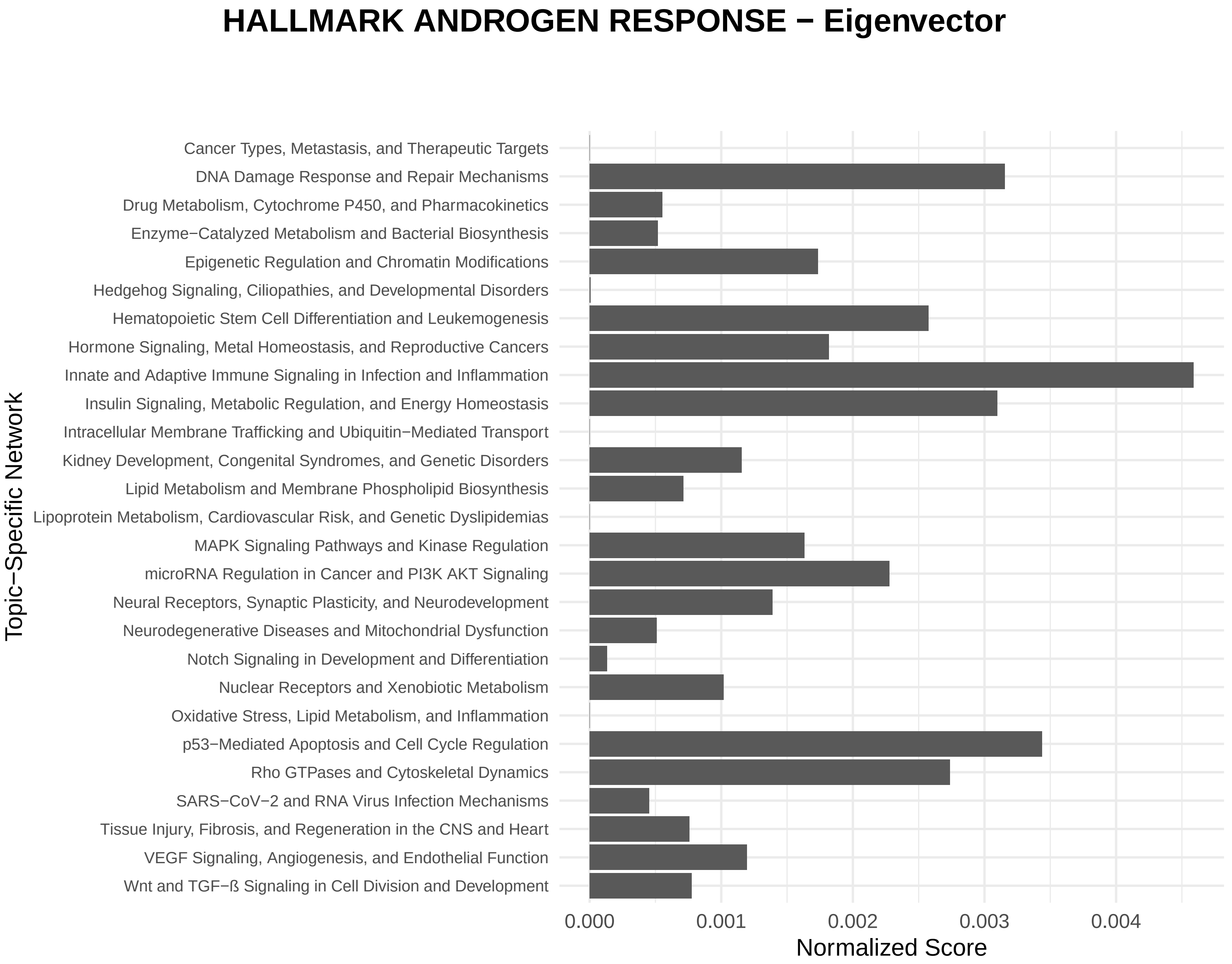
HALLMARK ANDROGEN RESPONSE − Eigenvector
Topic−Specific Network
Cancer Types, Metastasis, and Therapeutic Targets
DNA Damage Response and Repair Mechanisms
Drug Metabolism, Cytochrome P450, and Pharmacokinetics
Enzyme−Catalyzed Metabolism and Bacterial Biosynthesis
Epigenetic Regulation and Chromatin Modifications
Hedgehog Signaling, Ciliopathies, and Developmental Disorders
Hematopoietic Stem Cell Differentiation and Leukemogenesis
Hormone Signaling, Metal Homeostasis, and Reproductive Cancers
Innate and Adaptive Immune Signaling in Infection and Inflammation
Insulin Signaling, Metabolic Regulation, and Energy Homeostasis
Intracellular Membrane Trafficking and Ubiquitin−Mediated Transport
Kidney Development, Congenital Syndromes, and Genetic Disorders
Lipid Metabolism and Membrane Phospholipid Biosynthesis
Lipoprotein Metabolism, Cardiovascular Risk, and Genetic Dyslipidemias
MAPK Signaling Pathways and Kinase Regulation
microRNA Regulation in Cancer and PI3K AKT Signaling
Neural Receptors, Synaptic Plasticity, and Neurodevelopment
Neurodegenerative Diseases and Mitochondrial Dysfunction
Notch Signaling in Development and Differentiation
Nuclear Receptors and Xenobiotic Metabolism
Oxidative Stress, Lipid Metabolism, and Inflammation
p53−Mediated Apoptosis and Cell Cycle Regulation
Rho GTPases and Cytoskeletal Dynamics
SARS−CoV−2 and RNA Virus Infection Mechanisms
Tissue Injury, Fibrosis, and Regeneration in the CNS and Heart
VEGF Signaling, Angiogenesis, and Endothelial Function
Wnt and TGF−ß Signaling in Cell Division and Development
0.000
0.001
0.002
0.003
0.004
Normalized Score

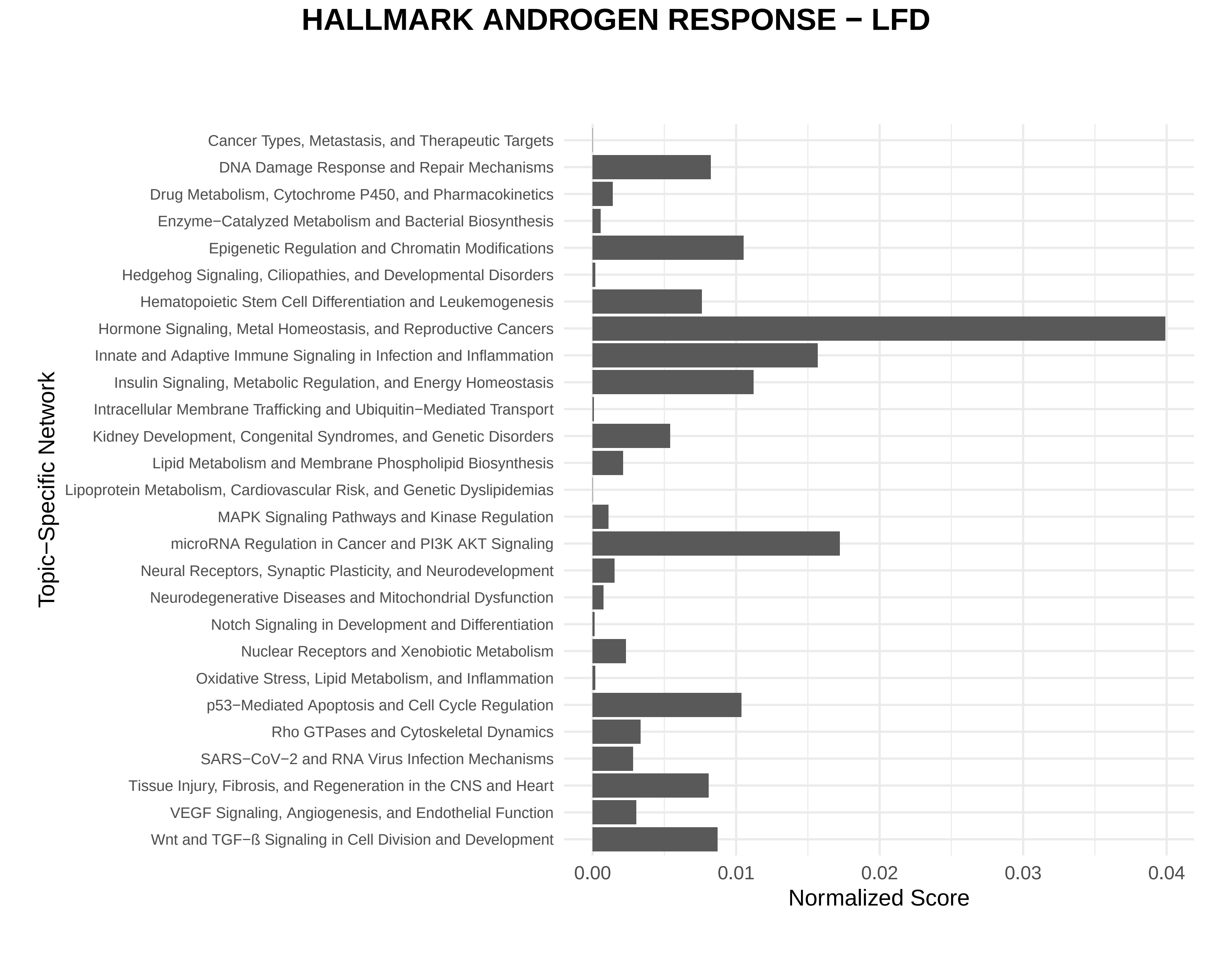
HALLMARK ANDROGEN RESPONSE − LFD
Topic−Specific Network
Cancer Types, Metastasis, and Therapeutic Targets
DNA Damage Response and Repair Mechanisms
Drug Metabolism, Cytochrome P450, and Pharmacokinetics
Enzyme−Catalyzed Metabolism and Bacterial Biosynthesis
Epigenetic Regulation and Chromatin Modifications
Hedgehog Signaling, Ciliopathies, and Developmental Disorders
Hematopoietic Stem Cell Differentiation and Leukemogenesis
Hormone Signaling, Metal Homeostasis, and Reproductive Cancers
Innate and Adaptive Immune Signaling in Infection and Inflammation
Insulin Signaling, Metabolic Regulation, and Energy Homeostasis
Intracellular Membrane Trafficking and Ubiquitin−Mediated Transport
Kidney Development, Congenital Syndromes, and Genetic Disorders
Lipid Metabolism and Membrane Phospholipid Biosynthesis
Lipoprotein Metabolism, Cardiovascular Risk, and Genetic Dyslipidemias
MAPK Signaling Pathways and Kinase Regulation
microRNA Regulation in Cancer and PI3K AKT Signaling
Neural Receptors, Synaptic Plasticity, and Neurodevelopment
Neurodegenerative Diseases and Mitochondrial Dysfunction
Notch Signaling in Development and Differentiation
Nuclear Receptors and Xenobiotic Metabolism
Oxidative Stress, Lipid Metabolism, and Inflammation
p53−Mediated Apoptosis and Cell Cycle Regulation
Rho GTPases and Cytoskeletal Dynamics
SARS−CoV−2 and RNA Virus Infection Mechanisms
Tissue Injury, Fibrosis, and Regeneration in the CNS and Heart
VEGF Signaling, Angiogenesis, and Endothelial Function
Wnt and TGF−ß Signaling in Cell Division and Development
0.00
0.01
0.02
0.03
0.04
Normalized Score

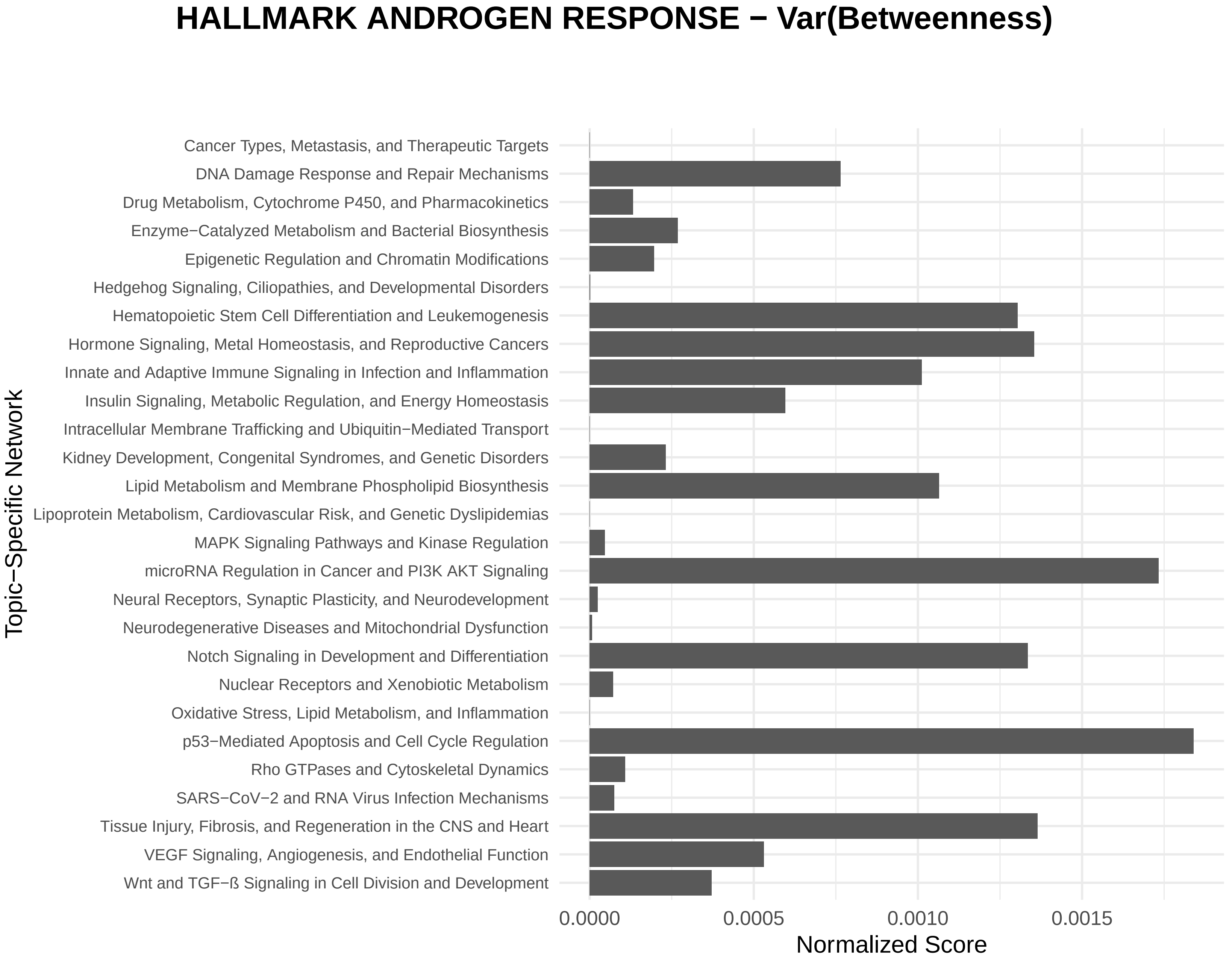
HALLMARK ANDROGEN RESPONSE − Var(Betweenness)
Topic−Specific Network
Normalized Score
Cancer Types, Metastasis, and Therapeutic Targets
DNA Damage Response and Repair Mechanisms
Drug Metabolism, Cytochrome P450, and Pharmacokinetics
Enzyme−Catalyzed Metabolism and Bacterial Biosynthesis
Epigenetic Regulation and Chromatin Modifications
Hedgehog Signaling, Ciliopathies, and Developmental Disorders
Hematopoietic Stem Cell Differentiation and Leukemogenesis
Hormone Signaling, Metal Homeostasis, and Reproductive Cancers
Innate and Adaptive Immune Signaling in Infection and Inflammation
Insulin Signaling, Metabolic Regulation, and Energy Homeostasis
Intracellular Membrane Trafficking and Ubiquitin−Mediated Transport
Kidney Development, Congenital Syndromes, and Genetic Disorders
Lipid Metabolism and Membrane Phospholipid Biosynthesis
Lipoprotein Metabolism, Cardiovascular Risk, and Genetic Dyslipidemias
MAPK Signaling Pathways and Kinase Regulation
microRNA Regulation in Cancer and PI3K AKT Signaling
Neural Receptors, Synaptic Plasticity, and Neurodevelopment
Neurodegenerative Diseases and Mitochondrial Dysfunction
Notch Signaling in Development and Differentiation
Nuclear Receptors and Xenobiotic Metabolism
Oxidative Stress, Lipid Metabolism, and Inflammation
p53−Mediated Apoptosis and Cell Cycle Regulation
Rho GTPases and Cytoskeletal Dynamics
SARS−CoV−2 and RNA Virus Infection Mechanisms
Tissue Injury, Fibrosis, and Regeneration in the CNS and Heart
VEGF Signaling, Angiogenesis, and Endothelial Function
Wnt and TGF−ß Signaling in Cell Division and Development
0.0000
0.0005
0.0010
0.0015

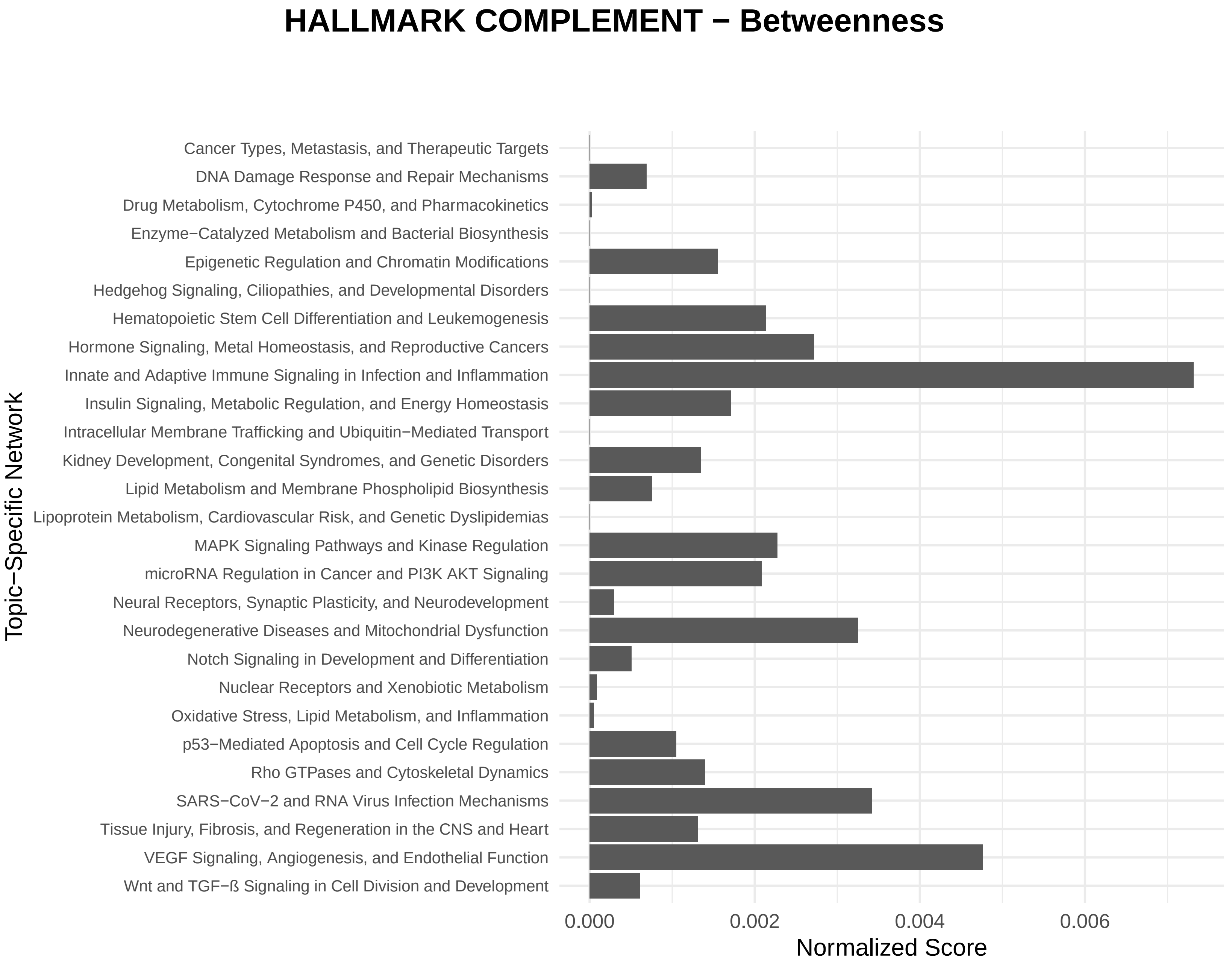
HALLMARK COMPLEMENT − Betweenness
Topic−Specific Network
Cancer Types, Metastasis, and Therapeutic Targets
DNA Damage Response and Repair Mechanisms
Drug Metabolism, Cytochrome P450, and Pharmacokinetics
Enzyme−Catalyzed Metabolism and Bacterial Biosynthesis
Epigenetic Regulation and Chromatin Modifications
Hedgehog Signaling, Ciliopathies, and Developmental Disorders
Hematopoietic Stem Cell Differentiation and Leukemogenesis
Hormone Signaling, Metal Homeostasis, and Reproductive Cancers
Innate and Adaptive Immune Signaling in Infection and Inflammation
Insulin Signaling, Metabolic Regulation, and Energy Homeostasis
Intracellular Membrane Trafficking and Ubiquitin−Mediated Transport
Kidney Development, Congenital Syndromes, and Genetic Disorders
Lipid Metabolism and Membrane Phospholipid Biosynthesis
Lipoprotein Metabolism, Cardiovascular Risk, and Genetic Dyslipidemias
MAPK Signaling Pathways and Kinase Regulation
microRNA Regulation in Cancer and PI3K AKT Signaling
Neural Receptors, Synaptic Plasticity, and Neurodevelopment
Neurodegenerative Diseases and Mitochondrial Dysfunction
Notch Signaling in Development and Differentiation
Nuclear Receptors and Xenobiotic Metabolism
Oxidative Stress, Lipid Metabolism, and Inflammation
p53−Mediated Apoptosis and Cell Cycle Regulation
Rho GTPases and Cytoskeletal Dynamics
SARS−CoV−2 and RNA Virus Infection Mechanisms
Tissue Injury, Fibrosis, and Regeneration in the CNS and Heart
VEGF Signaling, Angiogenesis, and Endothelial Function
Wnt and TGF−ß Signaling in Cell Division and Development
0.000
0.002
0.004
0.006
Normalized Score

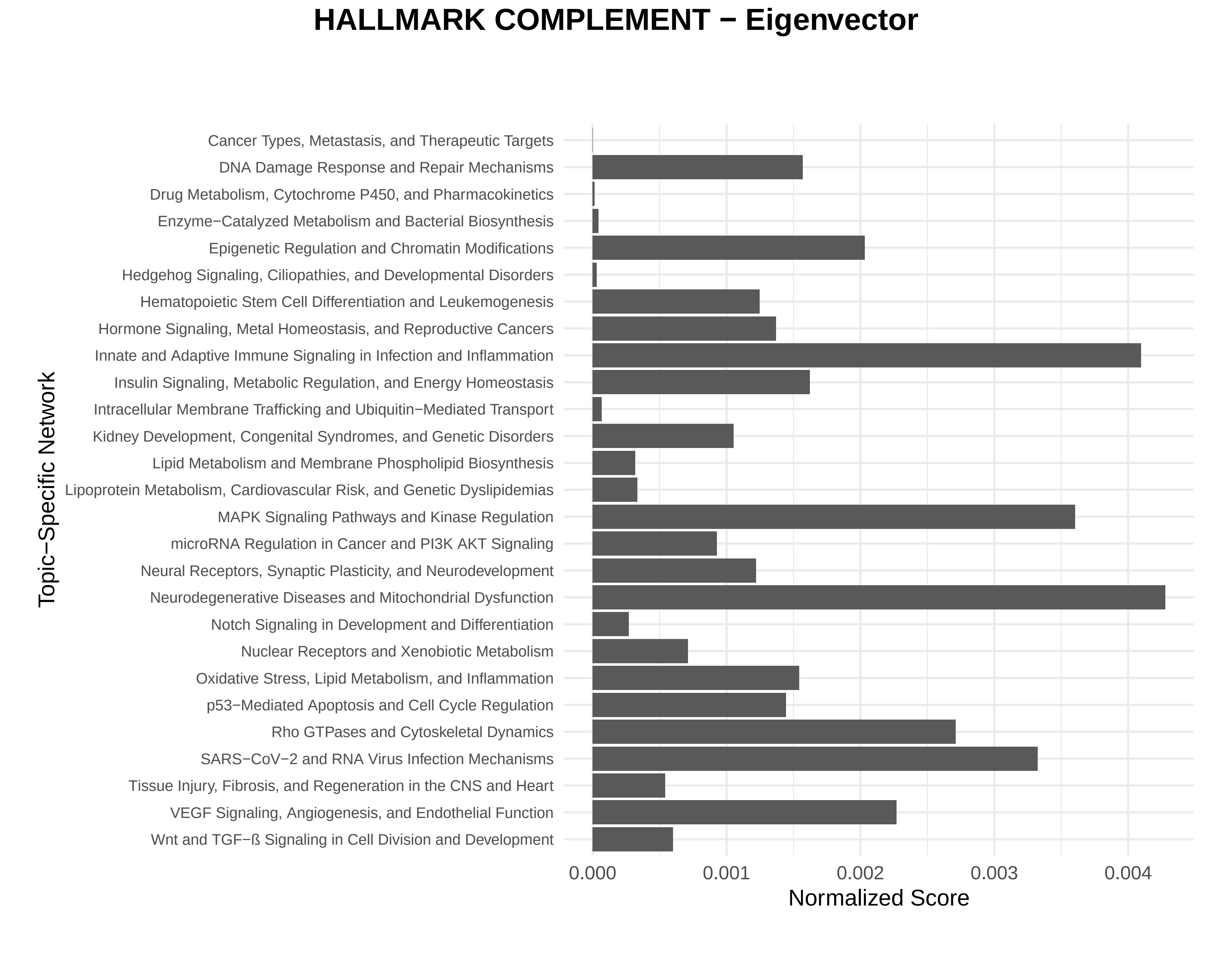

HALLMARK COMPLEMENT − Eigenvector
Topic−Specific Network
Cancer Types, Metastasis, and Therapeutic Targets
DNA Damage Response and Repair Mechanisms
Drug Metabolism, Cytochrome P450, and Pharmacokinetics
Enzyme−Catalyzed Metabolism and Bacterial Biosynthesis
Epigenetic Regulation and Chromatin Modifications
Hedgehog Signaling, Ciliopathies, and Developmental Disorders
Hematopoietic Stem Cell Differentiation and Leukemogenesis
Hormone Signaling, Metal Homeostasis, and Reproductive Cancers
Innate and Adaptive Immune Signaling in Infection and Inflammation
Insulin Signaling, Metabolic Regulation, and Energy Homeostasis
Intracellular Membrane Trafficking and Ubiquitin−Mediated Transport
Kidney Development, Congenital Syndromes, and Genetic Disorders
Lipid Metabolism and Membrane Phospholipid Biosynthesis
Lipoprotein Metabolism, Cardiovascular Risk, and Genetic Dyslipidemias
MAPK Signaling Pathways and Kinase Regulation
microRNA Regulation in Cancer and PI3K AKT Signaling
Neural Receptors, Synaptic Plasticity, and Neurodevelopment
Neurodegenerative Diseases and Mitochondrial Dysfunction
Notch Signaling in Development and Differentiation
Nuclear Receptors and Xenobiotic Metabolism
Oxidative Stress, Lipid Metabolism, and Inflammation
p53−Mediated Apoptosis and Cell Cycle Regulation
Rho GTPases and Cytoskeletal Dynamics
SARS−CoV−2 and RNA Virus Infection Mechanisms
Tissue Injury, Fibrosis, and Regeneration in the CNS and Heart
VEGF Signaling, Angiogenesis, and Endothelial Function
Wnt and TGF−ß Signaling in Cell Division and Development
0.000
0.001
0.002
0.003
0.004
Normalized Score

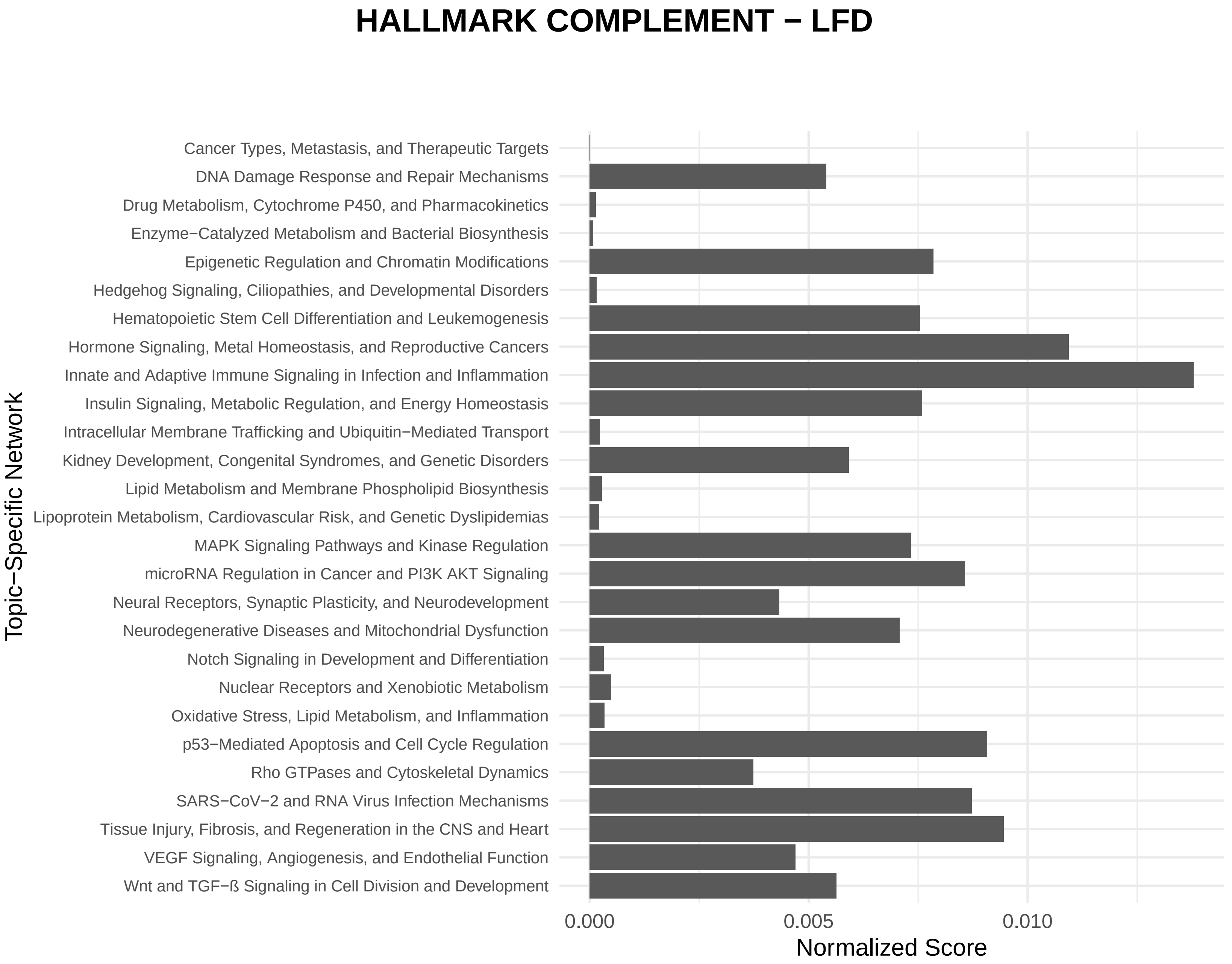
HALLMARK COMPLEMENT − LFD
Topic−Specific Network
Cancer Types, Metastasis, and Therapeutic Targets
DNA Damage Response and Repair Mechanisms
Drug Metabolism, Cytochrome P450, and Pharmacokinetics
Enzyme−Catalyzed Metabolism and Bacterial Biosynthesis
Epigenetic Regulation and Chromatin Modifications
Hedgehog Signaling, Ciliopathies, and Developmental Disorders
Hematopoietic Stem Cell Differentiation and Leukemogenesis
Hormone Signaling, Metal Homeostasis, and Reproductive Cancers
Innate and Adaptive Immune Signaling in Infection and Inflammation
Insulin Signaling, Metabolic Regulation, and Energy Homeostasis
Intracellular Membrane Trafficking and Ubiquitin−Mediated Transport
Kidney Development, Congenital Syndromes, and Genetic Disorders
Lipid Metabolism and Membrane Phospholipid Biosynthesis
Lipoprotein Metabolism, Cardiovascular Risk, and Genetic Dyslipidemias
MAPK Signaling Pathways and Kinase Regulation
microRNA Regulation in Cancer and PI3K AKT Signaling
Neural Receptors, Synaptic Plasticity, and Neurodevelopment
Neurodegenerative Diseases and Mitochondrial Dysfunction
Notch Signaling in Development and Differentiation
Nuclear Receptors and Xenobiotic Metabolism
Oxidative Stress, Lipid Metabolism, and Inflammation
p53−Mediated Apoptosis and Cell Cycle Regulation
Rho GTPases and Cytoskeletal Dynamics
SARS−CoV−2 and RNA Virus Infection Mechanisms
Tissue Injury, Fibrosis, and Regeneration in the CNS and Heart
VEGF Signaling, Angiogenesis, and Endothelial Function
Wnt and TGF−ß Signaling in Cell Division and Development
0.000
0.005
0.010
Normalized Score

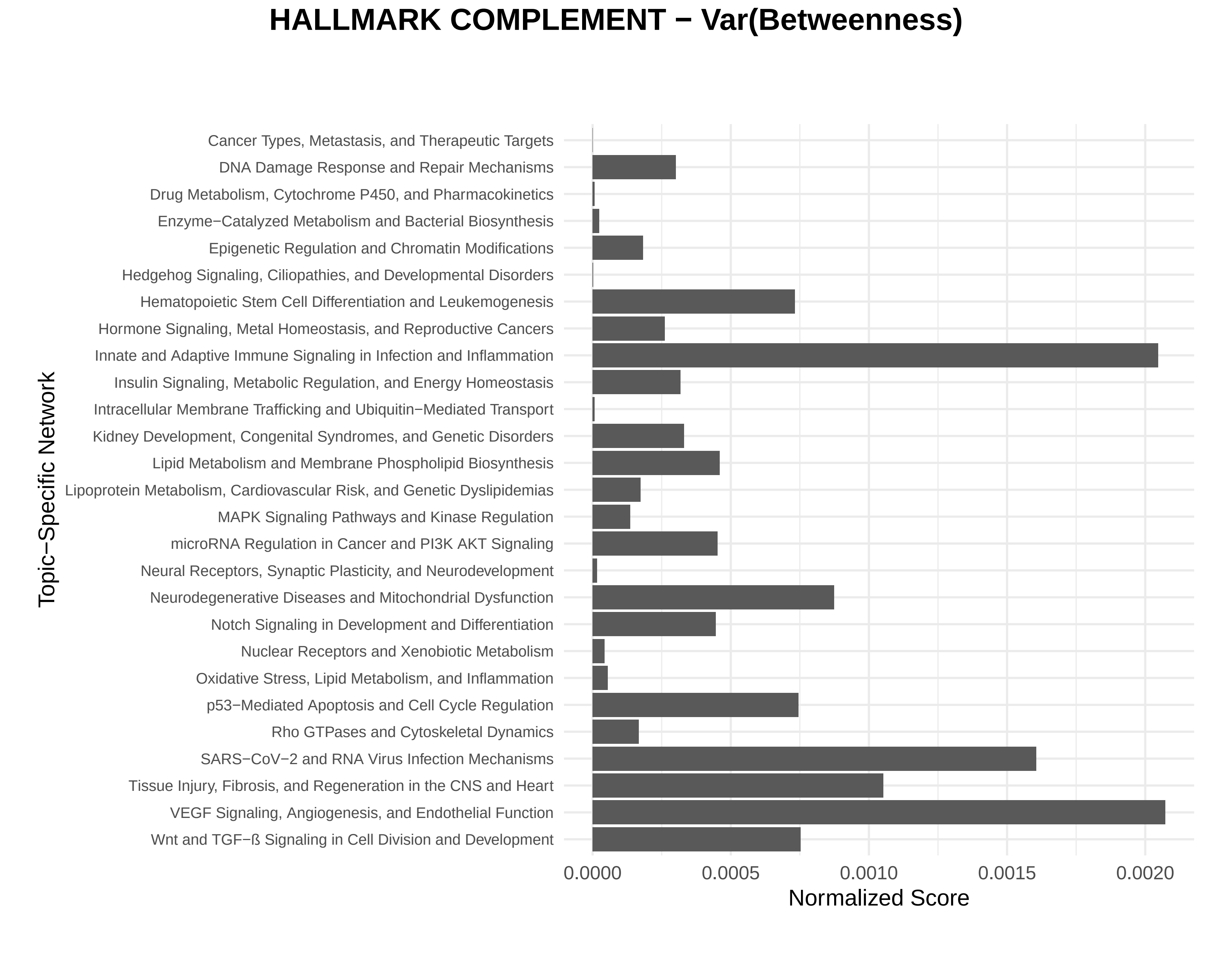

HALLMARK COMPLEMENT − Var(Betweenness)
Topic−Specific Network
Cancer Types, Metastasis, and Therapeutic Targets
DNA Damage Response and Repair Mechanisms
Drug Metabolism, Cytochrome P450, and Pharmacokinetics
Enzyme−Catalyzed Metabolism and Bacterial Biosynthesis
Epigenetic Regulation and Chromatin Modifications
Hedgehog Signaling, Ciliopathies, and Developmental Disorders
Hematopoietic Stem Cell Differentiation and Leukemogenesis
Hormone Signaling, Metal Homeostasis, and Reproductive Cancers
Innate and Adaptive Immune Signaling in Infection and Inflammation
Insulin Signaling, Metabolic Regulation, and Energy Homeostasis
Intracellular Membrane Trafficking and Ubiquitin−Mediated Transport
Kidney Development, Congenital Syndromes, and Genetic Disorders
Lipid Metabolism and Membrane Phospholipid Biosynthesis
Lipoprotein Metabolism, Cardiovascular Risk, and Genetic Dyslipidemias
MAPK Signaling Pathways and Kinase Regulation
microRNA Regulation in Cancer and PI3K AKT Signaling
Neural Receptors, Synaptic Plasticity, and Neurodevelopment
Neurodegenerative Diseases and Mitochondrial Dysfunction
Notch Signaling in Development and Differentiation
Nuclear Receptors and Xenobiotic Metabolism
Oxidative Stress, Lipid Metabolism, and Inflammation
p53−Mediated Apoptosis and Cell Cycle Regulation
Rho GTPases and Cytoskeletal Dynamics
SARS−CoV−2 and RNA Virus Infection Mechanisms
Tissue Injury, Fibrosis, and Regeneration in the CNS and Heart
VEGF Signaling, Angiogenesis, and Endothelial Function
Wnt and TGF−ß Signaling in Cell Division and Development
0.0000
0.0005
0.0010
0.0015
0.0020
Normalized Score

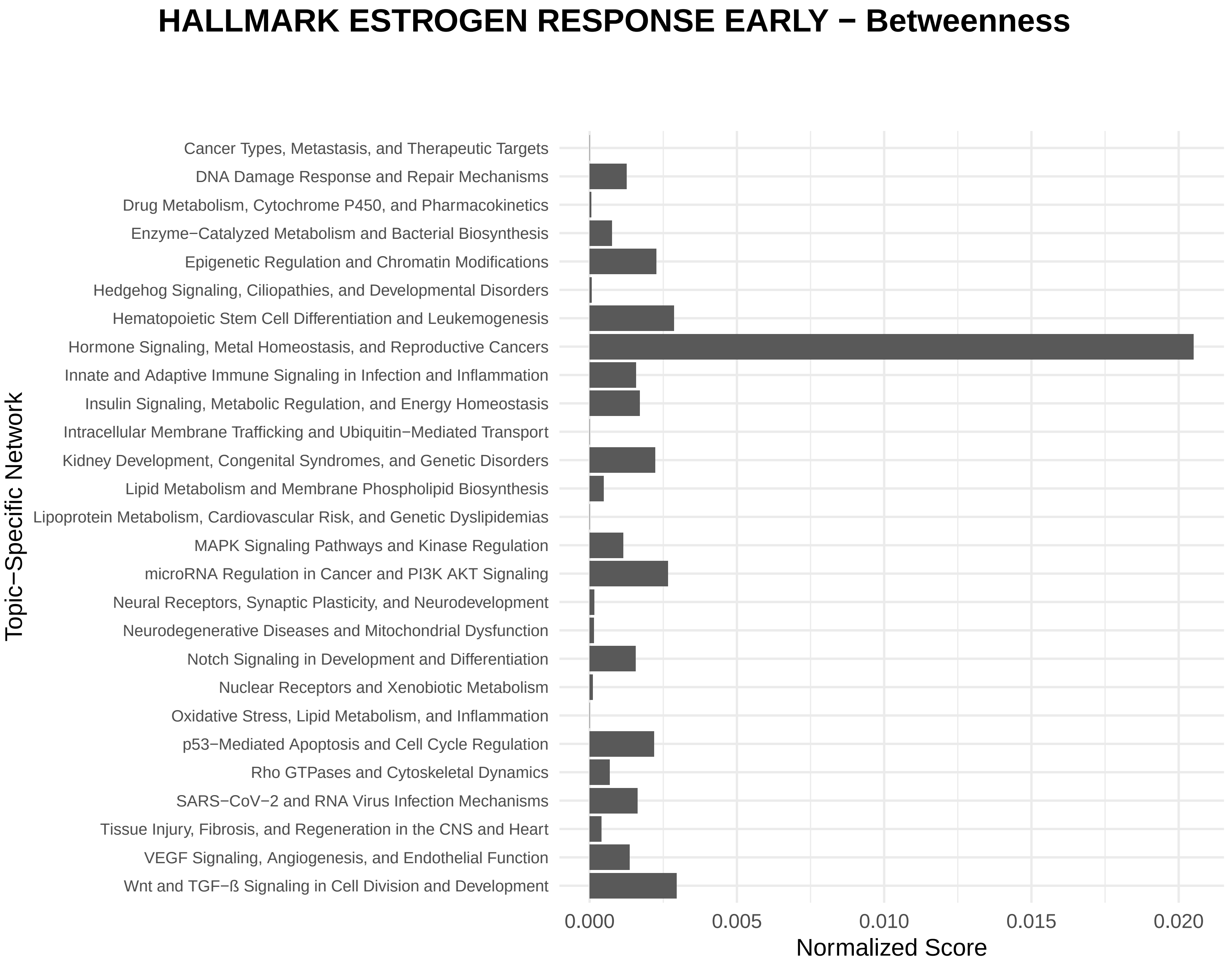
HALLMARK ESTROGEN RESPONSE EARLY − Betweenness
Topic−Specific Network
Normalized Score
Cancer Types, Metastasis, and Therapeutic Targets
DNA Damage Response and Repair Mechanisms
Drug Metabolism, Cytochrome P450, and Pharmacokinetics
Enzyme−Catalyzed Metabolism and Bacterial Biosynthesis
Epigenetic Regulation and Chromatin Modifications
Hedgehog Signaling, Ciliopathies, and Developmental Disorders
Hematopoietic Stem Cell Differentiation and Leukemogenesis
Hormone Signaling, Metal Homeostasis, and Reproductive Cancers
Innate and Adaptive Immune Signaling in Infection and Inflammation
Insulin Signaling, Metabolic Regulation, and Energy Homeostasis
Intracellular Membrane Trafficking and Ubiquitin−Mediated Transport
Kidney Development, Congenital Syndromes, and Genetic Disorders
Lipid Metabolism and Membrane Phospholipid Biosynthesis
Lipoprotein Metabolism, Cardiovascular Risk, and Genetic Dyslipidemias
MAPK Signaling Pathways and Kinase Regulation
microRNA Regulation in Cancer and PI3K AKT Signaling
Neural Receptors, Synaptic Plasticity, and Neurodevelopment
Neurodegenerative Diseases and Mitochondrial Dysfunction
Notch Signaling in Development and Differentiation
Nuclear Receptors and Xenobiotic Metabolism
Oxidative Stress, Lipid Metabolism, and Inflammation
p53−Mediated Apoptosis and Cell Cycle Regulation
Rho GTPases and Cytoskeletal Dynamics
SARS−CoV−2 and RNA Virus Infection Mechanisms
Tissue Injury, Fibrosis, and Regeneration in the CNS and Heart
VEGF Signaling, Angiogenesis, and Endothelial Function
Wnt and TGF−ß Signaling in Cell Division and Development
0.000
0.005
0.010
0.015
0.020

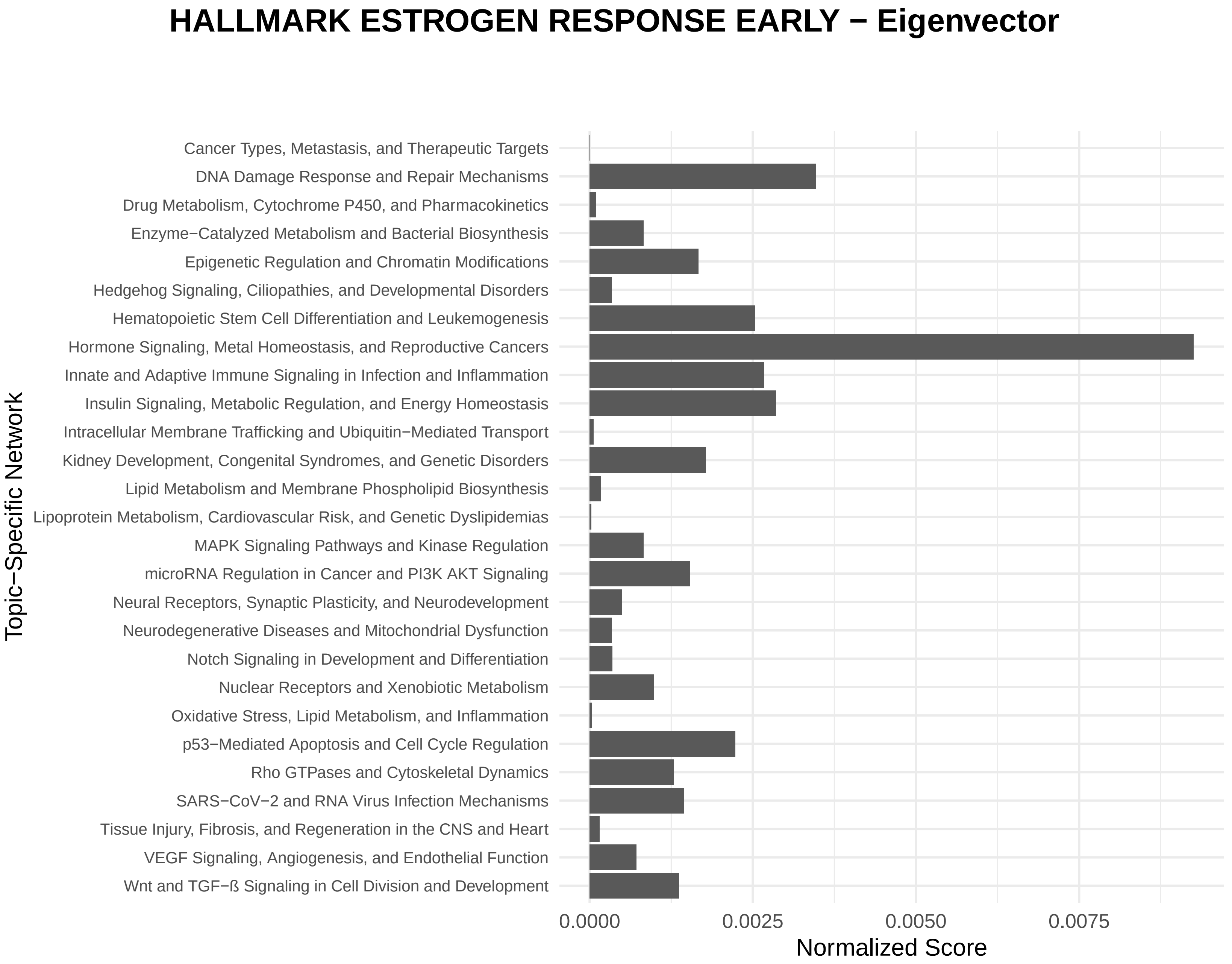
HALLMARK ESTROGEN RESPONSE EARLY − Eigenvector
Cancer Types, Metastasis, and Therapeutic Targets
DNA Damage Response and Repair Mechanisms
Drug Metabolism, Cytochrome P450, and Pharmacokinetics
Enzyme−Catalyzed Metabolism and Bacterial Biosynthesis
Epigenetic Regulation and Chromatin Modifications
Hedgehog Signaling, Ciliopathies, and Developmental Disorders
Hematopoietic Stem Cell Differentiation and Leukemogenesis
Hormone Signaling, Metal Homeostasis, and Reproductive Cancers
Innate and Adaptive Immune Signaling in Infection and Inflammation
Insulin Signaling, Metabolic Regulation, and Energy Homeostasis
Intracellular Membrane Trafficking and Ubiquitin−Mediated Transport
Kidney Development, Congenital Syndromes, and Genetic Disorders
Lipid Metabolism and Membrane Phospholipid Biosynthesis
Lipoprotein Metabolism, Cardiovascular Risk, and Genetic Dyslipidemias
MAPK Signaling Pathways and Kinase Regulation
microRNA Regulation in Cancer and PI3K AKT Signaling
Neural Receptors, Synaptic Plasticity, and Neurodevelopment
Neurodegenerative Diseases and Mitochondrial Dysfunction
Notch Signaling in Development and Differentiation
Nuclear Receptors and Xenobiotic Metabolism
Oxidative Stress, Lipid Metabolism, and Inflammation
p53−Mediated Apoptosis and Cell Cycle Regulation
Rho GTPases and Cytoskeletal Dynamics
SARS−CoV−2 and RNA Virus Infection Mechanisms
Tissue Injury, Fibrosis, and Regeneration in the CNS and Heart
VEGF Signaling, Angiogenesis, and Endothelial Function
Wnt and TGF−ß Signaling in Cell Division and Development
Topic−Specific Network
0.0000
0.0025
0.0050
0.0075
Normalized Score

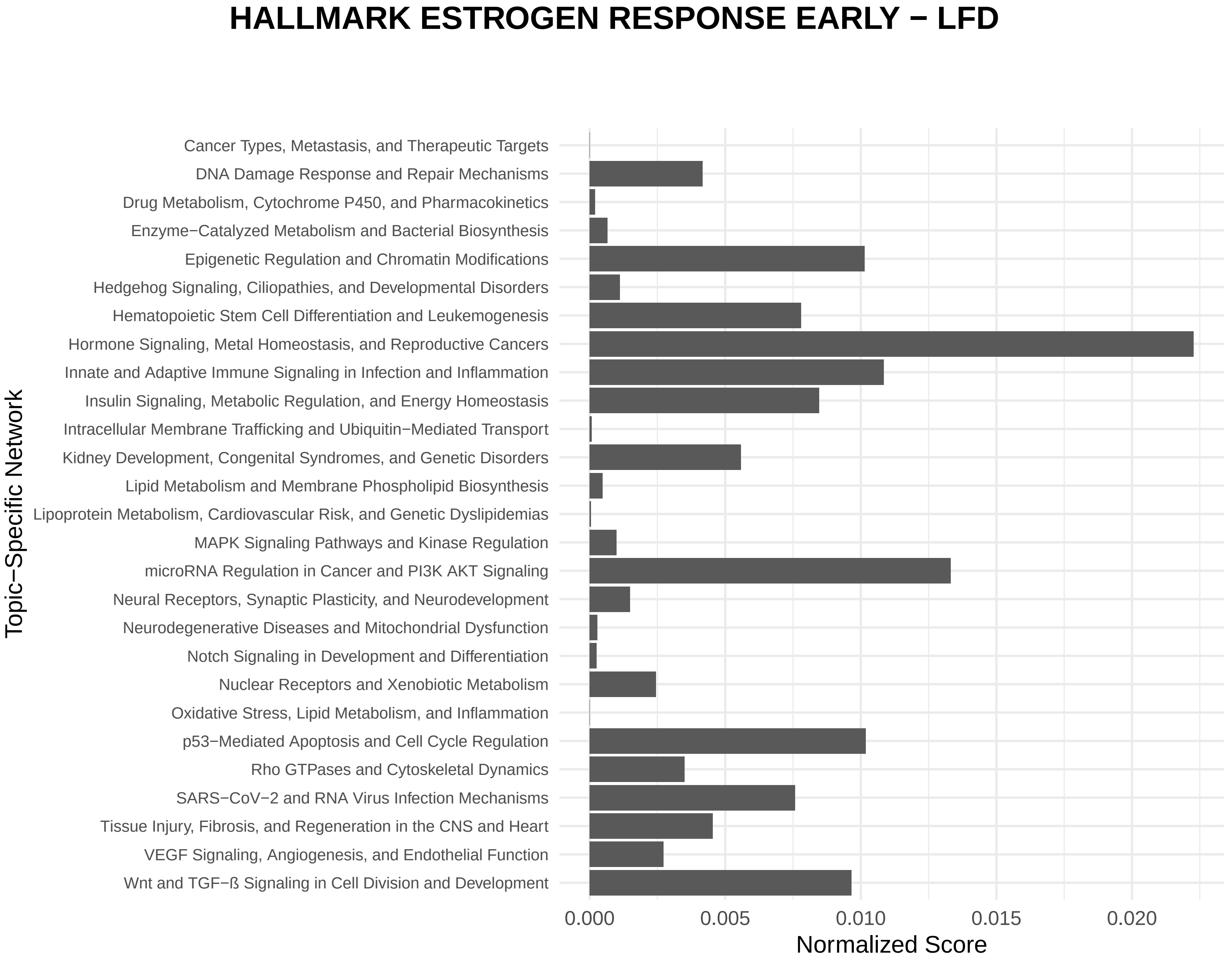
HALLMARK ESTROGEN RESPONSE EARLY − LFD
Topic−Specific Network
Cancer Types, Metastasis, and Therapeutic Targets
DNA Damage Response and Repair Mechanisms
Drug Metabolism, Cytochrome P450, and Pharmacokinetics
Enzyme−Catalyzed Metabolism and Bacterial Biosynthesis
Epigenetic Regulation and Chromatin Modifications
Hedgehog Signaling, Ciliopathies, and Developmental Disorders
Hematopoietic Stem Cell Differentiation and Leukemogenesis
Hormone Signaling, Metal Homeostasis, and Reproductive Cancers
Innate and Adaptive Immune Signaling in Infection and Inflammation
Insulin Signaling, Metabolic Regulation, and Energy Homeostasis
Intracellular Membrane Trafficking and Ubiquitin−Mediated Transport
Kidney Development, Congenital Syndromes, and Genetic Disorders
Lipid Metabolism and Membrane Phospholipid Biosynthesis
Lipoprotein Metabolism, Cardiovascular Risk, and Genetic Dyslipidemias
MAPK Signaling Pathways and Kinase Regulation
microRNA Regulation in Cancer and PI3K AKT Signaling
Neural Receptors, Synaptic Plasticity, and Neurodevelopment
Neurodegenerative Diseases and Mitochondrial Dysfunction
Notch Signaling in Development and Differentiation
Nuclear Receptors and Xenobiotic Metabolism
Oxidative Stress, Lipid Metabolism, and Inflammation
p53−Mediated Apoptosis and Cell Cycle Regulation
Rho GTPases and Cytoskeletal Dynamics
SARS−CoV−2 and RNA Virus Infection Mechanisms
Tissue Injury, Fibrosis, and Regeneration in the CNS and Heart
VEGF Signaling, Angiogenesis, and Endothelial Function
Wnt and TGF−ß Signaling in Cell Division and Development
0.000
0.005
0.010
0.015
0.020
Normalized Score

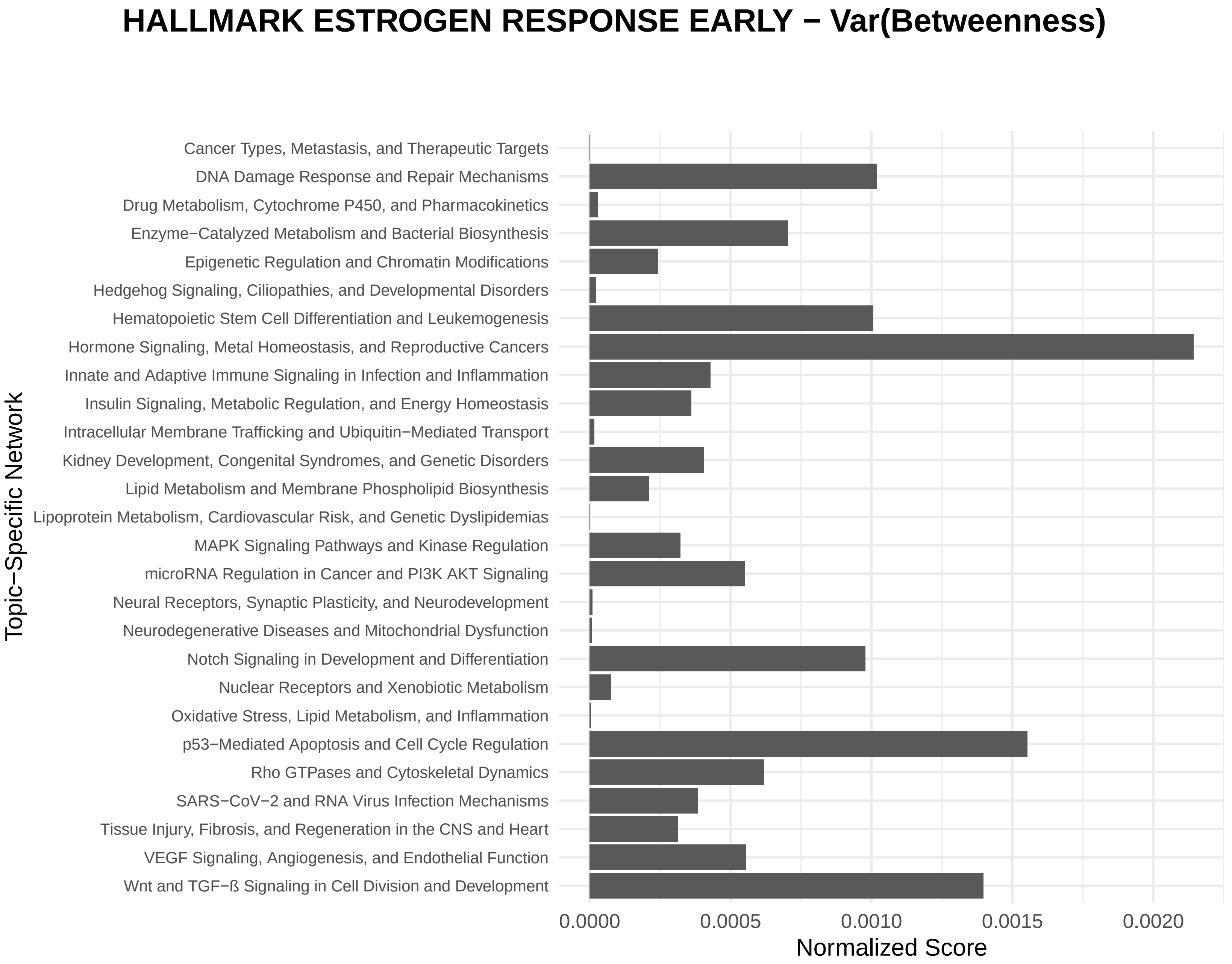

HALLMARK ESTROGEN RESPONSE EARLY − Var(Betweenness)
Topic−Specific Network
Cancer Types, Metastasis, and Therapeutic Targets
DNA Damage Response and Repair Mechanisms
Drug Metabolism, Cytochrome P450, and Pharmacokinetics
Enzyme−Catalyzed Metabolism and Bacterial Biosynthesis
Epigenetic Regulation and Chromatin Modifications
Hedgehog Signaling, Ciliopathies, and Developmental Disorders
Hematopoietic Stem Cell Differentiation and Leukemogenesis
Hormone Signaling, Metal Homeostasis, and Reproductive Cancers
Innate and Adaptive Immune Signaling in Infection and Inflammation
Insulin Signaling, Metabolic Regulation, and Energy Homeostasis
Intracellular Membrane Trafficking and Ubiquitin−Mediated Transport
Kidney Development, Congenital Syndromes, and Genetic Disorders
Lipid Metabolism and Membrane Phospholipid Biosynthesis
Lipoprotein Metabolism, Cardiovascular Risk, and Genetic Dyslipidemias
MAPK Signaling Pathways and Kinase Regulation
microRNA Regulation in Cancer and PI3K AKT Signaling
Neural Receptors, Synaptic Plasticity, and Neurodevelopment
Neurodegenerative Diseases and Mitochondrial Dysfunction
Notch Signaling in Development and Differentiation
Nuclear Receptors and Xenobiotic Metabolism
Oxidative Stress, Lipid Metabolism, and Inflammation
p53−Mediated Apoptosis and Cell Cycle Regulation
Rho GTPases and Cytoskeletal Dynamics
SARS−CoV−2 and RNA Virus Infection Mechanisms
Tissue Injury, Fibrosis, and Regeneration in the CNS and Heart
VEGF Signaling, Angiogenesis, and Endothelial Function
Wnt and TGF−ß Signaling in Cell Division and Development
0.0000
0.0005
0.0010
0.0015
0.0020
Normalized Score

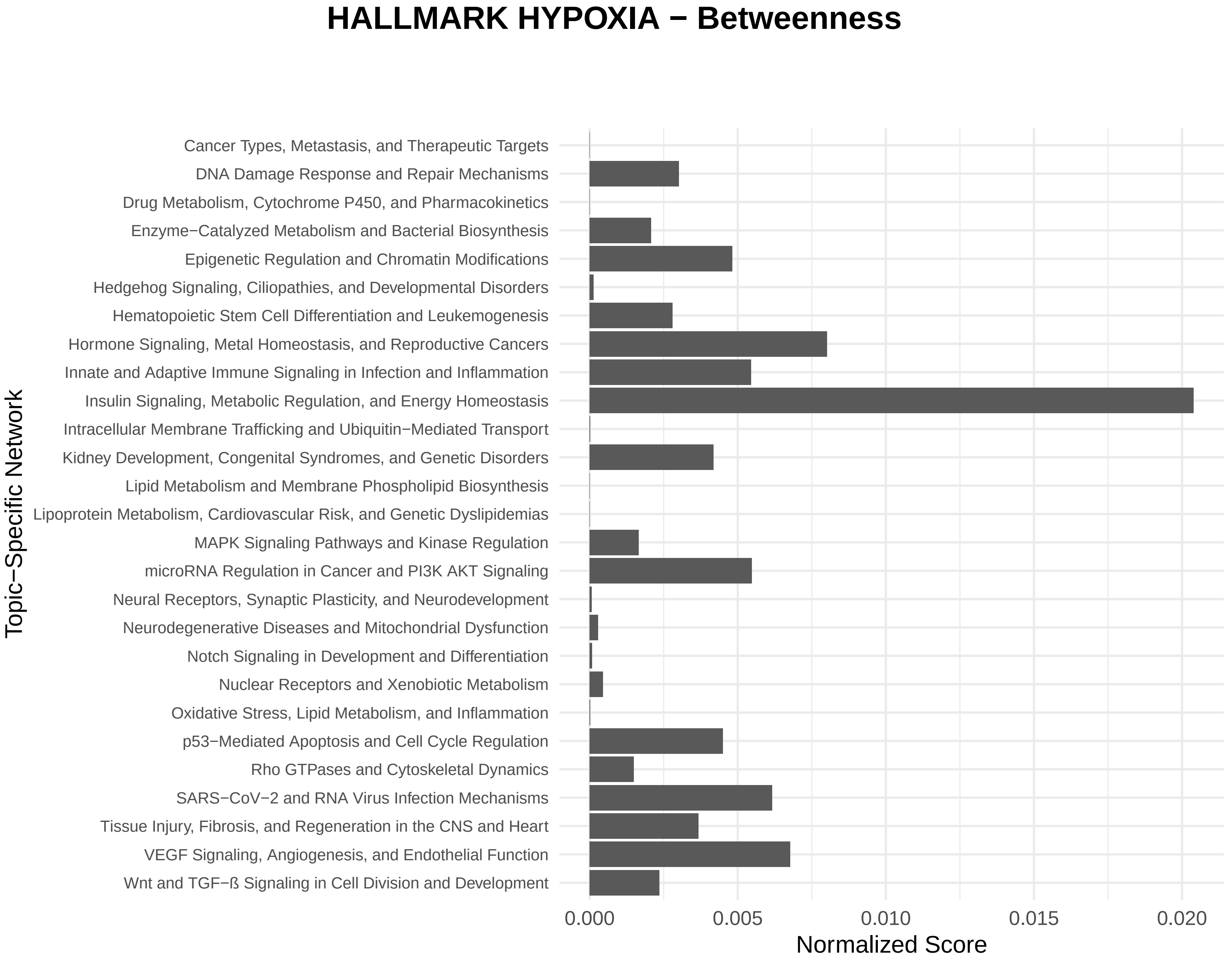
HALLMARK HYPOXIA − Betweenness
Topic−Specific Network
Cancer Types, Metastasis, and Therapeutic Targets
DNA Damage Response and Repair Mechanisms
Drug Metabolism, Cytochrome P450, and Pharmacokinetics
Enzyme−Catalyzed Metabolism and Bacterial Biosynthesis
Epigenetic Regulation and Chromatin Modifications
Hedgehog Signaling, Ciliopathies, and Developmental Disorders
Hematopoietic Stem Cell Differentiation and Leukemogenesis
Hormone Signaling, Metal Homeostasis, and Reproductive Cancers
Innate and Adaptive Immune Signaling in Infection and Inflammation
Insulin Signaling, Metabolic Regulation, and Energy Homeostasis
Intracellular Membrane Trafficking and Ubiquitin−Mediated Transport
Kidney Development, Congenital Syndromes, and Genetic Disorders
Lipid Metabolism and Membrane Phospholipid Biosynthesis
Lipoprotein Metabolism, Cardiovascular Risk, and Genetic Dyslipidemias
MAPK Signaling Pathways and Kinase Regulation
microRNA Regulation in Cancer and PI3K AKT Signaling
Neural Receptors, Synaptic Plasticity, and Neurodevelopment
Neurodegenerative Diseases and Mitochondrial Dysfunction
Notch Signaling in Development and Differentiation
Nuclear Receptors and Xenobiotic Metabolism
Oxidative Stress, Lipid Metabolism, and Inflammation
p53−Mediated Apoptosis and Cell Cycle Regulation
Rho GTPases and Cytoskeletal Dynamics
SARS−CoV−2 and RNA Virus Infection Mechanisms
Tissue Injury, Fibrosis, and Regeneration in the CNS and Heart
VEGF Signaling, Angiogenesis, and Endothelial Function
Wnt and TGF−ß Signaling in Cell Division and Development
0.000
0.005
0.010
0.015
0.020
Normalized Score

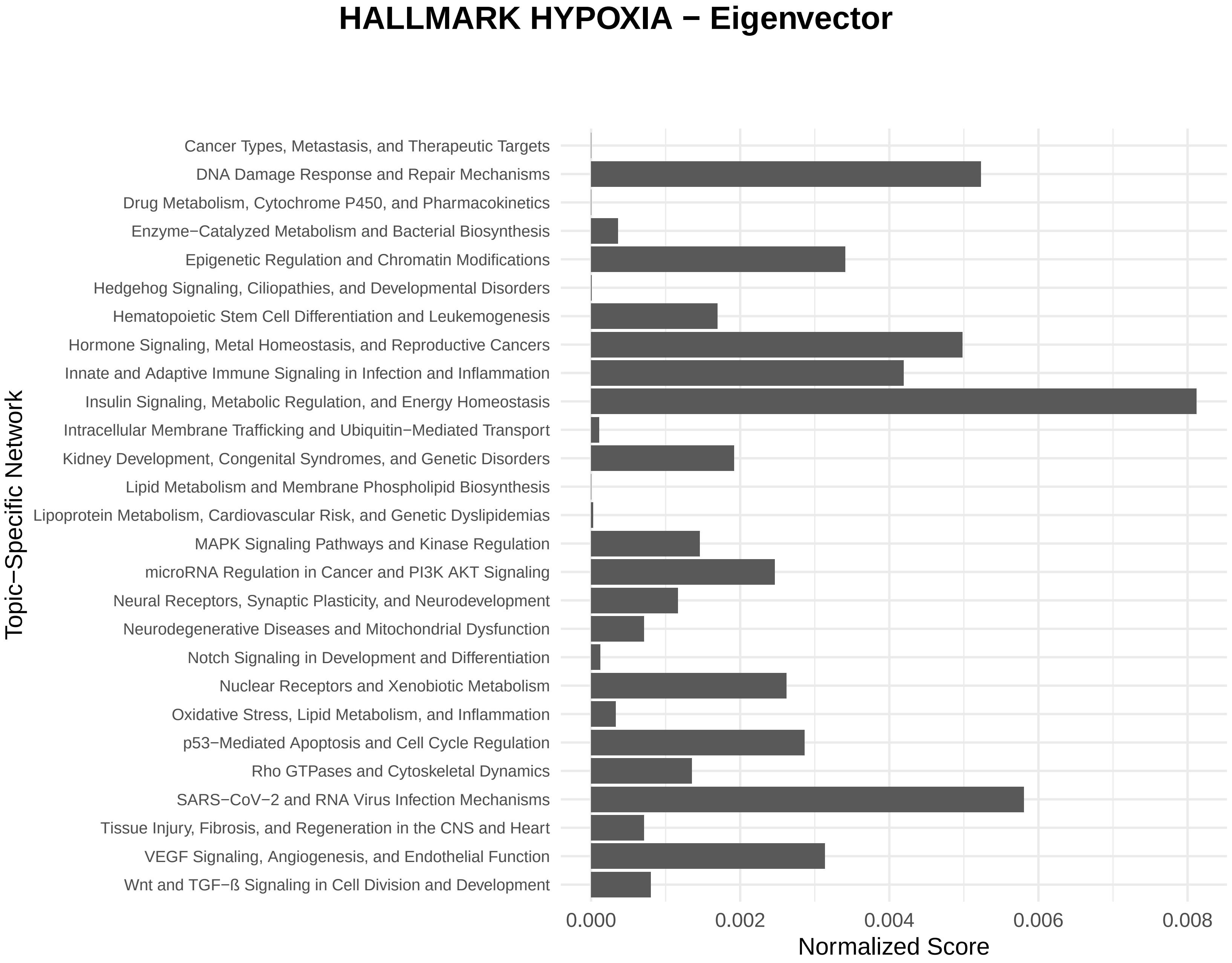
HALLMARK HYPOXIA − Eigenvector
Topic−Specific Network
Cancer Types, Metastasis, and Therapeutic Targets
DNA Damage Response and Repair Mechanisms
Drug Metabolism, Cytochrome P450, and Pharmacokinetics
Enzyme−Catalyzed Metabolism and Bacterial Biosynthesis
Epigenetic Regulation and Chromatin Modifications
Hedgehog Signaling, Ciliopathies, and Developmental Disorders
Hematopoietic Stem Cell Differentiation and Leukemogenesis
Hormone Signaling, Metal Homeostasis, and Reproductive Cancers
Innate and Adaptive Immune Signaling in Infection and Inflammation
Insulin Signaling, Metabolic Regulation, and Energy Homeostasis
Intracellular Membrane Trafficking and Ubiquitin−Mediated Transport
Kidney Development, Congenital Syndromes, and Genetic Disorders
Lipid Metabolism and Membrane Phospholipid Biosynthesis
Lipoprotein Metabolism, Cardiovascular Risk, and Genetic Dyslipidemias
MAPK Signaling Pathways and Kinase Regulation
microRNA Regulation in Cancer and PI3K AKT Signaling
Neural Receptors, Synaptic Plasticity, and Neurodevelopment
Neurodegenerative Diseases and Mitochondrial Dysfunction
Notch Signaling in Development and Differentiation
Nuclear Receptors and Xenobiotic Metabolism
Oxidative Stress, Lipid Metabolism, and Inflammation
p53−Mediated Apoptosis and Cell Cycle Regulation
Rho GTPases and Cytoskeletal Dynamics
SARS−CoV−2 and RNA Virus Infection Mechanisms
Tissue Injury, Fibrosis, and Regeneration in the CNS and Heart
VEGF Signaling, Angiogenesis, and Endothelial Function
Wnt and TGF−ß Signaling in Cell Division and Development
0.000
0.002
0.004
0.006
0.008
Normalized Score

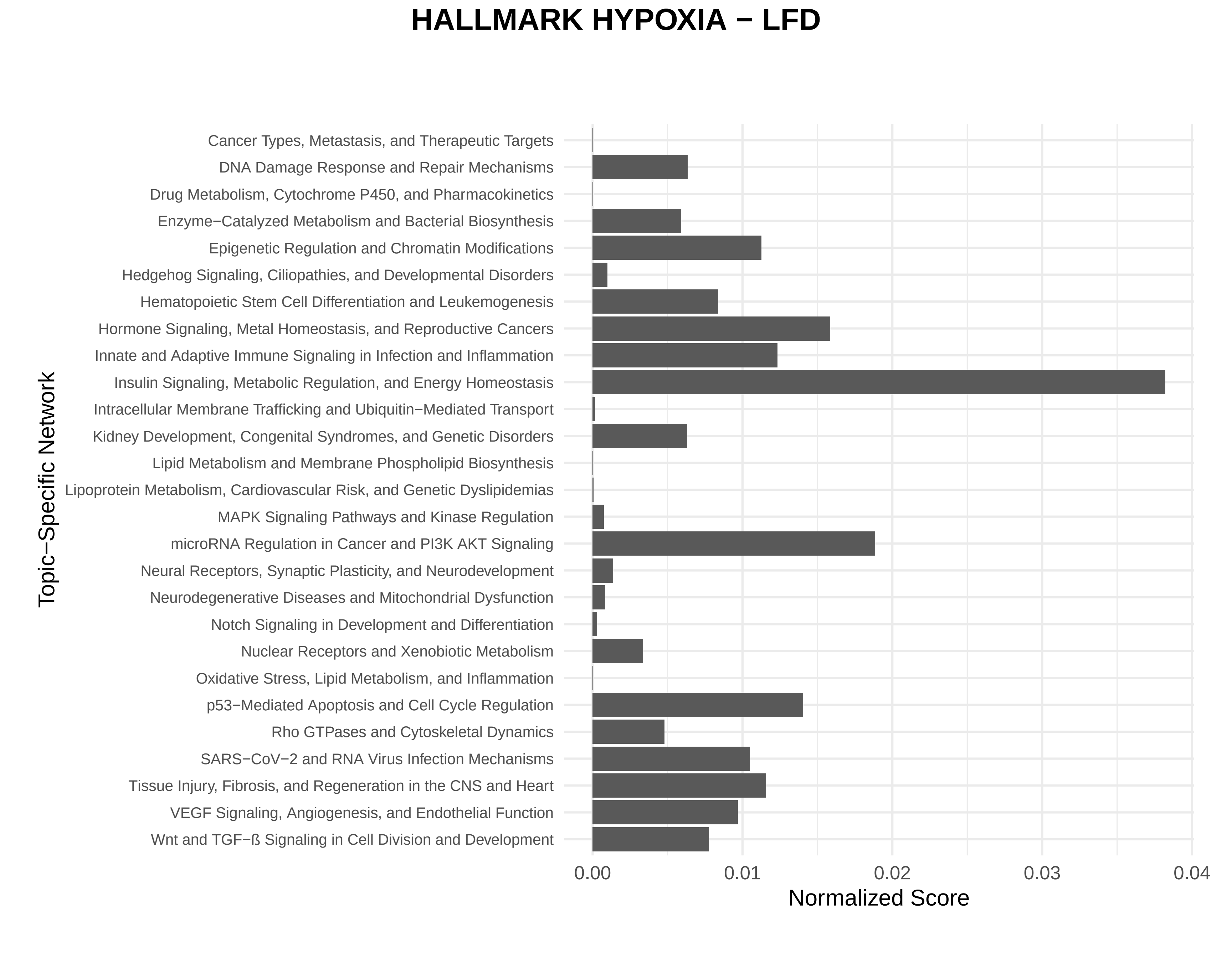

HALLMARK HYPOXIA − LFD
Topic−Specific Network
Cancer Types, Metastasis, and Therapeutic Targets
DNA Damage Response and Repair Mechanisms
Drug Metabolism, Cytochrome P450, and Pharmacokinetics
Enzyme−Catalyzed Metabolism and Bacterial Biosynthesis
Epigenetic Regulation and Chromatin Modifications
Hedgehog Signaling, Ciliopathies, and Developmental Disorders
Hematopoietic Stem Cell Differentiation and Leukemogenesis
Hormone Signaling, Metal Homeostasis, and Reproductive Cancers
Innate and Adaptive Immune Signaling in Infection and Inflammation
Insulin Signaling, Metabolic Regulation, and Energy Homeostasis
Intracellular Membrane Trafficking and Ubiquitin−Mediated Transport
Kidney Development, Congenital Syndromes, and Genetic Disorders
Lipid Metabolism and Membrane Phospholipid Biosynthesis
Lipoprotein Metabolism, Cardiovascular Risk, and Genetic Dyslipidemias
MAPK Signaling Pathways and Kinase Regulation
microRNA Regulation in Cancer and PI3K AKT Signaling
Neural Receptors, Synaptic Plasticity, and Neurodevelopment
Neurodegenerative Diseases and Mitochondrial Dysfunction
Notch Signaling in Development and Differentiation
Nuclear Receptors and Xenobiotic Metabolism
Oxidative Stress, Lipid Metabolism, and Inflammation
p53−Mediated Apoptosis and Cell Cycle Regulation
Rho GTPases and Cytoskeletal Dynamics
SARS−CoV−2 and RNA Virus Infection Mechanisms
Tissue Injury, Fibrosis, and Regeneration in the CNS and Heart
VEGF Signaling, Angiogenesis, and Endothelial Function
Wnt and TGF−ß Signaling in Cell Division and Development
0.00
0.01
0.02
0.03
0.04
Normalized Score

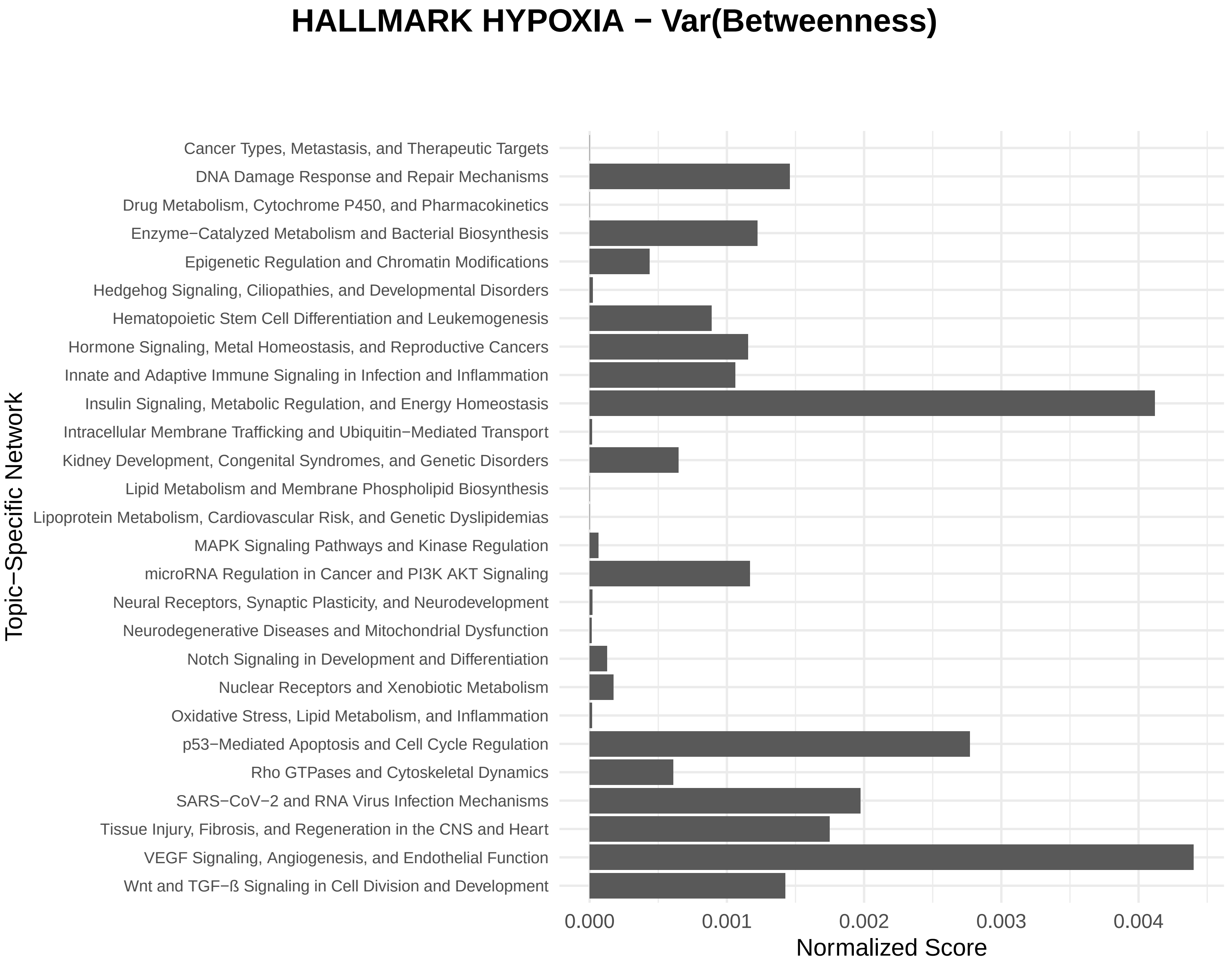
HALLMARK HYPOXIA − Var(Betweenness)
Topic−Specific Network
Cancer Types, Metastasis, and Therapeutic Targets
DNA Damage Response and Repair Mechanisms
Drug Metabolism, Cytochrome P450, and Pharmacokinetics
Enzyme−Catalyzed Metabolism and Bacterial Biosynthesis
Epigenetic Regulation and Chromatin Modifications
Hedgehog Signaling, Ciliopathies, and Developmental Disorders
Hematopoietic Stem Cell Differentiation and Leukemogenesis
Hormone Signaling, Metal Homeostasis, and Reproductive Cancers
Innate and Adaptive Immune Signaling in Infection and Inflammation
Insulin Signaling, Metabolic Regulation, and Energy Homeostasis
Intracellular Membrane Trafficking and Ubiquitin−Mediated Transport
Kidney Development, Congenital Syndromes, and Genetic Disorders
Lipid Metabolism and Membrane Phospholipid Biosynthesis
Lipoprotein Metabolism, Cardiovascular Risk, and Genetic Dyslipidemias
MAPK Signaling Pathways and Kinase Regulation
microRNA Regulation in Cancer and PI3K AKT Signaling
Neural Receptors, Synaptic Plasticity, and Neurodevelopment
Neurodegenerative Diseases and Mitochondrial Dysfunction
Notch Signaling in Development and Differentiation
Nuclear Receptors and Xenobiotic Metabolism
Oxidative Stress, Lipid Metabolism, and Inflammation
p53−Mediated Apoptosis and Cell Cycle Regulation
Rho GTPases and Cytoskeletal Dynamics
SARS−CoV−2 and RNA Virus Infection Mechanisms
Tissue Injury, Fibrosis, and Regeneration in the CNS and Heart
VEGF Signaling, Angiogenesis, and Endothelial Function
Wnt and TGF−ß Signaling in Cell Division and Development
0.000
0.001
0.002
0.003
0.004
Normalized Score

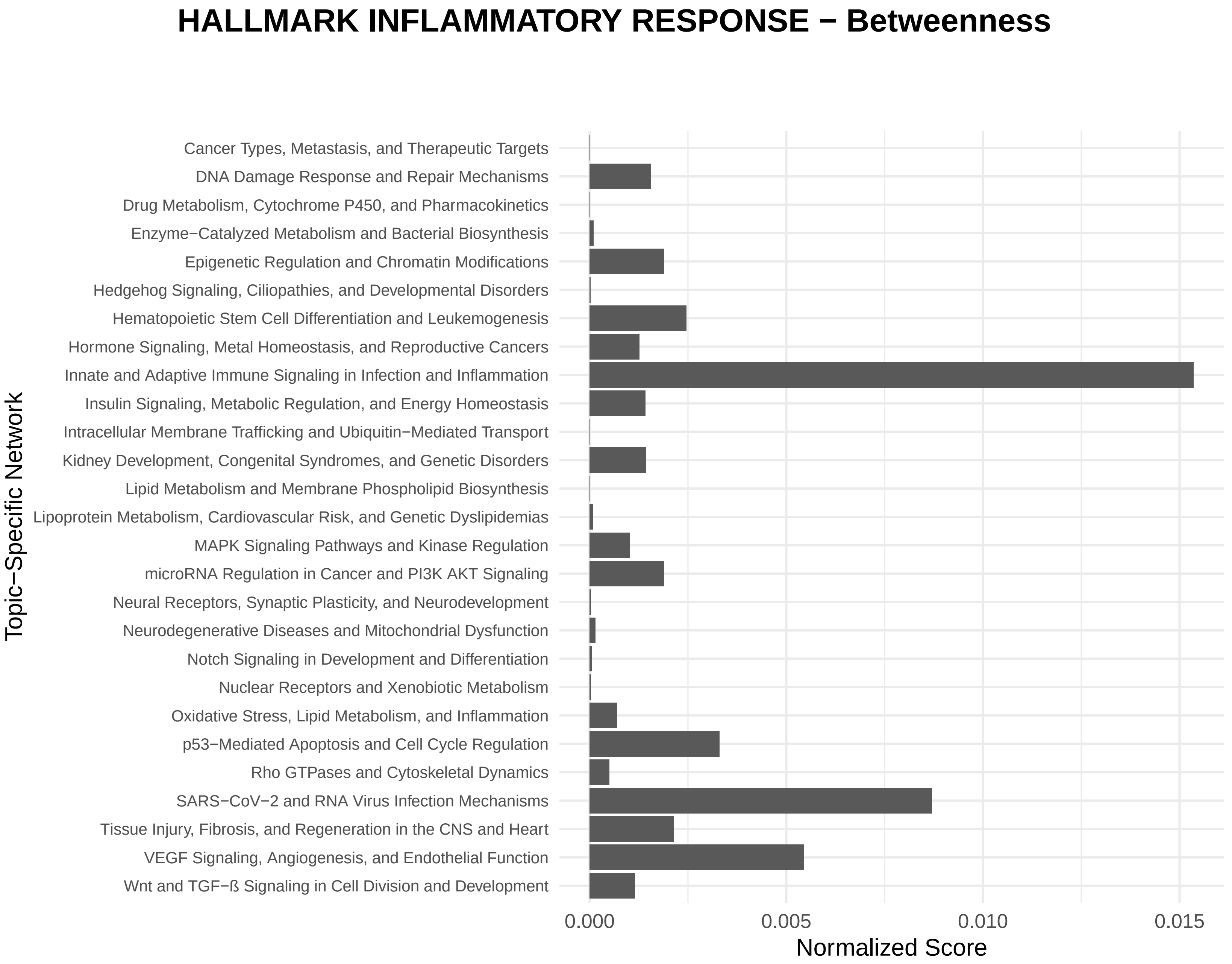

HALLMARK INFLAMMATORY RESPONSE − Betweenness
Topic−Specific Network
Cancer Types, Metastasis, and Therapeutic Targets
DNA Damage Response and Repair Mechanisms
Drug Metabolism, Cytochrome P450, and Pharmacokinetics
Enzyme−Catalyzed Metabolism and Bacterial Biosynthesis
Epigenetic Regulation and Chromatin Modifications
Hedgehog Signaling, Ciliopathies, and Developmental Disorders
Hematopoietic Stem Cell Differentiation and Leukemogenesis
Hormone Signaling, Metal Homeostasis, and Reproductive Cancers
Innate and Adaptive Immune Signaling in Infection and Inflammation
Insulin Signaling, Metabolic Regulation, and Energy Homeostasis
Intracellular Membrane Trafficking and Ubiquitin−Mediated Transport
Kidney Development, Congenital Syndromes, and Genetic Disorders
Lipid Metabolism and Membrane Phospholipid Biosynthesis
Lipoprotein Metabolism, Cardiovascular Risk, and Genetic Dyslipidemias
MAPK Signaling Pathways and Kinase Regulation
microRNA Regulation in Cancer and PI3K AKT Signaling
Neural Receptors, Synaptic Plasticity, and Neurodevelopment
Neurodegenerative Diseases and Mitochondrial Dysfunction
Notch Signaling in Development and Differentiation
Nuclear Receptors and Xenobiotic Metabolism
Oxidative Stress, Lipid Metabolism, and Inflammation
p53−Mediated Apoptosis and Cell Cycle Regulation
Rho GTPases and Cytoskeletal Dynamics
SARS−CoV−2 and RNA Virus Infection Mechanisms
Tissue Injury, Fibrosis, and Regeneration in the CNS and Heart
VEGF Signaling, Angiogenesis, and Endothelial Function
Wnt and TGF−ß Signaling in Cell Division and Development
0.000
0.005
0.010
0.015
Normalized Score

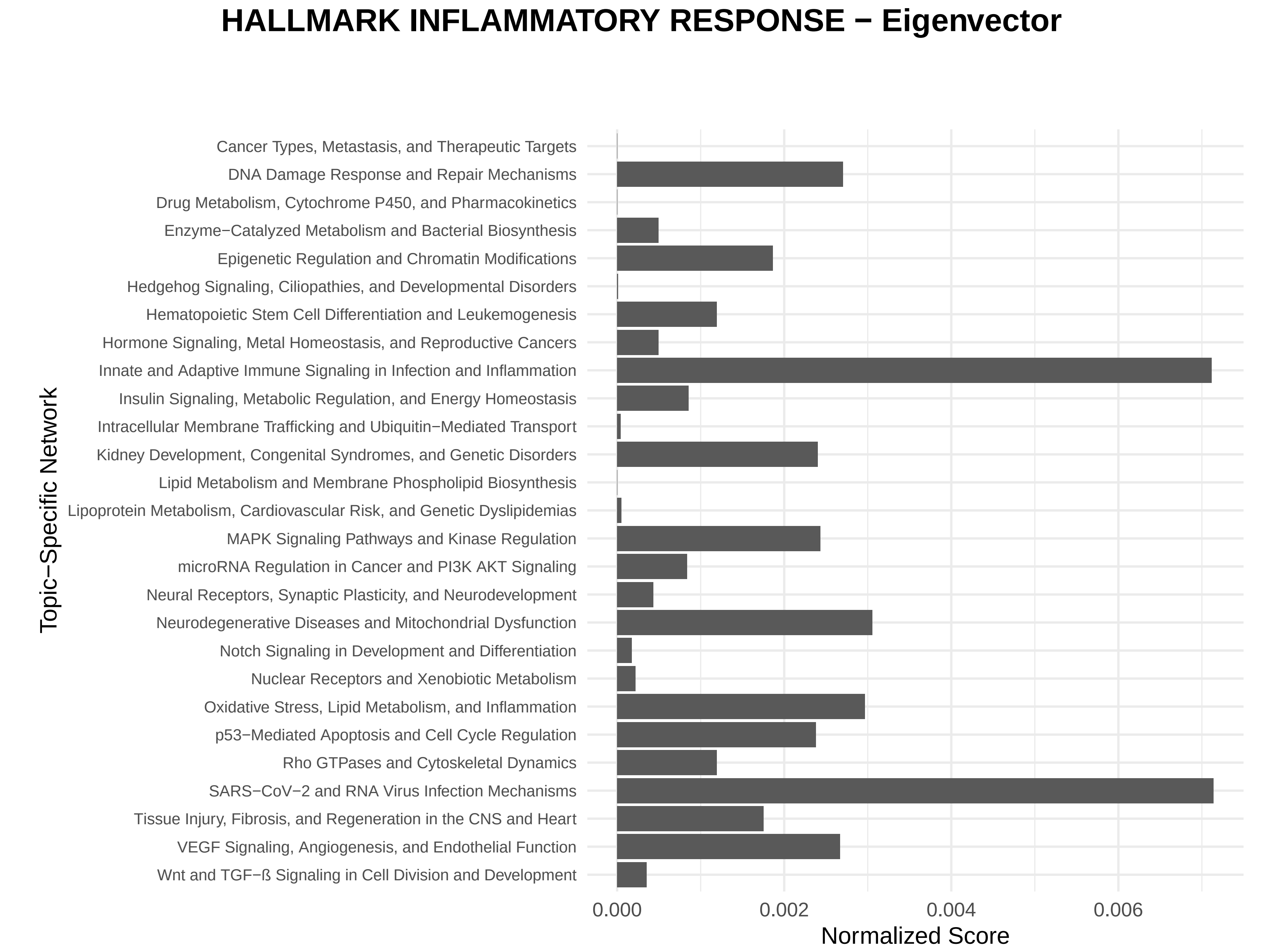
HALLMARK INFLAMMATORY RESPONSE − Eigenvector
Topic−Specific Network
Cancer Types, Metastasis, and Therapeutic Targets
DNA Damage Response and Repair Mechanisms
Drug Metabolism, Cytochrome P450, and Pharmacokinetics
Enzyme−Catalyzed Metabolism and Bacterial Biosynthesis
Epigenetic Regulation and Chromatin Modifications
Hedgehog Signaling, Ciliopathies, and Developmental Disorders
Hematopoietic Stem Cell Differentiation and Leukemogenesis
Hormone Signaling, Metal Homeostasis, and Reproductive Cancers
Innate and Adaptive Immune Signaling in Infection and Inflammation
Insulin Signaling, Metabolic Regulation, and Energy Homeostasis
Intracellular Membrane Trafficking and Ubiquitin−Mediated Transport
Kidney Development, Congenital Syndromes, and Genetic Disorders
Lipid Metabolism and Membrane Phospholipid Biosynthesis
Lipoprotein Metabolism, Cardiovascular Risk, and Genetic Dyslipidemias
MAPK Signaling Pathways and Kinase Regulation
microRNA Regulation in Cancer and PI3K AKT Signaling
Neural Receptors, Synaptic Plasticity, and Neurodevelopment
Neurodegenerative Diseases and Mitochondrial Dysfunction
Notch Signaling in Development and Differentiation
Nuclear Receptors and Xenobiotic Metabolism
Oxidative Stress, Lipid Metabolism, and Inflammation
p53−Mediated Apoptosis and Cell Cycle Regulation
Rho GTPases and Cytoskeletal Dynamics
SARS−CoV−2 and RNA Virus Infection Mechanisms
Tissue Injury, Fibrosis, and Regeneration in the CNS and Heart
VEGF Signaling, Angiogenesis, and Endothelial Function
Wnt and TGF−ß Signaling in Cell Division and Development
0.000
0.002
0.004
0.006
Normalized Score

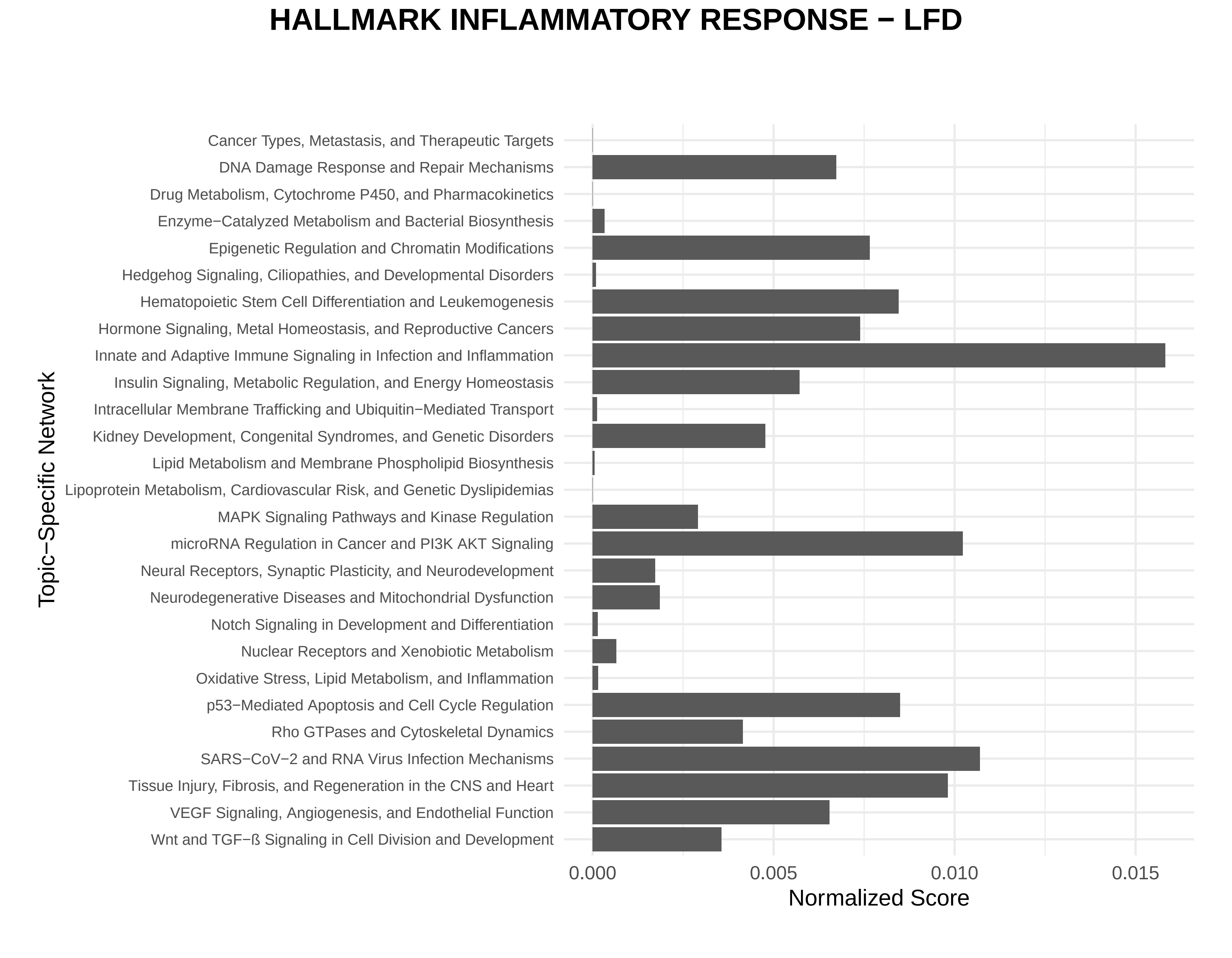
HALLMARK INFLAMMATORY RESPONSE − LFD
Topic−Specific Network
Cancer Types, Metastasis, and Therapeutic Targets
DNA Damage Response and Repair Mechanisms
Drug Metabolism, Cytochrome P450, and Pharmacokinetics
Enzyme−Catalyzed Metabolism and Bacterial Biosynthesis
Epigenetic Regulation and Chromatin Modifications
Hedgehog Signaling, Ciliopathies, and Developmental Disorders
Hematopoietic Stem Cell Differentiation and Leukemogenesis
Hormone Signaling, Metal Homeostasis, and Reproductive Cancers
Innate and Adaptive Immune Signaling in Infection and Inflammation
Insulin Signaling, Metabolic Regulation, and Energy Homeostasis
Intracellular Membrane Trafficking and Ubiquitin−Mediated Transport
Kidney Development, Congenital Syndromes, and Genetic Disorders
Lipid Metabolism and Membrane Phospholipid Biosynthesis
Lipoprotein Metabolism, Cardiovascular Risk, and Genetic Dyslipidemias
MAPK Signaling Pathways and Kinase Regulation
microRNA Regulation in Cancer and PI3K AKT Signaling
Neural Receptors, Synaptic Plasticity, and Neurodevelopment
Neurodegenerative Diseases and Mitochondrial Dysfunction
Notch Signaling in Development and Differentiation
Nuclear Receptors and Xenobiotic Metabolism
Oxidative Stress, Lipid Metabolism, and Inflammation
p53−Mediated Apoptosis and Cell Cycle Regulation
Rho GTPases and Cytoskeletal Dynamics
SARS−CoV−2 and RNA Virus Infection Mechanisms
Tissue Injury, Fibrosis, and Regeneration in the CNS and Heart
VEGF Signaling, Angiogenesis, and Endothelial Function
Wnt and TGF−ß Signaling in Cell Division and Development
0.000
0.005
0.010
0.015
Normalized Score

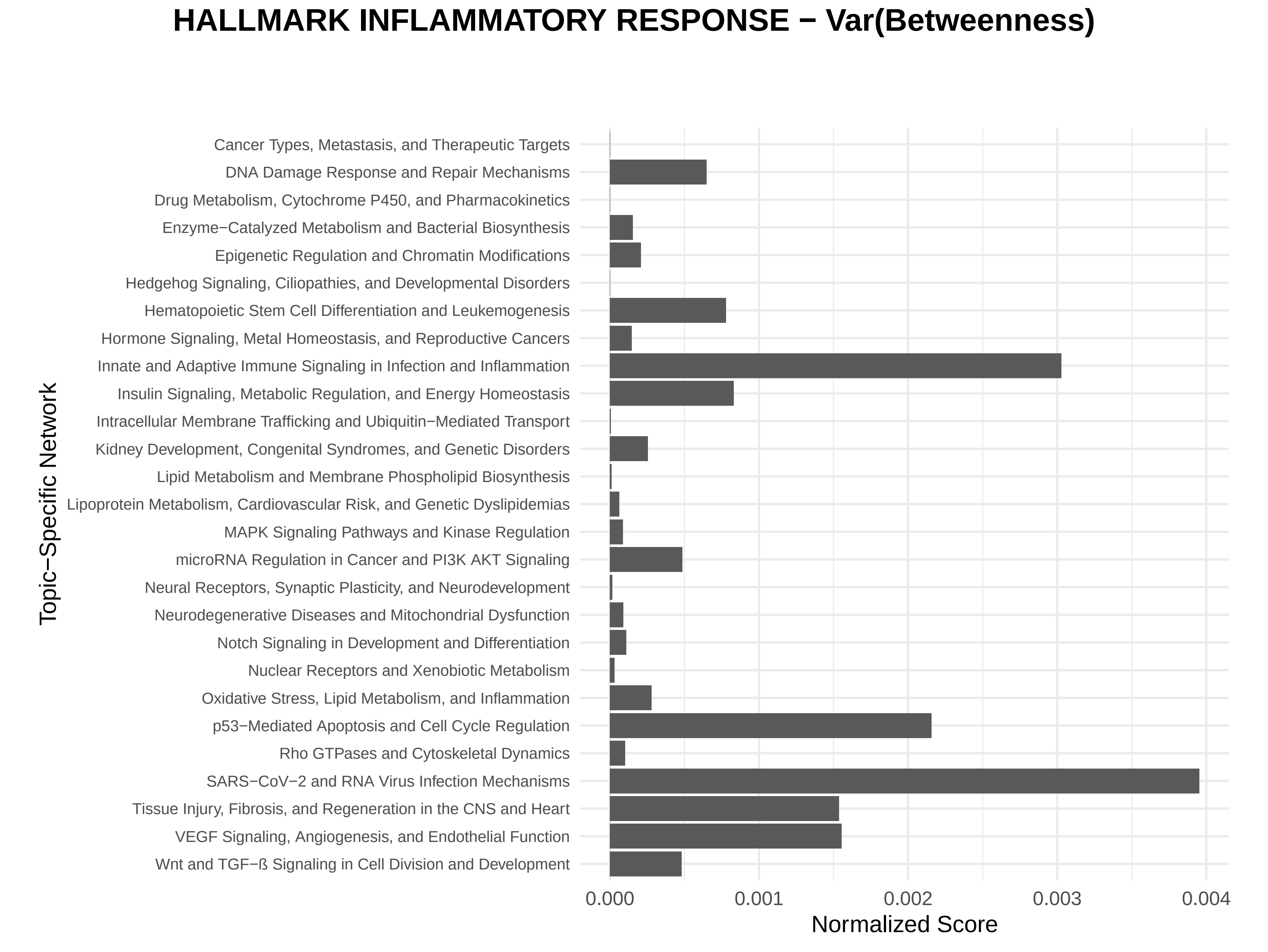
HALLMARK INFLAMMATORY RESPONSE − Var(Betweenness)
Topic−Specific Network
Normalized Score
Cancer Types, Metastasis, and Therapeutic Targets
DNA Damage Response and Repair Mechanisms
Drug Metabolism, Cytochrome P450, and Pharmacokinetics
Enzyme−Catalyzed Metabolism and Bacterial Biosynthesis
Epigenetic Regulation and Chromatin Modifications
Hedgehog Signaling, Ciliopathies, and Developmental Disorders
Hematopoietic Stem Cell Differentiation and Leukemogenesis
Hormone Signaling, Metal Homeostasis, and Reproductive Cancers
Innate and Adaptive Immune Signaling in Infection and Inflammation
Insulin Signaling, Metabolic Regulation, and Energy Homeostasis
Intracellular Membrane Trafficking and Ubiquitin−Mediated Transport
Kidney Development, Congenital Syndromes, and Genetic Disorders
Lipid Metabolism and Membrane Phospholipid Biosynthesis
Lipoprotein Metabolism, Cardiovascular Risk, and Genetic Dyslipidemias
MAPK Signaling Pathways and Kinase Regulation
microRNA Regulation in Cancer and PI3K AKT Signaling
Neural Receptors, Synaptic Plasticity, and Neurodevelopment
Neurodegenerative Diseases and Mitochondrial Dysfunction
Notch Signaling in Development and Differentiation
Nuclear Receptors and Xenobiotic Metabolism
Oxidative Stress, Lipid Metabolism, and Inflammation
p53−Mediated Apoptosis and Cell Cycle Regulation
Rho GTPases and Cytoskeletal Dynamics
SARS−CoV−2 and RNA Virus Infection Mechanisms
Tissue Injury, Fibrosis, and Regeneration in the CNS and Heart
VEGF Signaling, Angiogenesis, and Endothelial Function
Wnt and TGF−ß Signaling in Cell Division and Development
0.000
0.001
0.002
0.003
0.004

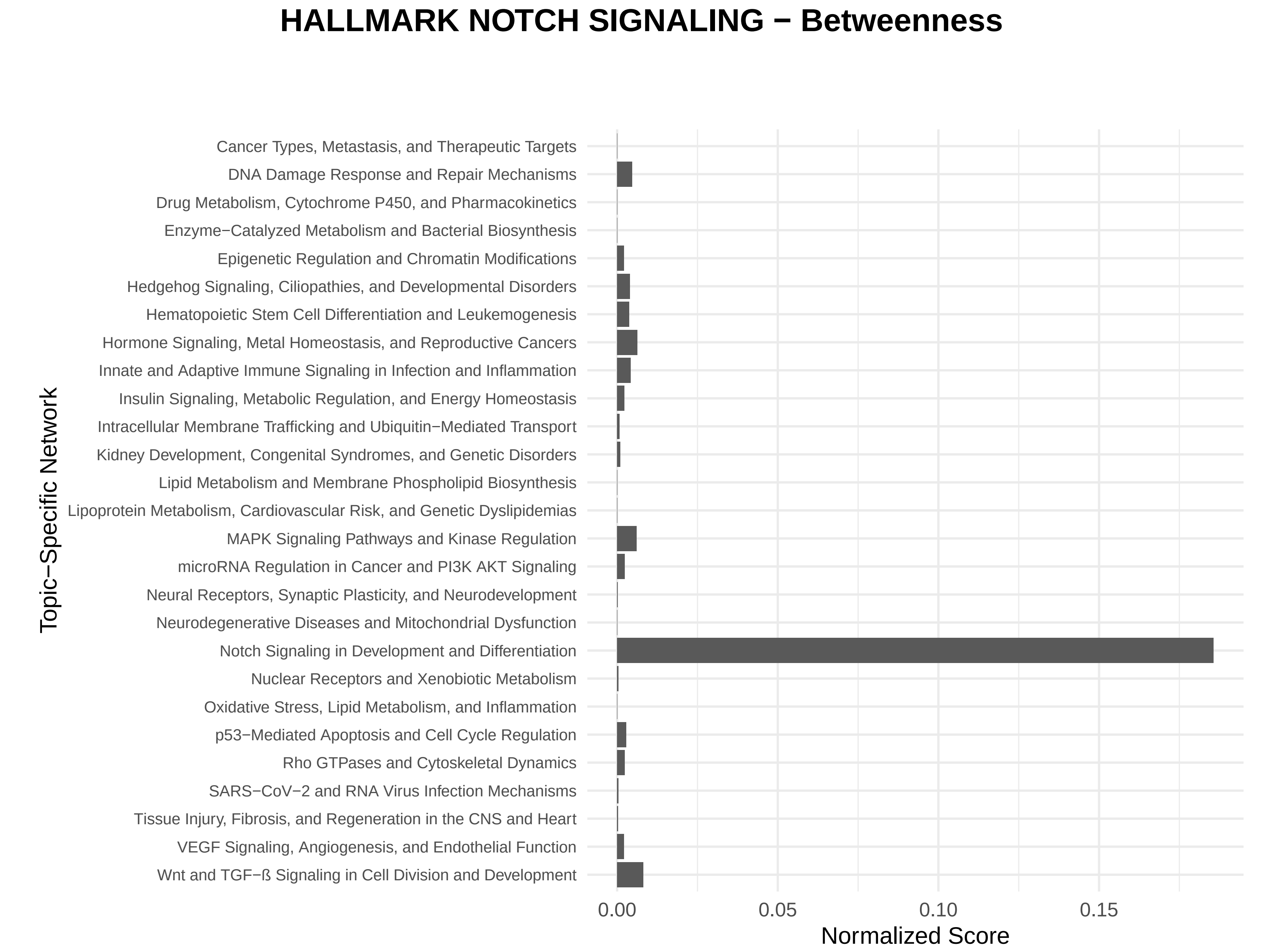
HALLMARK NOTCH SIGNALING − Betweenness
Topic−Specific Network
Cancer Types, Metastasis, and Therapeutic Targets
DNA Damage Response and Repair Mechanisms
Drug Metabolism, Cytochrome P450, and Pharmacokinetics
Enzyme−Catalyzed Metabolism and Bacterial Biosynthesis
Epigenetic Regulation and Chromatin Modifications
Hedgehog Signaling, Ciliopathies, and Developmental Disorders
Hematopoietic Stem Cell Differentiation and Leukemogenesis
Hormone Signaling, Metal Homeostasis, and Reproductive Cancers
Innate and Adaptive Immune Signaling in Infection and Inflammation
Insulin Signaling, Metabolic Regulation, and Energy Homeostasis
Intracellular Membrane Trafficking and Ubiquitin−Mediated Transport
Kidney Development, Congenital Syndromes, and Genetic Disorders
Lipid Metabolism and Membrane Phospholipid Biosynthesis
Lipoprotein Metabolism, Cardiovascular Risk, and Genetic Dyslipidemias
MAPK Signaling Pathways and Kinase Regulation
microRNA Regulation in Cancer and PI3K AKT Signaling
Neural Receptors, Synaptic Plasticity, and Neurodevelopment
Neurodegenerative Diseases and Mitochondrial Dysfunction
Notch Signaling in Development and Differentiation
Nuclear Receptors and Xenobiotic Metabolism
Oxidative Stress, Lipid Metabolism, and Inflammation
p53−Mediated Apoptosis and Cell Cycle Regulation
Rho GTPases and Cytoskeletal Dynamics
SARS−CoV−2 and RNA Virus Infection Mechanisms
Tissue Injury, Fibrosis, and Regeneration in the CNS and Heart
VEGF Signaling, Angiogenesis, and Endothelial Function
Wnt and TGF−ß Signaling in Cell Division and Development
0.00
0.05
0.10
0.15
Normalized Score

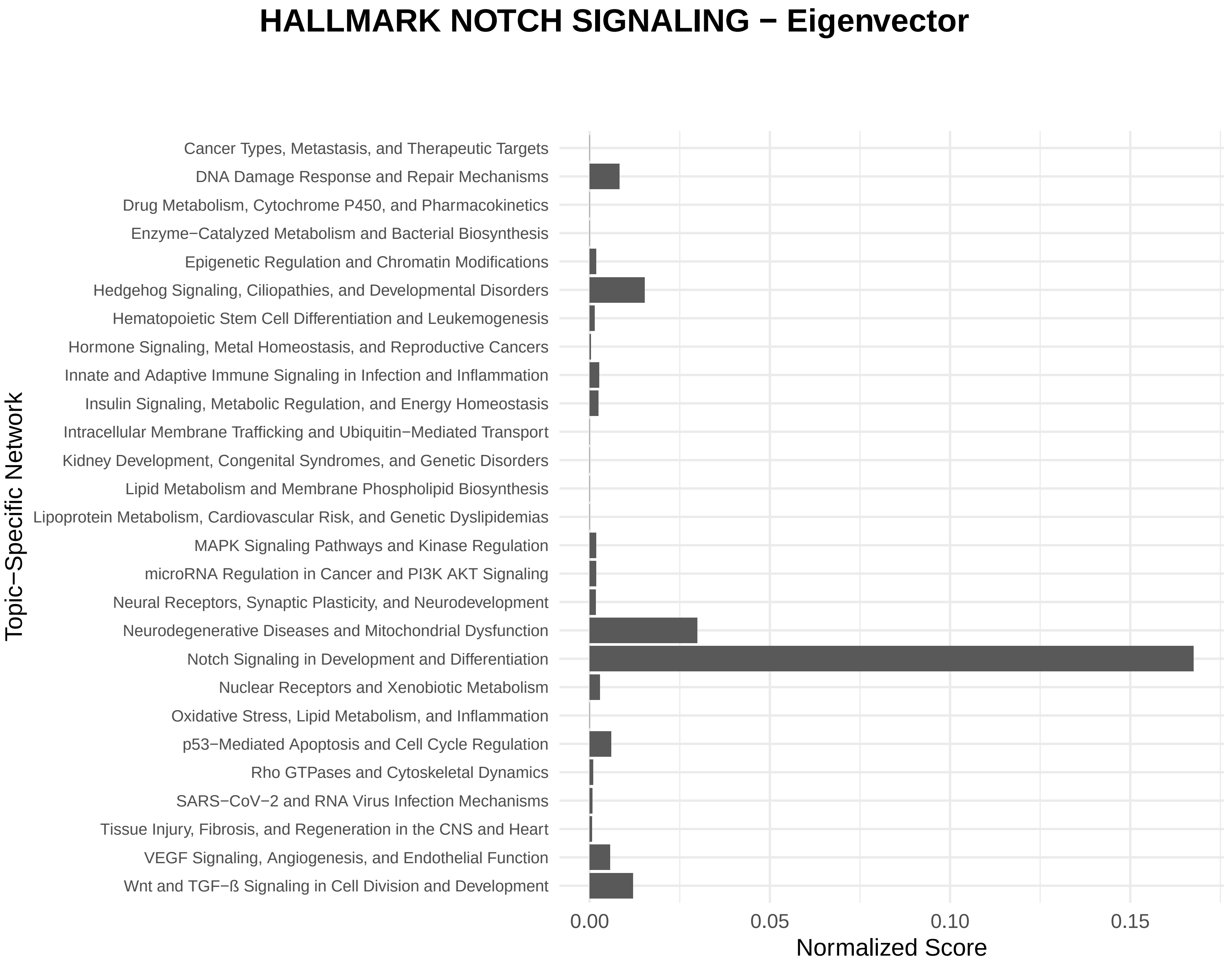
HALLMARK NOTCH SIGNALING − Eigenvector
Topic−Specific Network
Cancer Types, Metastasis, and Therapeutic Targets
DNA Damage Response and Repair Mechanisms
Drug Metabolism, Cytochrome P450, and Pharmacokinetics
Enzyme−Catalyzed Metabolism and Bacterial Biosynthesis
Epigenetic Regulation and Chromatin Modifications
Hedgehog Signaling, Ciliopathies, and Developmental Disorders
Hematopoietic Stem Cell Differentiation and Leukemogenesis
Hormone Signaling, Metal Homeostasis, and Reproductive Cancers
Innate and Adaptive Immune Signaling in Infection and Inflammation
Insulin Signaling, Metabolic Regulation, and Energy Homeostasis
Intracellular Membrane Trafficking and Ubiquitin−Mediated Transport
Kidney Development, Congenital Syndromes, and Genetic Disorders
Lipid Metabolism and Membrane Phospholipid Biosynthesis
Lipoprotein Metabolism, Cardiovascular Risk, and Genetic Dyslipidemias
MAPK Signaling Pathways and Kinase Regulation
microRNA Regulation in Cancer and PI3K AKT Signaling
Neural Receptors, Synaptic Plasticity, and Neurodevelopment
Neurodegenerative Diseases and Mitochondrial Dysfunction
Notch Signaling in Development and Differentiation
Nuclear Receptors and Xenobiotic Metabolism
Oxidative Stress, Lipid Metabolism, and Inflammation
p53−Mediated Apoptosis and Cell Cycle Regulation
Rho GTPases and Cytoskeletal Dynamics
SARS−CoV−2 and RNA Virus Infection Mechanisms
Tissue Injury, Fibrosis, and Regeneration in the CNS and Heart
VEGF Signaling, Angiogenesis, and Endothelial Function
Wnt and TGF−ß Signaling in Cell Division and Development
0.00
0.05
0.10
0.15
Normalized Score

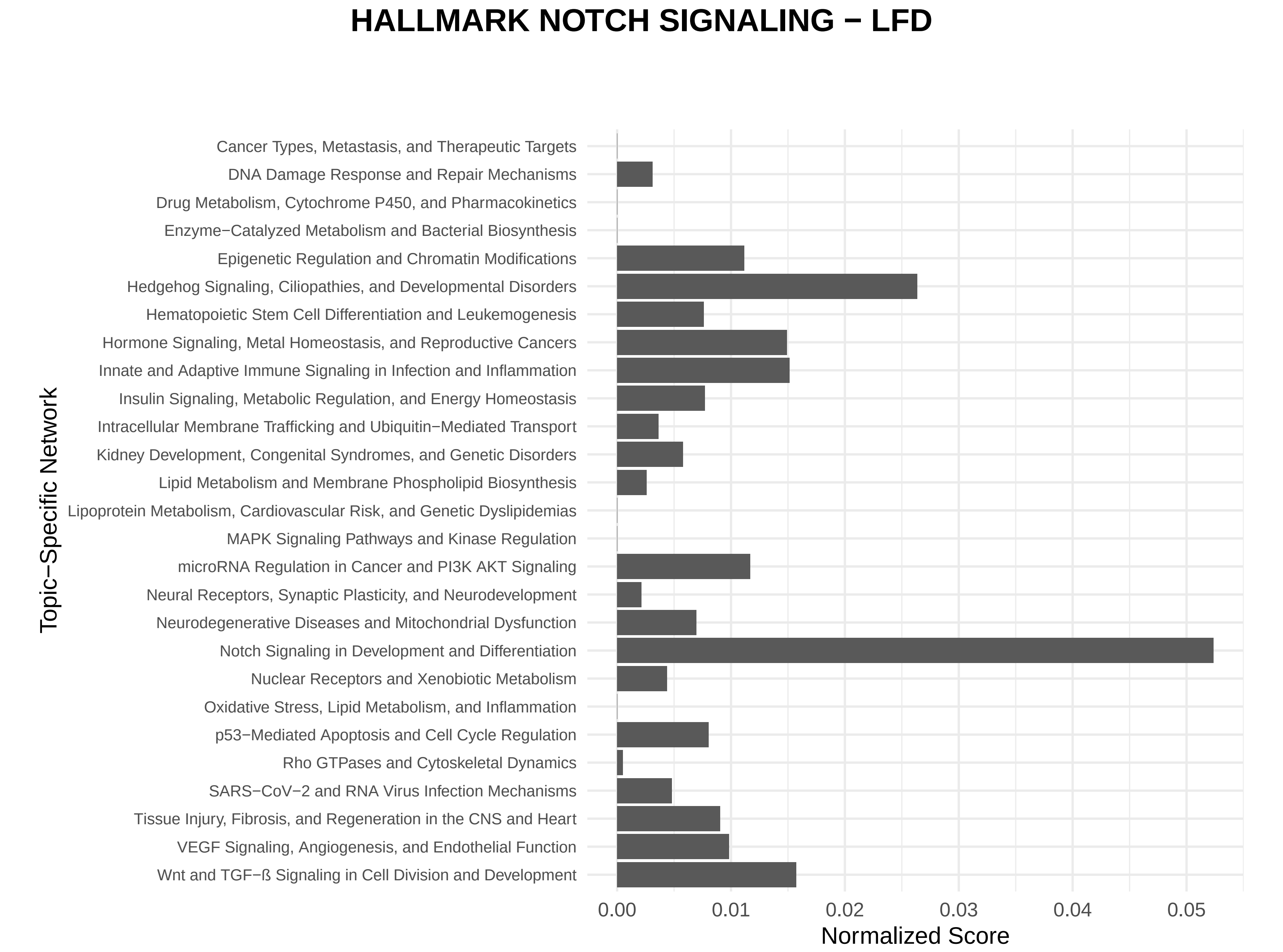
HALLMARK NOTCH SIGNALING − LFD
Topic−Specific Network
Cancer Types, Metastasis, and Therapeutic Targets
DNA Damage Response and Repair Mechanisms
Drug Metabolism, Cytochrome P450, and Pharmacokinetics
Enzyme−Catalyzed Metabolism and Bacterial Biosynthesis
Epigenetic Regulation and Chromatin Modifications
Hedgehog Signaling, Ciliopathies, and Developmental Disorders
Hematopoietic Stem Cell Differentiation and Leukemogenesis
Hormone Signaling, Metal Homeostasis, and Reproductive Cancers
Innate and Adaptive Immune Signaling in Infection and Inflammation
Insulin Signaling, Metabolic Regulation, and Energy Homeostasis
Intracellular Membrane Trafficking and Ubiquitin−Mediated Transport
Kidney Development, Congenital Syndromes, and Genetic Disorders
Lipid Metabolism and Membrane Phospholipid Biosynthesis
Lipoprotein Metabolism, Cardiovascular Risk, and Genetic Dyslipidemias
MAPK Signaling Pathways and Kinase Regulation
microRNA Regulation in Cancer and PI3K AKT Signaling
Neural Receptors, Synaptic Plasticity, and Neurodevelopment
Neurodegenerative Diseases and Mitochondrial Dysfunction
Notch Signaling in Development and Differentiation
Nuclear Receptors and Xenobiotic Metabolism
Oxidative Stress, Lipid Metabolism, and Inflammation
p53−Mediated Apoptosis and Cell Cycle Regulation
Rho GTPases and Cytoskeletal Dynamics
SARS−CoV−2 and RNA Virus Infection Mechanisms
Tissue Injury, Fibrosis, and Regeneration in the CNS and Heart
VEGF Signaling, Angiogenesis, and Endothelial Function
Wnt and TGF−ß Signaling in Cell Division and Development
0.00
0.01
0.02
0.03
0.04
0.05
Normalized Score

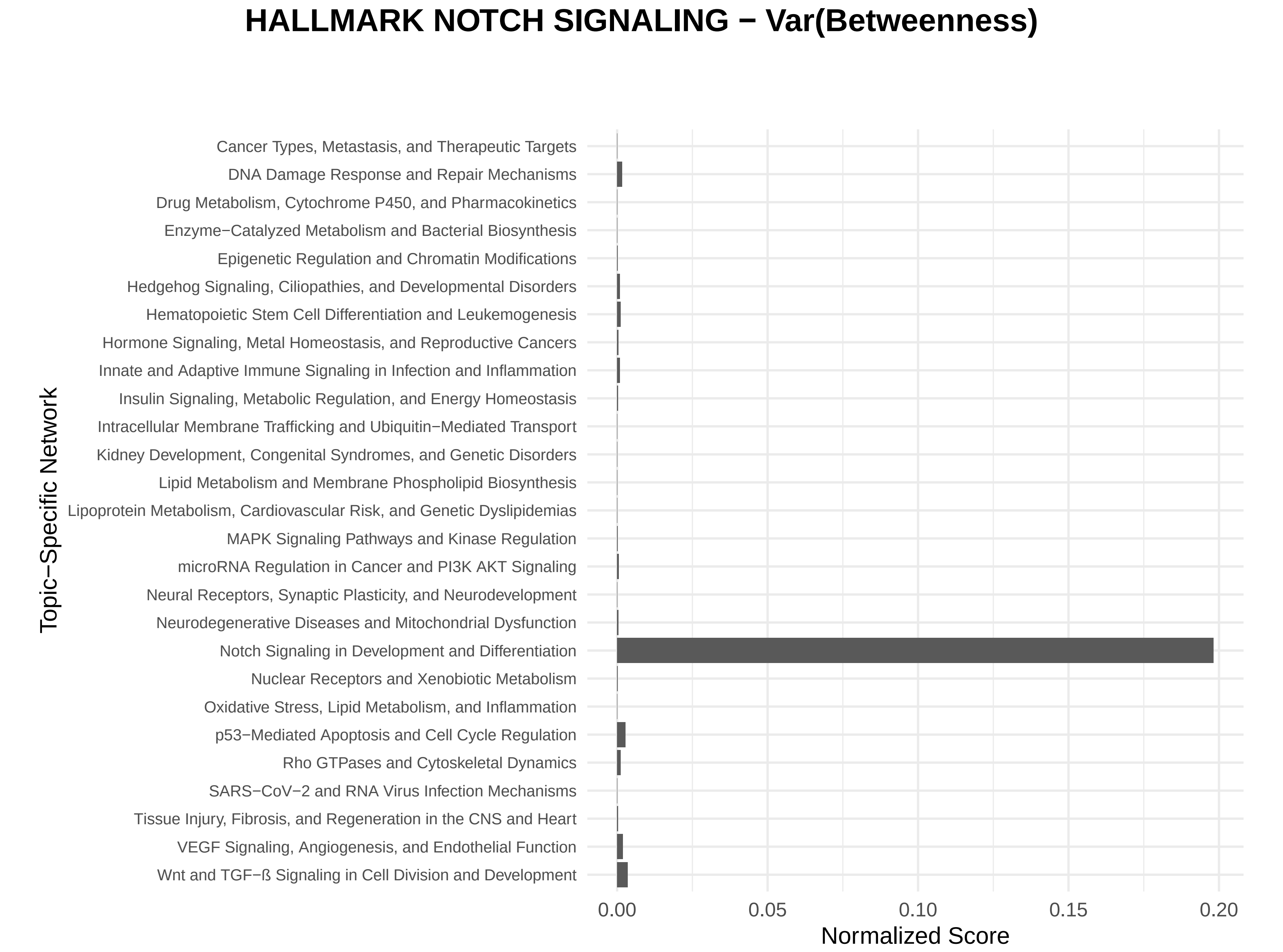

HALLMARK NOTCH SIGNALING − Var(Betweenness)
Topic−Specific Network
Cancer Types, Metastasis, and Therapeutic Targets
DNA Damage Response and Repair Mechanisms
Drug Metabolism, Cytochrome P450, and Pharmacokinetics
Enzyme−Catalyzed Metabolism and Bacterial Biosynthesis
Epigenetic Regulation and Chromatin Modifications
Hedgehog Signaling, Ciliopathies, and Developmental Disorders
Hematopoietic Stem Cell Differentiation and Leukemogenesis
Hormone Signaling, Metal Homeostasis, and Reproductive Cancers
Innate and Adaptive Immune Signaling in Infection and Inflammation
Insulin Signaling, Metabolic Regulation, and Energy Homeostasis
Intracellular Membrane Trafficking and Ubiquitin−Mediated Transport
Kidney Development, Congenital Syndromes, and Genetic Disorders
Lipid Metabolism and Membrane Phospholipid Biosynthesis
Lipoprotein Metabolism, Cardiovascular Risk, and Genetic Dyslipidemias
MAPK Signaling Pathways and Kinase Regulation
microRNA Regulation in Cancer and PI3K AKT Signaling
Neural Receptors, Synaptic Plasticity, and Neurodevelopment
Neurodegenerative Diseases and Mitochondrial Dysfunction
Notch Signaling in Development and Differentiation
Nuclear Receptors and Xenobiotic Metabolism
Oxidative Stress, Lipid Metabolism, and Inflammation
p53−Mediated Apoptosis and Cell Cycle Regulation
Rho GTPases and Cytoskeletal Dynamics
SARS−CoV−2 and RNA Virus Infection Mechanisms
Tissue Injury, Fibrosis, and Regeneration in the CNS and Heart
VEGF Signaling, Angiogenesis, and Endothelial Function
Wnt and TGF−ß Signaling in Cell Division and Development
0.00
0.05
0.10
0.15
0.20
Normalized Score

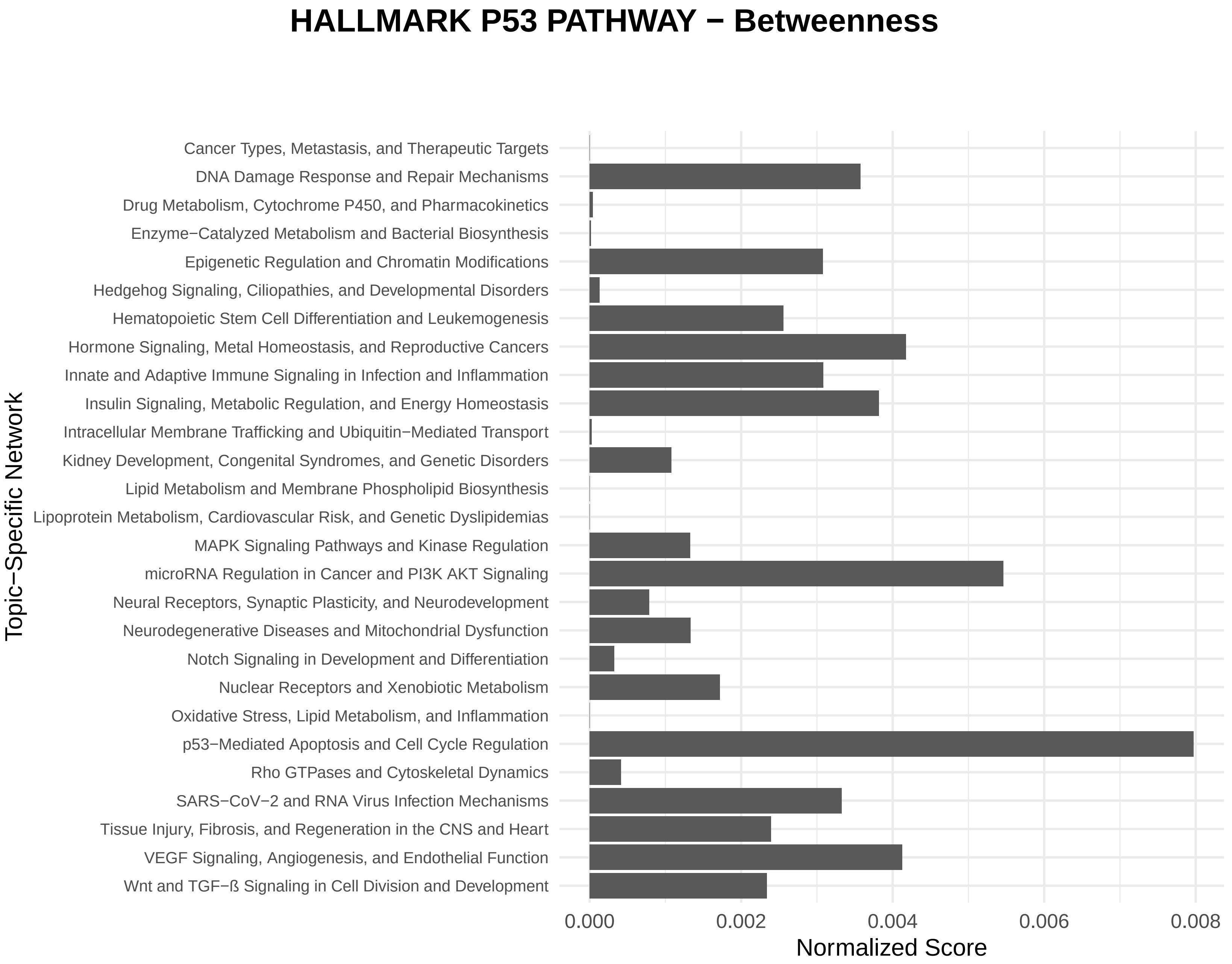
HALLMARK P53 PATHWAY − Betweenness
Topic−Specific Network
Cancer Types, Metastasis, and Therapeutic Targets
DNA Damage Response and Repair Mechanisms
Drug Metabolism, Cytochrome P450, and Pharmacokinetics
Enzyme−Catalyzed Metabolism and Bacterial Biosynthesis
Epigenetic Regulation and Chromatin Modifications
Hedgehog Signaling, Ciliopathies, and Developmental Disorders
Hematopoietic Stem Cell Differentiation and Leukemogenesis
Hormone Signaling, Metal Homeostasis, and Reproductive Cancers
Innate and Adaptive Immune Signaling in Infection and Inflammation
Insulin Signaling, Metabolic Regulation, and Energy Homeostasis
Intracellular Membrane Trafficking and Ubiquitin−Mediated Transport
Kidney Development, Congenital Syndromes, and Genetic Disorders
Lipid Metabolism and Membrane Phospholipid Biosynthesis
Lipoprotein Metabolism, Cardiovascular Risk, and Genetic Dyslipidemias
MAPK Signaling Pathways and Kinase Regulation
microRNA Regulation in Cancer and PI3K AKT Signaling
Neural Receptors, Synaptic Plasticity, and Neurodevelopment
Neurodegenerative Diseases and Mitochondrial Dysfunction
Notch Signaling in Development and Differentiation
Nuclear Receptors and Xenobiotic Metabolism
Oxidative Stress, Lipid Metabolism, and Inflammation
p53−Mediated Apoptosis and Cell Cycle Regulation
Rho GTPases and Cytoskeletal Dynamics
SARS−CoV−2 and RNA Virus Infection Mechanisms
Tissue Injury, Fibrosis, and Regeneration in the CNS and Heart
VEGF Signaling, Angiogenesis, and Endothelial Function
Wnt and TGF−ß Signaling in Cell Division and Development
0.000
0.002
0.004
0.006
0.008
Normalized Score

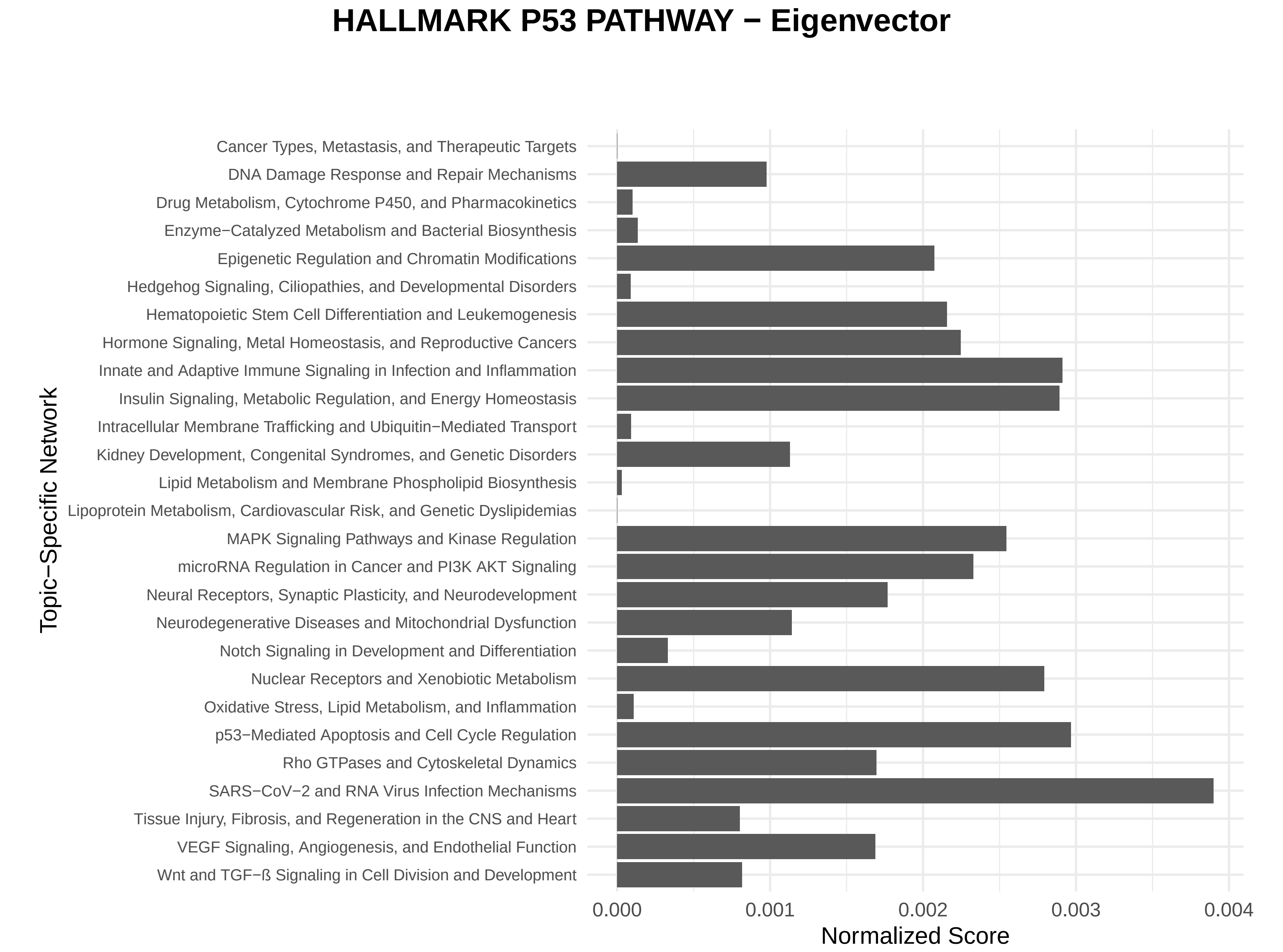
HALLMARK P53 PATHWAY − Eigenvector
Topic−Specific Network
Normalized Score
Cancer Types, Metastasis, and Therapeutic Targets
DNA Damage Response and Repair Mechanisms
Drug Metabolism, Cytochrome P450, and Pharmacokinetics
Enzyme−Catalyzed Metabolism and Bacterial Biosynthesis
Epigenetic Regulation and Chromatin Modifications
Hedgehog Signaling, Ciliopathies, and Developmental Disorders
Hematopoietic Stem Cell Differentiation and Leukemogenesis
Hormone Signaling, Metal Homeostasis, and Reproductive Cancers
Innate and Adaptive Immune Signaling in Infection and Inflammation
Insulin Signaling, Metabolic Regulation, and Energy Homeostasis
Intracellular Membrane Trafficking and Ubiquitin−Mediated Transport
Kidney Development, Congenital Syndromes, and Genetic Disorders
Lipid Metabolism and Membrane Phospholipid Biosynthesis
Lipoprotein Metabolism, Cardiovascular Risk, and Genetic Dyslipidemias
MAPK Signaling Pathways and Kinase Regulation
microRNA Regulation in Cancer and PI3K AKT Signaling
Neural Receptors, Synaptic Plasticity, and Neurodevelopment
Neurodegenerative Diseases and Mitochondrial Dysfunction
Notch Signaling in Development and Differentiation
Nuclear Receptors and Xenobiotic Metabolism
Oxidative Stress, Lipid Metabolism, and Inflammation
p53−Mediated Apoptosis and Cell Cycle Regulation
Rho GTPases and Cytoskeletal Dynamics
SARS−CoV−2 and RNA Virus Infection Mechanisms
Tissue Injury, Fibrosis, and Regeneration in the CNS and Heart
VEGF Signaling, Angiogenesis, and Endothelial Function
Wnt and TGF−ß Signaling in Cell Division and Development
0.000
0.001
0.002
0.003
0.004

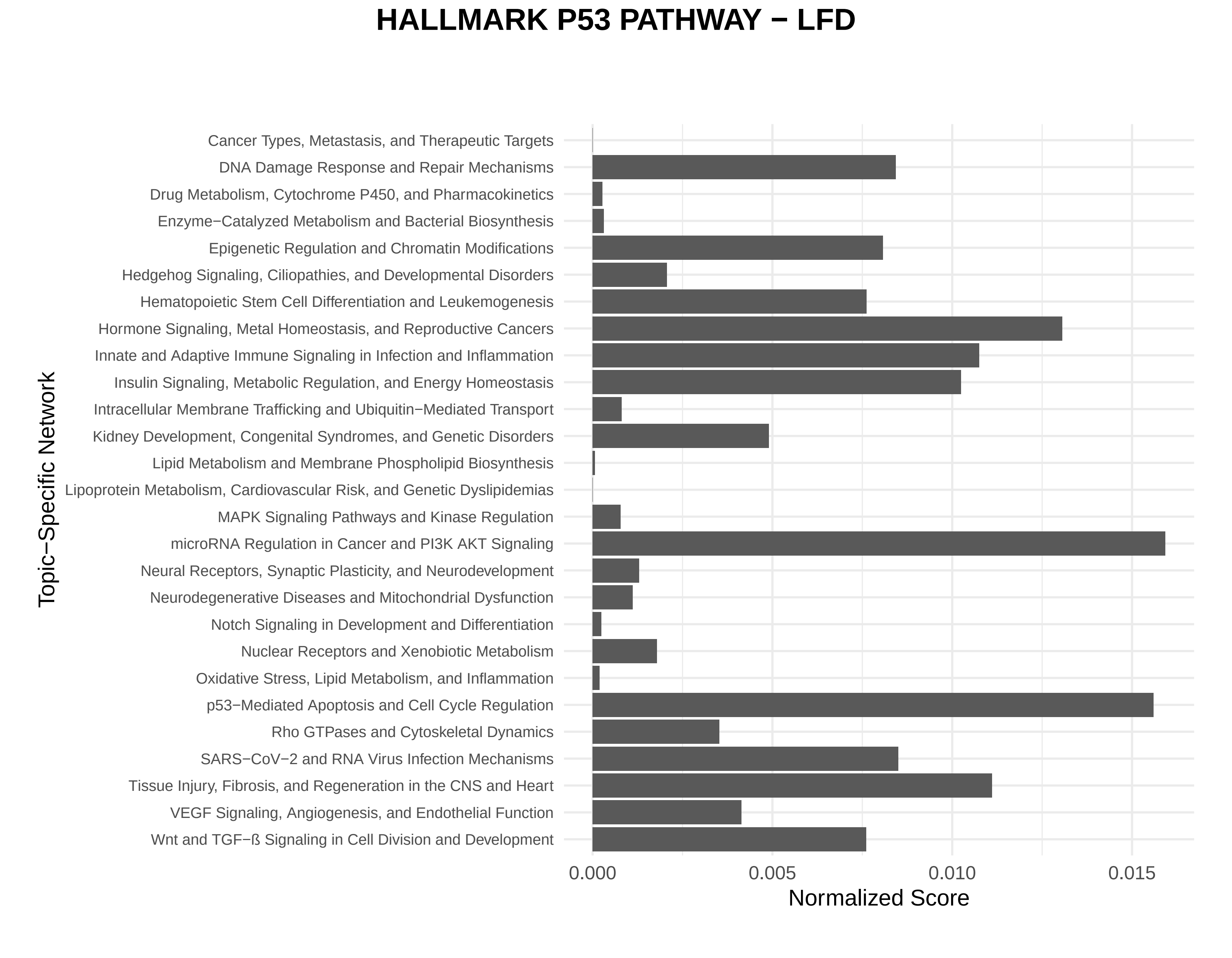

HALLMARK P53 PATHWAY − LFD
Topic−Specific Network
Cancer Types, Metastasis, and Therapeutic Targets
DNA Damage Response and Repair Mechanisms
Drug Metabolism, Cytochrome P450, and Pharmacokinetics
Enzyme−Catalyzed Metabolism and Bacterial Biosynthesis
Epigenetic Regulation and Chromatin Modifications
Hedgehog Signaling, Ciliopathies, and Developmental Disorders
Hematopoietic Stem Cell Differentiation and Leukemogenesis
Hormone Signaling, Metal Homeostasis, and Reproductive Cancers
Innate and Adaptive Immune Signaling in Infection and Inflammation
Insulin Signaling, Metabolic Regulation, and Energy Homeostasis
Intracellular Membrane Trafficking and Ubiquitin−Mediated Transport
Kidney Development, Congenital Syndromes, and Genetic Disorders
Lipid Metabolism and Membrane Phospholipid Biosynthesis
Lipoprotein Metabolism, Cardiovascular Risk, and Genetic Dyslipidemias
MAPK Signaling Pathways and Kinase Regulation
microRNA Regulation in Cancer and PI3K AKT Signaling
Neural Receptors, Synaptic Plasticity, and Neurodevelopment
Neurodegenerative Diseases and Mitochondrial Dysfunction
Notch Signaling in Development and Differentiation
Nuclear Receptors and Xenobiotic Metabolism
Oxidative Stress, Lipid Metabolism, and Inflammation
p53−Mediated Apoptosis and Cell Cycle Regulation
Rho GTPases and Cytoskeletal Dynamics
SARS−CoV−2 and RNA Virus Infection Mechanisms
Tissue Injury, Fibrosis, and Regeneration in the CNS and Heart
VEGF Signaling, Angiogenesis, and Endothelial Function
Wnt and TGF−ß Signaling in Cell Division and Development
0.000
0.005
0.010
0.015
Normalized Score

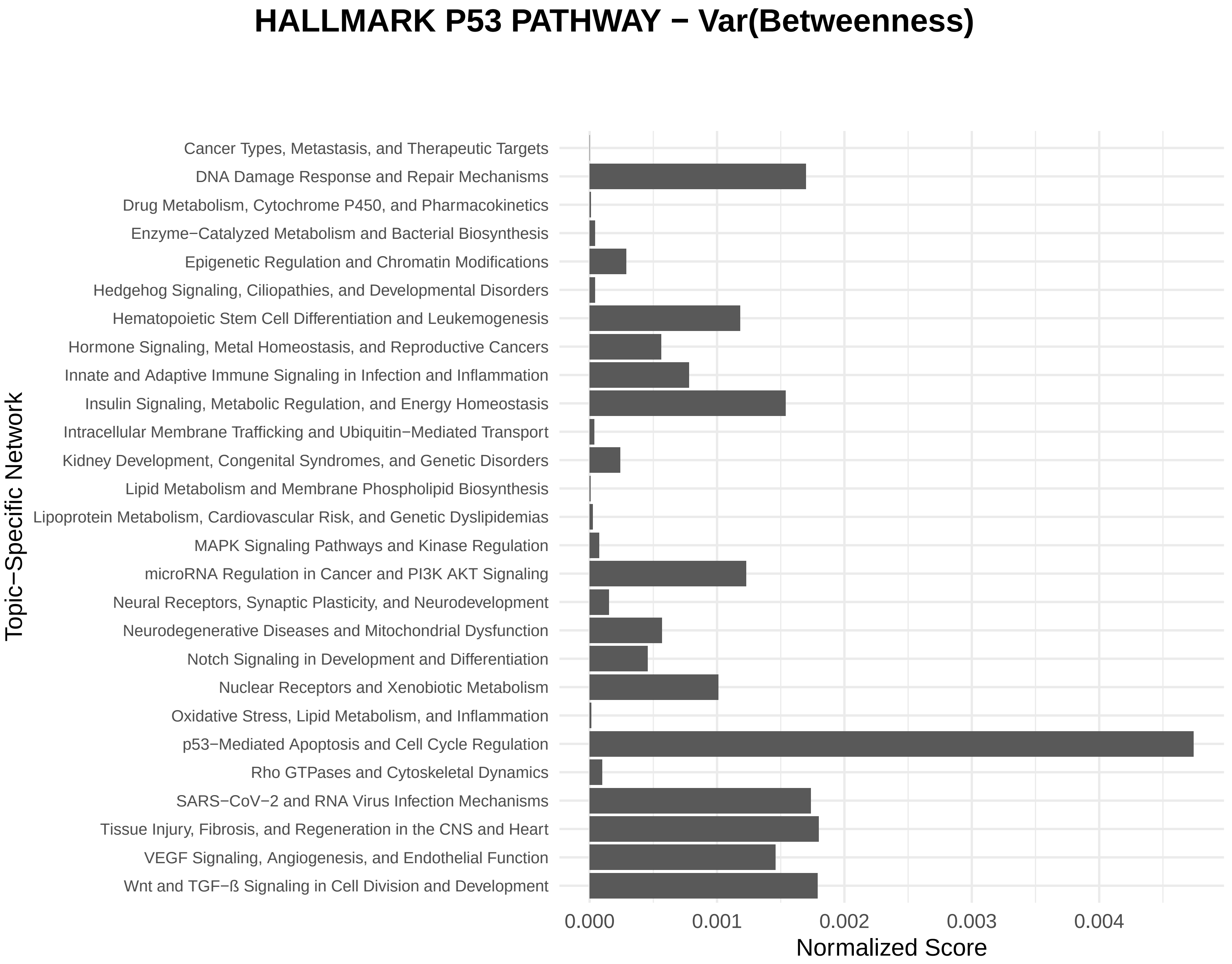
HALLMARK P53 PATHWAY − Var(Betweenness)
Topic−Specific Network
Cancer Types, Metastasis, and Therapeutic Targets
DNA Damage Response and Repair Mechanisms
Drug Metabolism, Cytochrome P450, and Pharmacokinetics
Enzyme−Catalyzed Metabolism and Bacterial Biosynthesis
Epigenetic Regulation and Chromatin Modifications
Hedgehog Signaling, Ciliopathies, and Developmental Disorders
Hematopoietic Stem Cell Differentiation and Leukemogenesis
Hormone Signaling, Metal Homeostasis, and Reproductive Cancers
Innate and Adaptive Immune Signaling in Infection and Inflammation
Insulin Signaling, Metabolic Regulation, and Energy Homeostasis
Intracellular Membrane Trafficking and Ubiquitin−Mediated Transport
Kidney Development, Congenital Syndromes, and Genetic Disorders
Lipid Metabolism and Membrane Phospholipid Biosynthesis
Lipoprotein Metabolism, Cardiovascular Risk, and Genetic Dyslipidemias
MAPK Signaling Pathways and Kinase Regulation
microRNA Regulation in Cancer and PI3K AKT Signaling
Neural Receptors, Synaptic Plasticity, and Neurodevelopment
Neurodegenerative Diseases and Mitochondrial Dysfunction
Notch Signaling in Development and Differentiation
Nuclear Receptors and Xenobiotic Metabolism
Oxidative Stress, Lipid Metabolism, and Inflammation
p53−Mediated Apoptosis and Cell Cycle Regulation
Rho GTPases and Cytoskeletal Dynamics
SARS−CoV−2 and RNA Virus Infection Mechanisms
Tissue Injury, Fibrosis, and Regeneration in the CNS and Heart
VEGF Signaling, Angiogenesis, and Endothelial Function
Wnt and TGF−ß Signaling in Cell Division and Development
0.000
0.001
0.002
0.003
0.004
Normalized Score

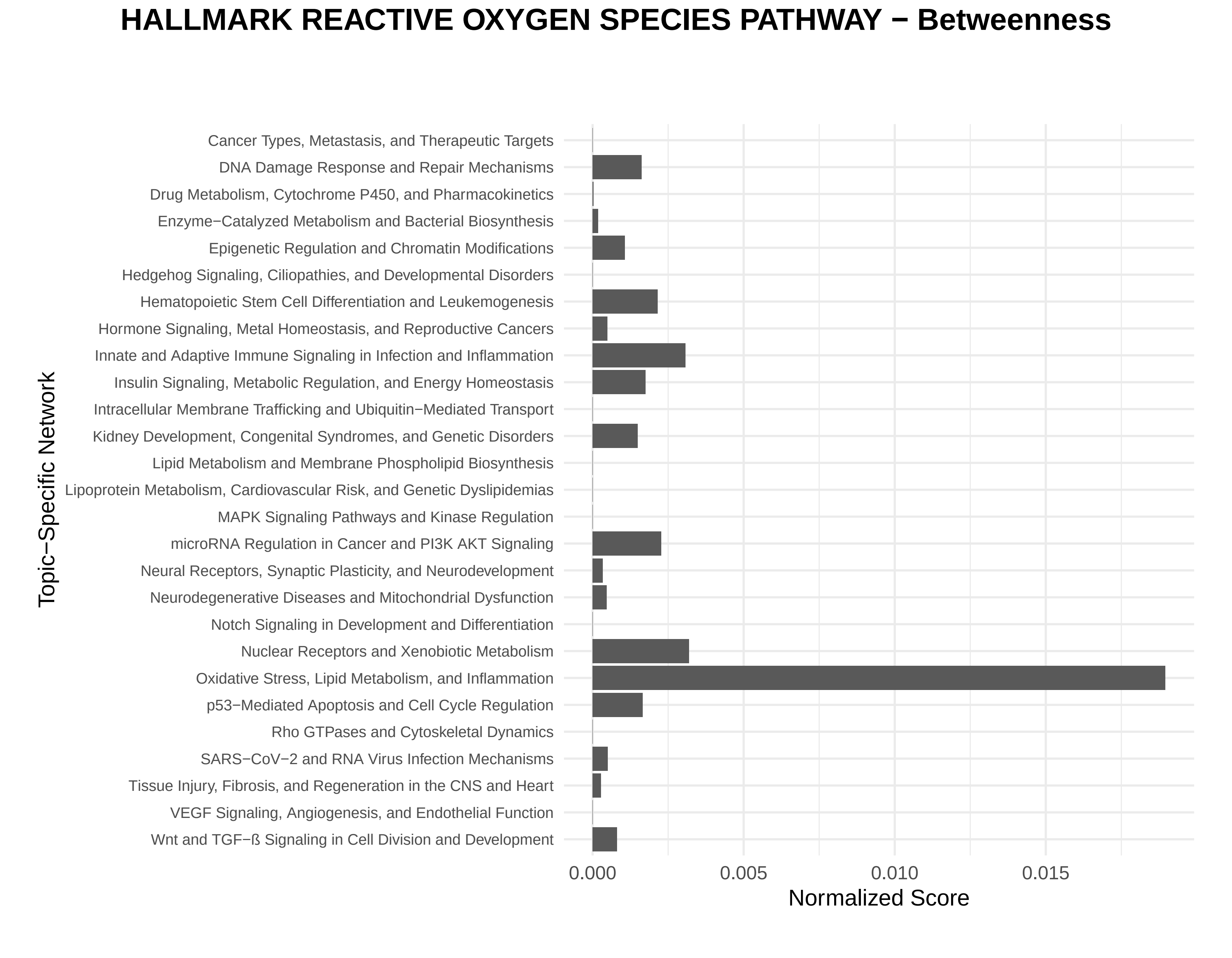
HALLMARK REACTIVE OXYGEN SPECIES PATHWAY − Betweenness
Topic−Specific Network
Cancer Types, Metastasis, and Therapeutic Targets
DNA Damage Response and Repair Mechanisms
Drug Metabolism, Cytochrome P450, and Pharmacokinetics
Enzyme−Catalyzed Metabolism and Bacterial Biosynthesis
Epigenetic Regulation and Chromatin Modifications
Hedgehog Signaling, Ciliopathies, and Developmental Disorders
Hematopoietic Stem Cell Differentiation and Leukemogenesis
Hormone Signaling, Metal Homeostasis, and Reproductive Cancers
Innate and Adaptive Immune Signaling in Infection and Inflammation
Insulin Signaling, Metabolic Regulation, and Energy Homeostasis
Intracellular Membrane Trafficking and Ubiquitin−Mediated Transport
Kidney Development, Congenital Syndromes, and Genetic Disorders
Lipid Metabolism and Membrane Phospholipid Biosynthesis
Lipoprotein Metabolism, Cardiovascular Risk, and Genetic Dyslipidemias
MAPK Signaling Pathways and Kinase Regulation
microRNA Regulation in Cancer and PI3K AKT Signaling
Neural Receptors, Synaptic Plasticity, and Neurodevelopment
Neurodegenerative Diseases and Mitochondrial Dysfunction
Notch Signaling in Development and Differentiation
Nuclear Receptors and Xenobiotic Metabolism
Oxidative Stress, Lipid Metabolism, and Inflammation
p53−Mediated Apoptosis and Cell Cycle Regulation
Rho GTPases and Cytoskeletal Dynamics
SARS−CoV−2 and RNA Virus Infection Mechanisms
Tissue Injury, Fibrosis, and Regeneration in the CNS and Heart
VEGF Signaling, Angiogenesis, and Endothelial Function
Wnt and TGF−ß Signaling in Cell Division and Development
0.000
0.005
0.010
0.015
Normalized Score

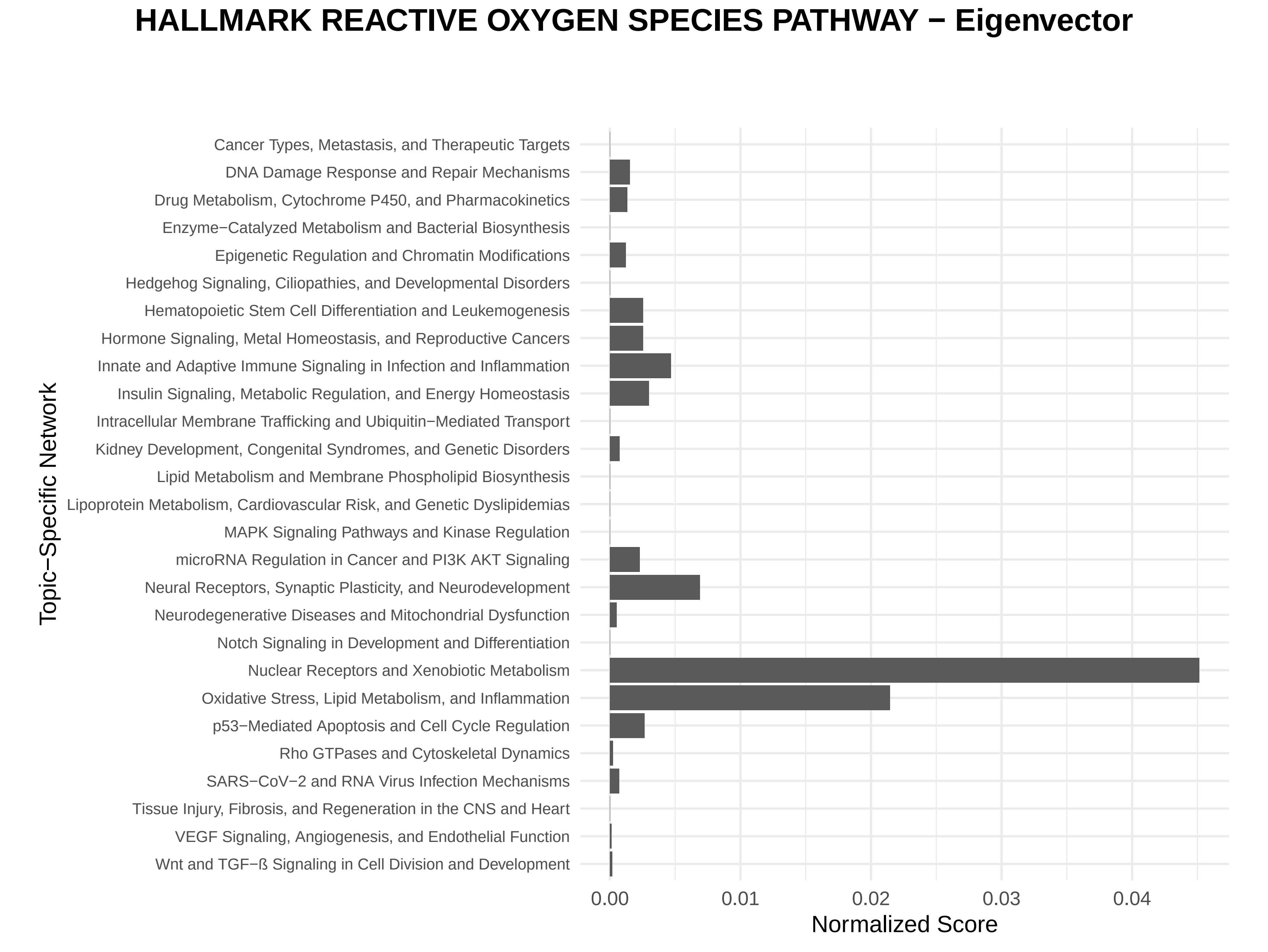
HALLMARK REACTIVE OXYGEN SPECIES PATHWAY − Eigenvector
Topic−Specific Network
Cancer Types, Metastasis, and Therapeutic Targets
DNA Damage Response and Repair Mechanisms
Drug Metabolism, Cytochrome P450, and Pharmacokinetics
Enzyme−Catalyzed Metabolism and Bacterial Biosynthesis
Epigenetic Regulation and Chromatin Modifications
Hedgehog Signaling, Ciliopathies, and Developmental Disorders
Hematopoietic Stem Cell Differentiation and Leukemogenesis
Hormone Signaling, Metal Homeostasis, and Reproductive Cancers
Innate and Adaptive Immune Signaling in Infection and Inflammation
Insulin Signaling, Metabolic Regulation, and Energy Homeostasis
Intracellular Membrane Trafficking and Ubiquitin−Mediated Transport
Kidney Development, Congenital Syndromes, and Genetic Disorders
Lipid Metabolism and Membrane Phospholipid Biosynthesis
Lipoprotein Metabolism, Cardiovascular Risk, and Genetic Dyslipidemias
MAPK Signaling Pathways and Kinase Regulation
microRNA Regulation in Cancer and PI3K AKT Signaling
Neural Receptors, Synaptic Plasticity, and Neurodevelopment
Neurodegenerative Diseases and Mitochondrial Dysfunction
Notch Signaling in Development and Differentiation
Nuclear Receptors and Xenobiotic Metabolism
Oxidative Stress, Lipid Metabolism, and Inflammation
p53−Mediated Apoptosis and Cell Cycle Regulation
Rho GTPases and Cytoskeletal Dynamics
SARS−CoV−2 and RNA Virus Infection Mechanisms
Tissue Injury, Fibrosis, and Regeneration in the CNS and Heart
VEGF Signaling, Angiogenesis, and Endothelial Function
Wnt and TGF−ß Signaling in Cell Division and Development
0.00
0.01
0.02
0.03
0.04
Normalized Score

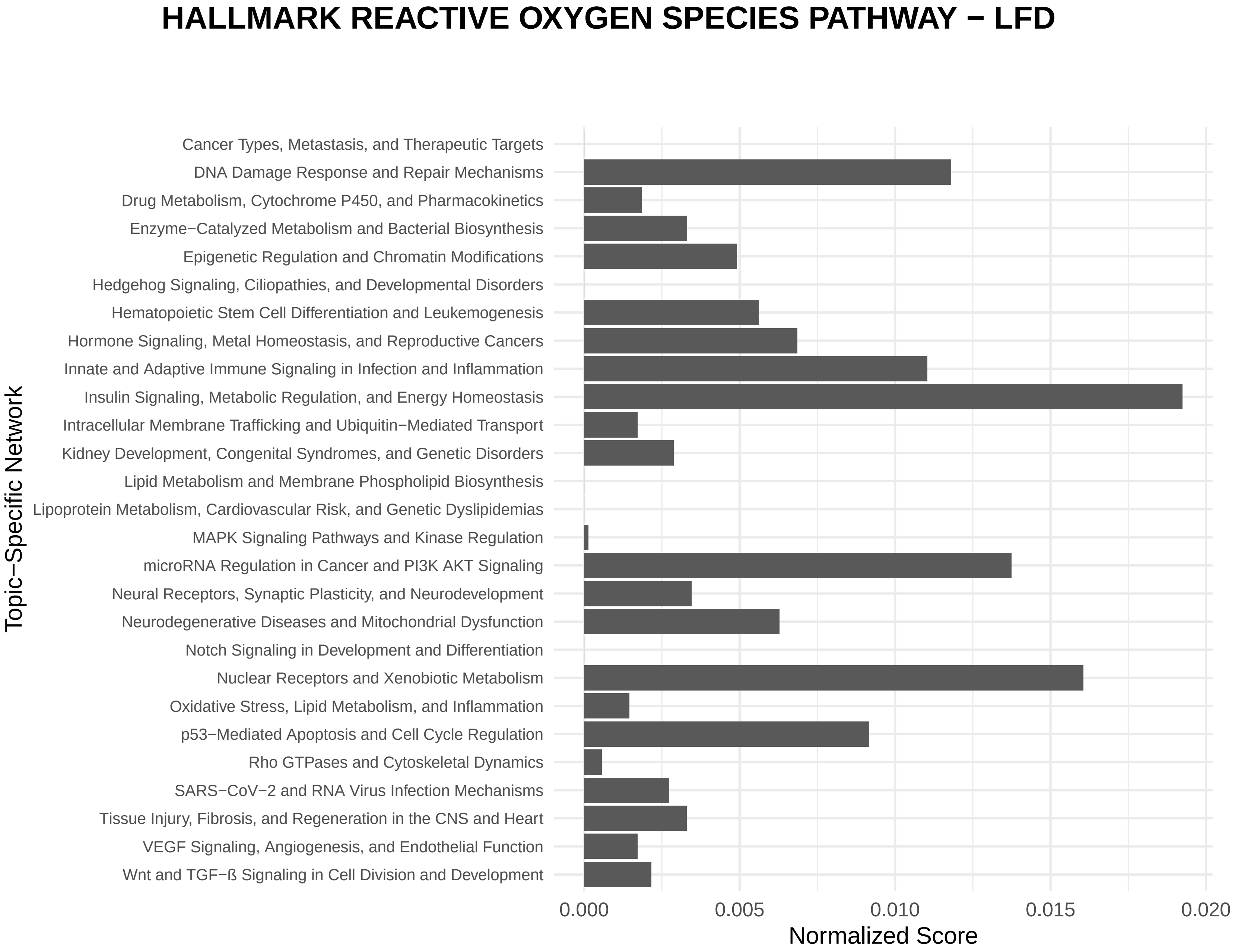
HALLMARK REACTIVE OXYGEN SPECIES PATHWAY − LFD
Topic−Specific Network
Cancer Types, Metastasis, and Therapeutic Targets
DNA Damage Response and Repair Mechanisms
Drug Metabolism, Cytochrome P450, and Pharmacokinetics
Enzyme−Catalyzed Metabolism and Bacterial Biosynthesis
Epigenetic Regulation and Chromatin Modifications
Hedgehog Signaling, Ciliopathies, and Developmental Disorders
Hematopoietic Stem Cell Differentiation and Leukemogenesis
Hormone Signaling, Metal Homeostasis, and Reproductive Cancers
Innate and Adaptive Immune Signaling in Infection and Inflammation
Insulin Signaling, Metabolic Regulation, and Energy Homeostasis
Intracellular Membrane Trafficking and Ubiquitin−Mediated Transport
Kidney Development, Congenital Syndromes, and Genetic Disorders
Lipid Metabolism and Membrane Phospholipid Biosynthesis
Lipoprotein Metabolism, Cardiovascular Risk, and Genetic Dyslipidemias
MAPK Signaling Pathways and Kinase Regulation
microRNA Regulation in Cancer and PI3K AKT Signaling
Neural Receptors, Synaptic Plasticity, and Neurodevelopment
Neurodegenerative Diseases and Mitochondrial Dysfunction
Notch Signaling in Development and Differentiation
Nuclear Receptors and Xenobiotic Metabolism
Oxidative Stress, Lipid Metabolism, and Inflammation
p53−Mediated Apoptosis and Cell Cycle Regulation
Rho GTPases and Cytoskeletal Dynamics
SARS−CoV−2 and RNA Virus Infection Mechanisms
Tissue Injury, Fibrosis, and Regeneration in the CNS and Heart
VEGF Signaling, Angiogenesis, and Endothelial Function
Wnt and TGF−ß Signaling in Cell Division and Development
0.000
0.005
0.010
0.015
0.020
Normalized Score

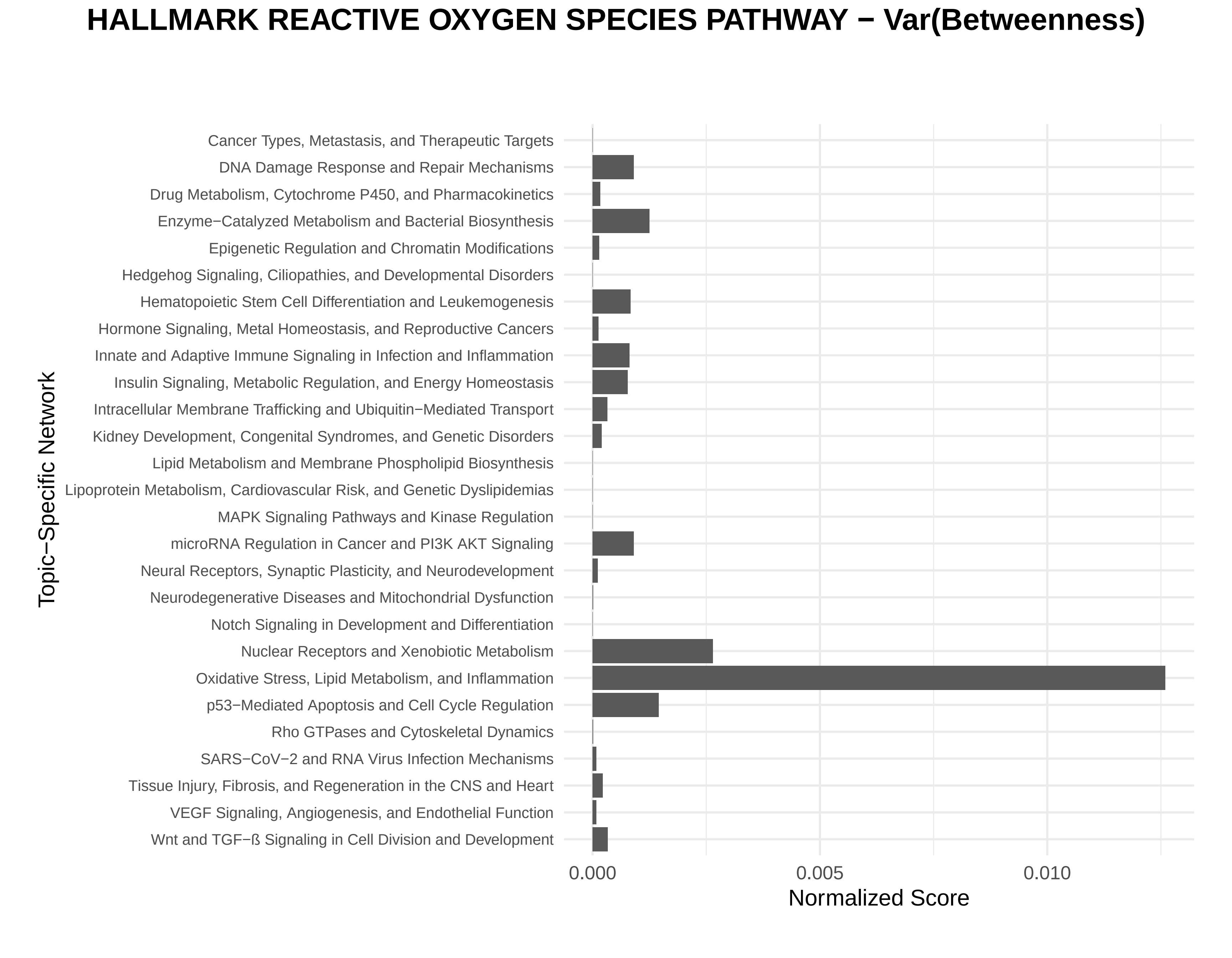
HALLMARK REACTIVE OXYGEN SPECIES PATHWAY − Var(Betweenness)
Cancer Types, Metastasis, and Therapeutic Targets
DNA Damage Response and Repair Mechanisms
Drug Metabolism, Cytochrome P450, and Pharmacokinetics
Enzyme−Catalyzed Metabolism and Bacterial Biosynthesis
Epigenetic Regulation and Chromatin Modifications
Hedgehog Signaling, Ciliopathies, and Developmental Disorders
Hematopoietic Stem Cell Differentiation and Leukemogenesis
Hormone Signaling, Metal Homeostasis, and Reproductive Cancers
Innate and Adaptive Immune Signaling in Infection and Inflammation
Insulin Signaling, Metabolic Regulation, and Energy Homeostasis
Intracellular Membrane Trafficking and Ubiquitin−Mediated Transport
Kidney Development, Congenital Syndromes, and Genetic Disorders
Lipid Metabolism and Membrane Phospholipid Biosynthesis
Lipoprotein Metabolism, Cardiovascular Risk, and Genetic Dyslipidemias
MAPK Signaling Pathways and Kinase Regulation
microRNA Regulation in Cancer and PI3K AKT Signaling
Neural Receptors, Synaptic Plasticity, and Neurodevelopment
Neurodegenerative Diseases and Mitochondrial Dysfunction
Notch Signaling in Development and Differentiation
Nuclear Receptors and Xenobiotic Metabolism
Oxidative Stress, Lipid Metabolism, and Inflammation
p53−Mediated Apoptosis and Cell Cycle Regulation
Rho GTPases and Cytoskeletal Dynamics
SARS−CoV−2 and RNA Virus Infection Mechanisms
Tissue Injury, Fibrosis, and Regeneration in the CNS and Heart
VEGF Signaling, Angiogenesis, and Endothelial Function
Wnt and TGF−ß Signaling in Cell Division and Development
Topic−Specific Network
0.000
0.005
0.010
Normalized Score

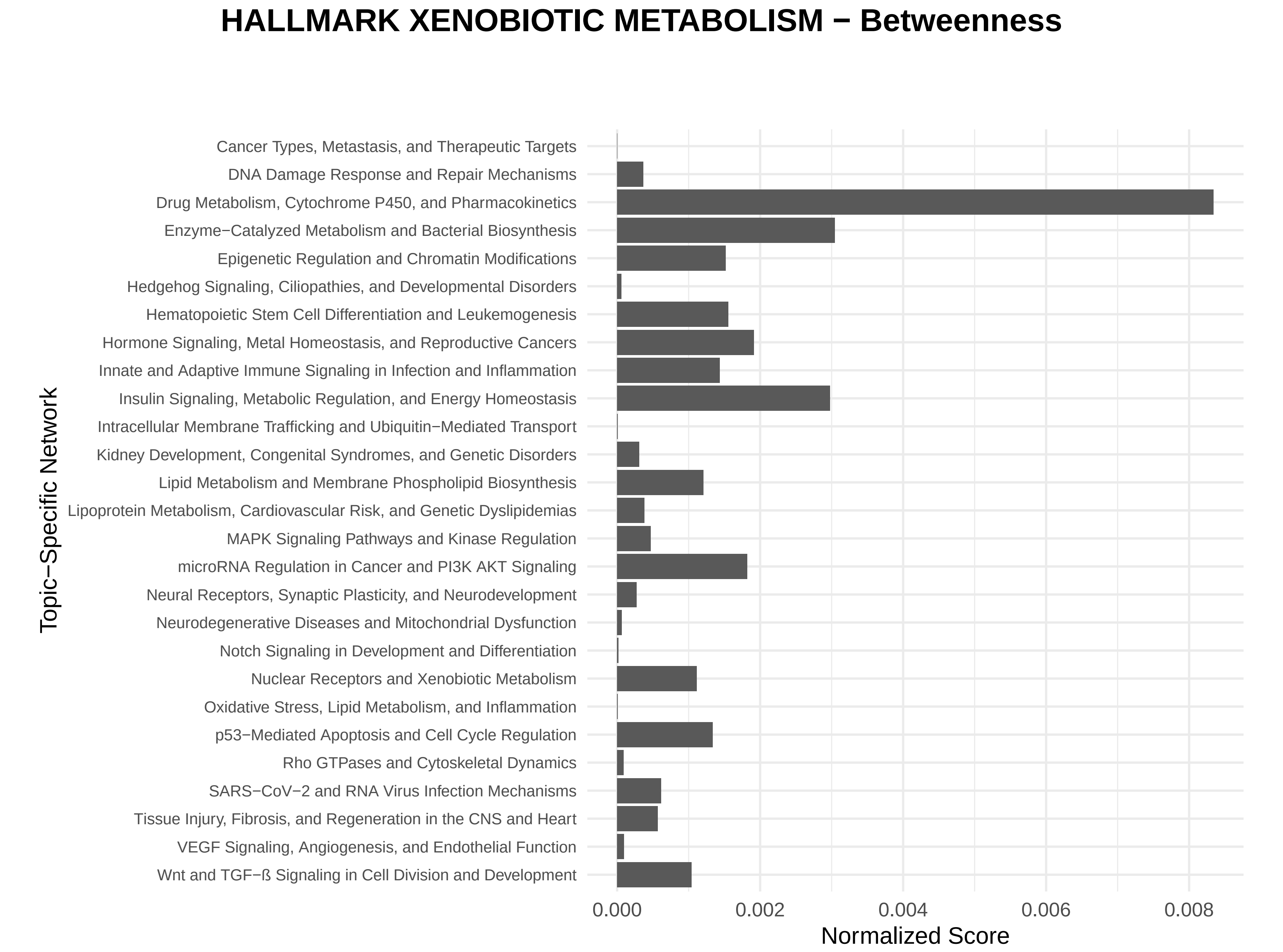
HALLMARK XENOBIOTIC METABOLISM − Betweenness
Cancer Types, Metastasis, and Therapeutic Targets
DNA Damage Response and Repair Mechanisms
Drug Metabolism, Cytochrome P450, and Pharmacokinetics
Enzyme−Catalyzed Metabolism and Bacterial Biosynthesis
Epigenetic Regulation and Chromatin Modifications
Hedgehog Signaling, Ciliopathies, and Developmental Disorders
Hematopoietic Stem Cell Differentiation and Leukemogenesis
Hormone Signaling, Metal Homeostasis, and Reproductive Cancers
Innate and Adaptive Immune Signaling in Infection and Inflammation
Insulin Signaling, Metabolic Regulation, and Energy Homeostasis
Intracellular Membrane Trafficking and Ubiquitin−Mediated Transport
Kidney Development, Congenital Syndromes, and Genetic Disorders
Lipid Metabolism and Membrane Phospholipid Biosynthesis
Lipoprotein Metabolism, Cardiovascular Risk, and Genetic Dyslipidemias
MAPK Signaling Pathways and Kinase Regulation
microRNA Regulation in Cancer and PI3K AKT Signaling
Neural Receptors, Synaptic Plasticity, and Neurodevelopment
Neurodegenerative Diseases and Mitochondrial Dysfunction
Notch Signaling in Development and Differentiation
Nuclear Receptors and Xenobiotic Metabolism
Oxidative Stress, Lipid Metabolism, and Inflammation
p53−Mediated Apoptosis and Cell Cycle Regulation
Rho GTPases and Cytoskeletal Dynamics
SARS−CoV−2 and RNA Virus Infection Mechanisms
Tissue Injury, Fibrosis, and Regeneration in the CNS and Heart
VEGF Signaling, Angiogenesis, and Endothelial Function
Wnt and TGF−ß Signaling in Cell Division and Development
Topic−Specific Network
0.000
0.002
0.004
0.006
0.008
Normalized Score

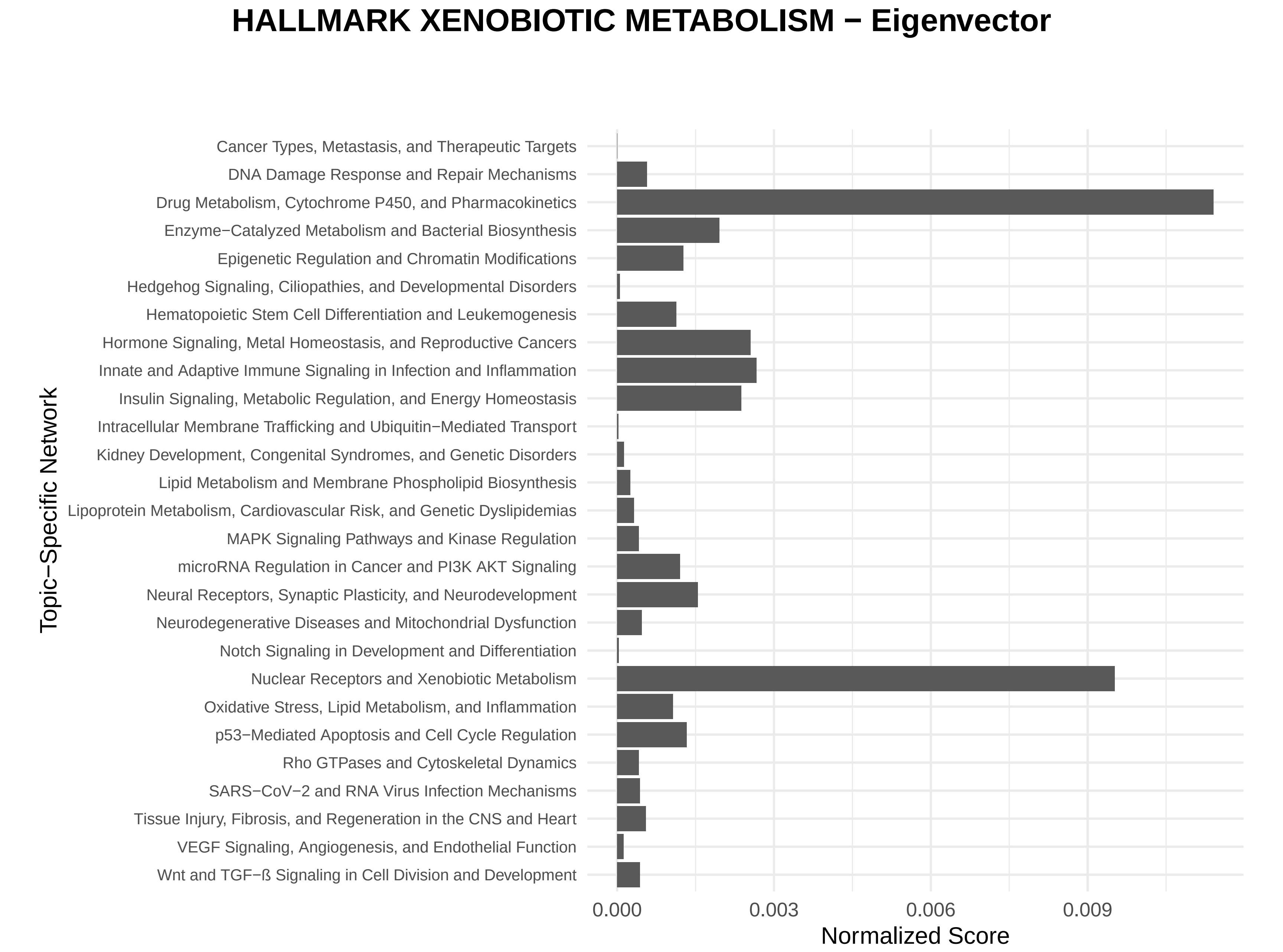
HALLMARK XENOBIOTIC METABOLISM − Eigenvector
Topic−Specific Network
Cancer Types, Metastasis, and Therapeutic Targets
DNA Damage Response and Repair Mechanisms
Drug Metabolism, Cytochrome P450, and Pharmacokinetics
Enzyme−Catalyzed Metabolism and Bacterial Biosynthesis
Epigenetic Regulation and Chromatin Modifications
Hedgehog Signaling, Ciliopathies, and Developmental Disorders
Hematopoietic Stem Cell Differentiation and Leukemogenesis
Hormone Signaling, Metal Homeostasis, and Reproductive Cancers
Innate and Adaptive Immune Signaling in Infection and Inflammation
Insulin Signaling, Metabolic Regulation, and Energy Homeostasis
Intracellular Membrane Trafficking and Ubiquitin−Mediated Transport
Kidney Development, Congenital Syndromes, and Genetic Disorders
Lipid Metabolism and Membrane Phospholipid Biosynthesis
Lipoprotein Metabolism, Cardiovascular Risk, and Genetic Dyslipidemias
MAPK Signaling Pathways and Kinase Regulation
microRNA Regulation in Cancer and PI3K AKT Signaling
Neural Receptors, Synaptic Plasticity, and Neurodevelopment
Neurodegenerative Diseases and Mitochondrial Dysfunction
Notch Signaling in Development and Differentiation
Nuclear Receptors and Xenobiotic Metabolism
Oxidative Stress, Lipid Metabolism, and Inflammation
p53−Mediated Apoptosis and Cell Cycle Regulation
Rho GTPases and Cytoskeletal Dynamics
SARS−CoV−2 and RNA Virus Infection Mechanisms
Tissue Injury, Fibrosis, and Regeneration in the CNS and Heart
VEGF Signaling, Angiogenesis, and Endothelial Function
Wnt and TGF−ß Signaling in Cell Division and Development
0.000
0.003
0.006
0.009
Normalized Score

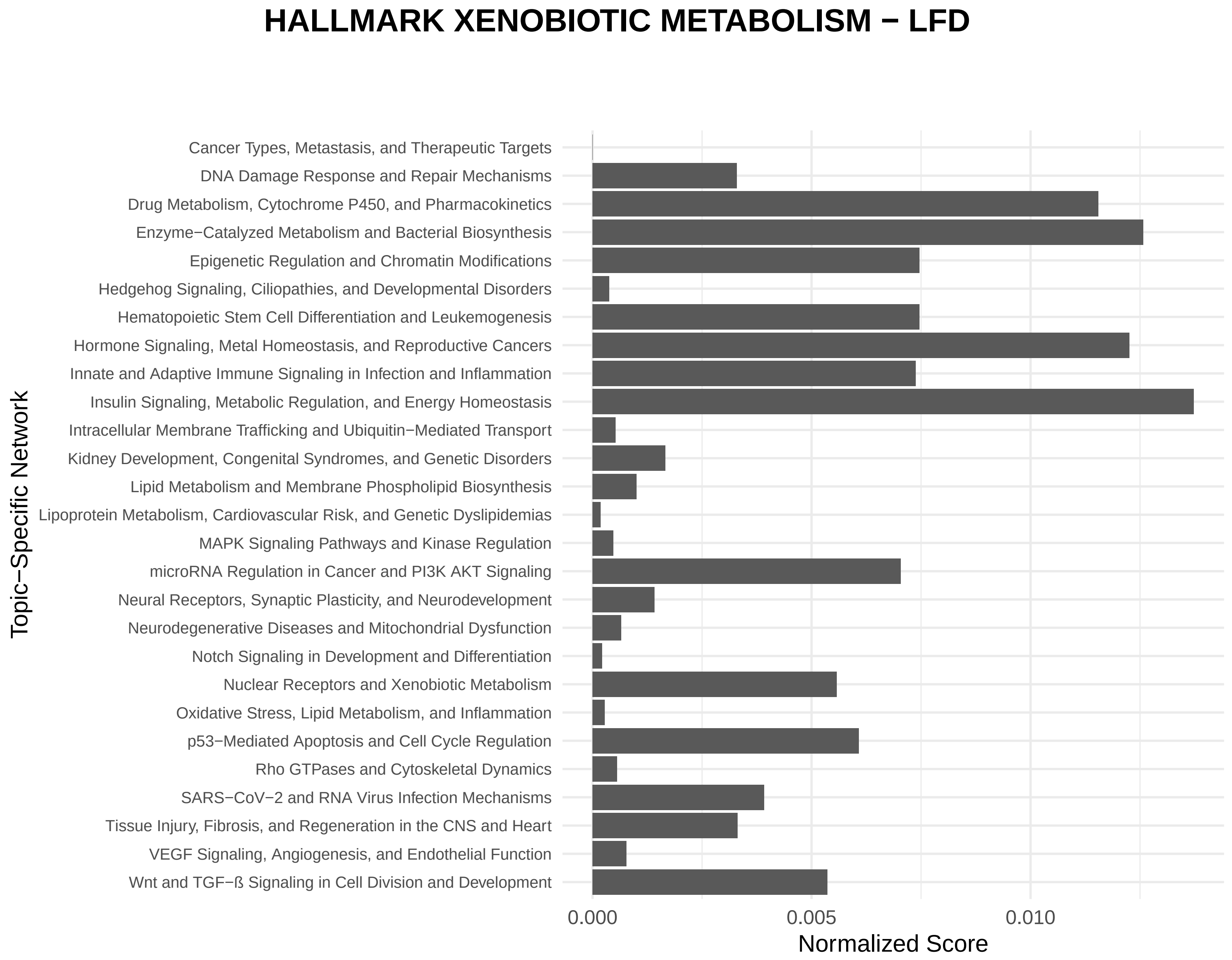
HALLMARK XENOBIOTIC METABOLISM − LFD
Topic−Specific Network
Cancer Types, Metastasis, and Therapeutic Targets
DNA Damage Response and Repair Mechanisms
Drug Metabolism, Cytochrome P450, and Pharmacokinetics
Enzyme−Catalyzed Metabolism and Bacterial Biosynthesis
Epigenetic Regulation and Chromatin Modifications
Hedgehog Signaling, Ciliopathies, and Developmental Disorders
Hematopoietic Stem Cell Differentiation and Leukemogenesis
Hormone Signaling, Metal Homeostasis, and Reproductive Cancers
Innate and Adaptive Immune Signaling in Infection and Inflammation
Insulin Signaling, Metabolic Regulation, and Energy Homeostasis
Intracellular Membrane Trafficking and Ubiquitin−Mediated Transport
Kidney Development, Congenital Syndromes, and Genetic Disorders
Lipid Metabolism and Membrane Phospholipid Biosynthesis
Lipoprotein Metabolism, Cardiovascular Risk, and Genetic Dyslipidemias
MAPK Signaling Pathways and Kinase Regulation
microRNA Regulation in Cancer and PI3K AKT Signaling
Neural Receptors, Synaptic Plasticity, and Neurodevelopment
Neurodegenerative Diseases and Mitochondrial Dysfunction
Notch Signaling in Development and Differentiation
Nuclear Receptors and Xenobiotic Metabolism
Oxidative Stress, Lipid Metabolism, and Inflammation
p53−Mediated Apoptosis and Cell Cycle Regulation
Rho GTPases and Cytoskeletal Dynamics
SARS−CoV−2 and RNA Virus Infection Mechanisms
Tissue Injury, Fibrosis, and Regeneration in the CNS and Heart
VEGF Signaling, Angiogenesis, and Endothelial Function
Wnt and TGF−ß Signaling in Cell Division and Development
0.000
0.005
0.010
Normalized Score

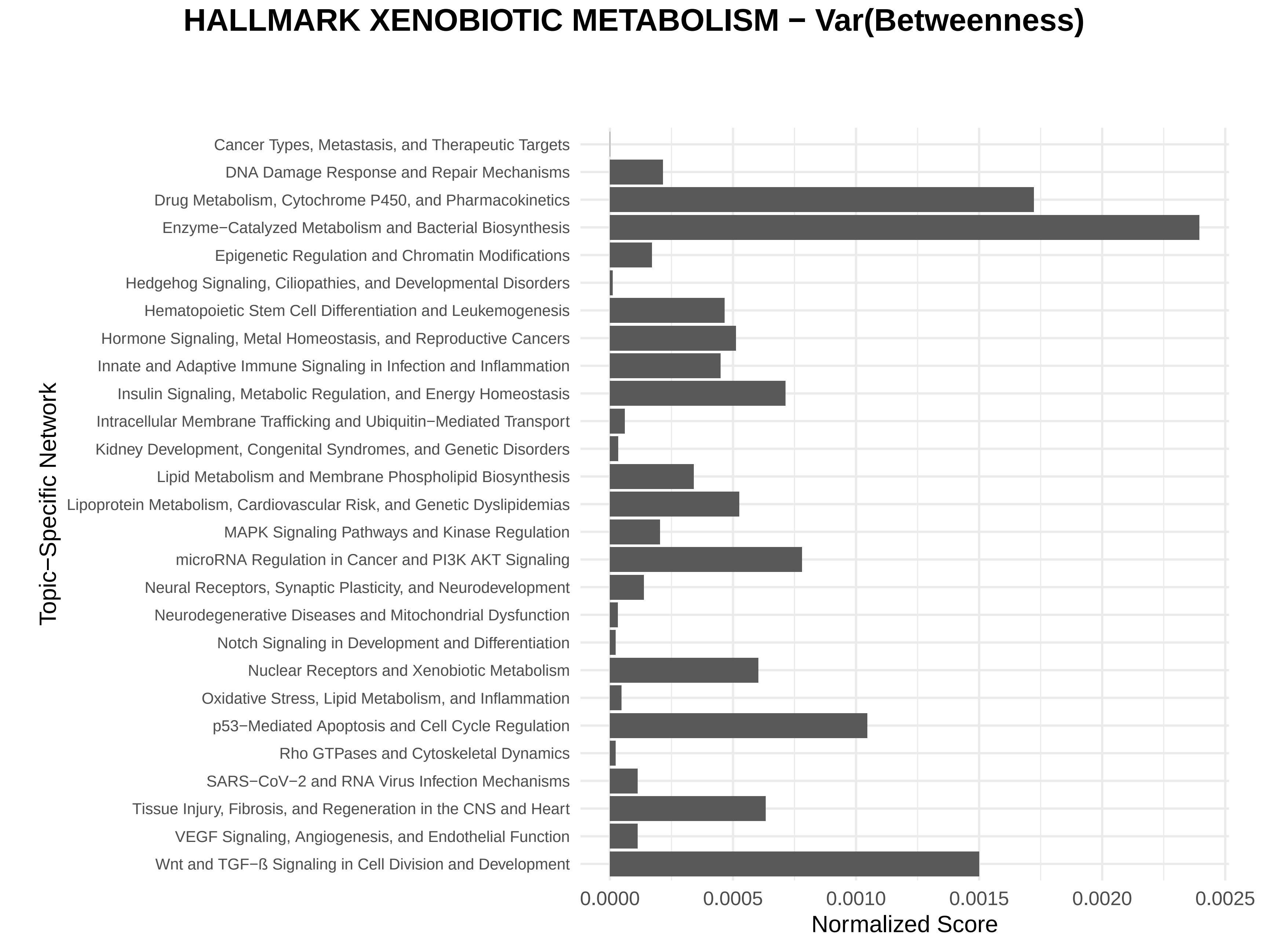
HALLMARK XENOBIOTIC METABOLISM − Var(Betweenness)
Cancer Types, Metastasis, and Therapeutic Targets
DNA Damage Response and Repair Mechanisms
Drug Metabolism, Cytochrome P450, and Pharmacokinetics
Enzyme−Catalyzed Metabolism and Bacterial Biosynthesis
Epigenetic Regulation and Chromatin Modifications
Hedgehog Signaling, Ciliopathies, and Developmental Disorders
Hematopoietic Stem Cell Differentiation and Leukemogenesis
Hormone Signaling, Metal Homeostasis, and Reproductive Cancers
Innate and Adaptive Immune Signaling in Infection and Inflammation
Insulin Signaling, Metabolic Regulation, and Energy Homeostasis
Intracellular Membrane Trafficking and Ubiquitin−Mediated Transport
Kidney Development, Congenital Syndromes, and Genetic Disorders
Lipid Metabolism and Membrane Phospholipid Biosynthesis
Lipoprotein Metabolism, Cardiovascular Risk, and Genetic Dyslipidemias
MAPK Signaling Pathways and Kinase Regulation
microRNA Regulation in Cancer and PI3K AKT Signaling
Neural Receptors, Synaptic Plasticity, and Neurodevelopment
Neurodegenerative Diseases and Mitochondrial Dysfunction
Notch Signaling in Development and Differentiation
Nuclear Receptors and Xenobiotic Metabolism
Oxidative Stress, Lipid Metabolism, and Inflammation
p53−Mediated Apoptosis and Cell Cycle Regulation
Rho GTPases and Cytoskeletal Dynamics
SARS−CoV−2 and RNA Virus Infection Mechanisms
Tissue Injury, Fibrosis, and Regeneration in the CNS and Heart
VEGF Signaling, Angiogenesis, and Endothelial Function
Wnt and TGF−ß Signaling in Cell Division and Development
Topic−Specific Network
0.0000
0.0005
0.0010
0.0015
0.0020
0.0025
Normalized Score
