# Supplementary material for: CENTRA: knowledge-based gene contextuality graphs reveal functional master regulators by centrality and fractality
Source: NAR Genom Bioinform. 2025 Dec 19;7(4):lqaf196. doi: 10.1093/nargab/lqaf196 (PMC12714692; doi:10.1093/nargab/lqaf196)
Supplement: lqaf196_Supplemental_Files [file lqaf196_supplemental_files.zip › Supplementary File S1 PubMed IDs of included Abstracts.pdf]

14576184	16121031	15033539	16116475	17252012
20584986	15657362	12137940	17234778	17450142
17001320	15688035	17952126	12414654	18948947
12471561	17483311	14525773	19029983	15674340
12197474	33428749	15711547	18710942	12900543
15388584	12642603	18849966	18794116	16551631
20085707	18443585	18454173	18172325	16491124
18413731	17213802	19061838	17234794	17119049
18838534	17353267	15735755	18199539	17234770
15252187	18757432	16501609	16877703	17548472
16611997	18490921	18316590	18272964	15681441
15592509	16651414	12379459	16314830	14604967
15199222	17234781	18710938	10737792	11830491
17486063	15070671	16990782	15592499	18701473
17043641	18838536	16909116	16983338	17636019
17287851	16397028	16314847	18849962	16652140
15831697	18339861	15856024	34197623	18757399
17621274	17562867	18212061	17344918	20406972
20123981	16617321	18519664	18506891	17072329
17409456	16273092	17430594	17704800	17525748
16116230	14769913	17242180	17213801	16862181
18381438	32416070	10756030	18794137	18172260
17322878	17409441	18676852	14676830	11741835
15824734	18660816	16288205	12791645	16007187
22208948	16341039	17404577	17043659	14522256
21320499	17195838	12480690	17409444	16565084
15756019	18931683	17146443	16207825	18381450
17213814	18593951	21364935	15608684	16434974
11384963	15635089	16424048	18381411	21873988
16954472	16862182	18339859	17709385	15817677
12393420	15674348	12915101	14519204	17724461
15303102	19414752	17242199	17525749	20624283
18264110	20336062	16107850	15095275	19832978
16331256	20956565	15757903	15735721	15897907
17438134	17016442	18344982	16849537	12695333
12479369	17187432	14573703	12947006	12086890
15042541	18413726	12824457	17260020	12637319
15520196	17483317	18923524	12771951	15958647
19010895	12447701	14981515	12885910	17952124
17934481	18849970	12704389	17072333	17785439
17353275	16799620	15711545	19884340	20060365
16331246	20421412	18701503	15870273	18438415
17704777	12969979	16607279	11423116	18245477
15980968	11711622	16160012	17452451	18794900
15806164	16769770	14684422	17621275	27280975
16091735	17308078	15336447	19189975	15307835
17043639	18195040	15452378	17546029	25721503
17072343	18288184	20385362	9861020	16627760
15778709	14701746	17001317	28498607	17968324
15331443	18157142	17409404	17713554	28119430
14707115	20087356	16909099	18339860	20421419

20713442	16607286	12673201	18724357	15940259
17538624	18317448	20385360	17178841	17623797
16849555	17726467	17170726	18451147	18026104
14973112	17173069	12411303	12228721	18762803
16849584	17210682	20655465	18212054	15033468
18427549	17160024	11779835	17086209	15213097
18711403	17717066	20124474	18281487	18227149
18316615	11991981	11468180	16288221	14499499
18574468	16007192	12411319	14508521	18757430
18710934	17210724	15295046	18519682	16116477
16785517	15310760	29346386	16210406	17060456
15735688	20609354	16585179	11137995	16885346
16532023	17440070	18724378	17289871	17682065
20308323	17704799	16998498	16715129	17430890
18591254	15897887	16474848	15578091	20584988
12198119	28446439	18413733	12096084	17114343
16105978	18037961	17088532	15592526	12531789
17998939	20385766	14512514	14562044	16205638
12824169	12791650	18923165	19047146	17145885
18632613	19596788	19723656	17928865	16186804
16892093	17252022	32663312	11172053	19364821
17039238	17483333	16612386	17922013	16247484
18591936	16044152	16278680	12692265	18955504
16912112	15331438	17009164	17440068	16908595
17878328	16547494	11773596	28855211	16462763
18632645	16914566	17332353	15031206	18488029
17130830	15205324	18593902	17676035	16247469
18381431	16983347	19620289	15386376	16909124
17220874	18381439	17468757	15750623	15870693
16741228	15492243	16760442	17998334	16424014
17960246	12354774	16247483	18246078	18985860
11487589	18337816	18059283	15318170	12036489
12907719	19074870	17409405	17486088	16314831
21177505	11902584	16932341	15103417	19858293
19074876	18542059	15592519	11313881	18701491
12021175	16205646	15120960	20478528	18690214
15985538	15459216	16799640	16293602	18451145
16501597	16407832	33096892	18838539	20211141
19108712	23636981	12663442	14734480	14615372
18701499	12610208	17297441	19805515	15475267
12438227	14625302	15146237	15579653	15288478
17072321	15688027	12648972	11402317	18042934
16899599	16732325	15710396	15592527	17332321
33829040	17483324	19188439	16710476	19289500
15931389	18172295	19564420	16619041	15897889
12433680	14673169	16007138	15769904	14527998
19139271	17099726	12446780	17470548	12606941
17873875	17533367	17873912	19307312	16819509
15548615	16507773	12234922	17891185	12914697
10521349	17237784	11416145	11309484	18676831
18075594	19074878	17101797	15642117	16434967

15897883	18039849	16998502	12213819	16166251
15608679	20538915	16357323	17597811	15940270
17533364	15657340	17363560	12969976	18281475
17369842	12057921	15735737	11532376	18381418
16449969	15531917	18989307	17237808	16799638
10888876	18391978	16551867	16751803	17891175
10464095	18794111	12130493	18413734	16774935
17515606	16540645	17486082	16936776	17873882
15210650	18223687	18339881	11027337	15485929
15565109	19451229	14566342	17142811	17967875
16532004	16007190	18794130	17694077	16741146
15349906	17293870	12540486	15735679	17483347
18245461	15608688	18632628	11493461	12893766
18037878	16568090	18641651	12646203	11781229
17452975	15190254	15869869	16491115	19433444
17151600	20708159	18500333	17210693	17339329
15699330	15591113	27191501	17699763	18066064
15105423	17308118	15731117	18071306	16953217
11279020	10741968	15558012	17464315	15735714
12885785	26763932	18600261	16849564	15738394
15695336	10644742	18288197	16140920	20074520
19635812	19332562	16507782	15674343	19364815
18451154	17189429	16785998	12185249	20479124
17220891	12623842	18285459	12766259	18829567
11861364	17325667	17440110	16772382	17287849
16127159	12091409	18509334	17440165	17563751
11468144	15824735	17603471	16618808	18443592
18504433	19917725	18829530	15339848	12198161
17603561	17409432	18451862	19010930	18644872
20346151	29625051	18519693	17297461	20516219
16532037	15374877	15637593	20399149	18006812
12356685	32573711	18593922	15302897	16533756
12702554	15750621	18212050	15664994	18852285
17242205	15661559	16127434	17213809	17965720
11522623	15601821	20144757	12670911	19752192
18245465	10969808	15515172	17724462	17308109
18815592	17452985	11438693	15558013	11807556
19074894	17322921	17213808	16508012	15864312
17308126	15831586	18413728	16785988	18339882
18332864	17237765	18794119	18426912	16636670
16990345	20159609	17873908	17211412	16531451
16205643	15302890	12808457	17667943	17971902
20123980	18245467	15827134	12855579	17922014
16618720	11755394	14606959	16909110	19103759
11509180	15897246	18922927	18483246	17510386
19289498	17694086	17761758	12802282	20609350
18676865	17934524	18632618	16651410	17363561
11981038	15608685	16832351	17377532	25681390
16369491	18281483	15024077	18339843	11910354
17072341	18559539	16909125	12682234	16670265
18757419	18245484	18724358	16740760	18981216

17353439	15749026	17213807	15271793	19564408
18438429	16331255	15735734	15516973	11279127
12228720	19805517	11016956	18981221	15531915
12469122	17377531	16007217	17597760	18381434
16909112	15198980	18829555	15187151	12198164
11070096	18281472	16467079	17468756	17173050
18490920	14530283	18451135	18625718	12618007
20837710	16832346	16540638	19043405	19158274
16314843	17413002	21844125	17606841	15637585
20547756	15471956	12591738	20038531	17767167
17934472	15856012	12717449	15722553	18039842
15155749	12832290	15824737	16394013	19414603
17283130	15026540	11867723	16799645	17297437
17483325	28847964	16478745	18413723	15189810
20404087	12839967	17906636	17260014	18245496
18701482	18794802	17206142	17173048	16467078
11799067	17404570	17334370	18663361	17954559
16449971	17072322	15548687	17898786	16449976
17330098	17200670	20227041	17483357	15107491
15184677	12640676	11937641	17875932	17452977
18679425	15516975	18469815	18519671	17486081
33263276	17143286	18172294	12418965	15755900
18974135	19273610	18285465	16788156	17392792
18381423	18544741	17443180	16247478	15721472
18316601	12789274	19687298	17440099	15735726
17213805	16707453	18974123	15895078	19703992
17334389	11139609	18676871	17283122	17440082
17406368	18026132	15033914	15608674	17438126
15562319	17982488	17001308	17998335	19047173
21159642	17709396	18378698	19074901	17975138
15741219	33766982	18542061	17389037	16751804
18264134	16158056	17403900	17496919	16247446
14973077	15580292	19047182	16574658	20541704
15226186	17952090	17599052	15273285	16135788
16007141	16407838	20231363	16041372	15635450
16247447	11516994	11992124	16077073	19307311
16702952	17470557	11904358	15051823	17440055
17728400	16912175	15608639	16606835	19580510
18519667	19289501	16116479	15084694	17047040
12732648	11867738	18762810	15561778	16585155
15492259	18505969	16103878	17371845	17255260
17724341	18568025	11982916	15940248	17334365
16527888	16407836	17297473	17923686	16007088
29045844	17283119	16818636	20956564	12419474
18171984	18641660	17438526	11823860	15897868
17334401	19074882	17533371	16079796	18504438
15834410	16150706	12663452	17483315	15297311
18413748	16607285	18185580	17297478	12032322
19074895	19010892	14517214	16109776	18235444
33429950	11786909	17363581	20129251	16006652
17500590	16862184	18772890	12923195	12200464

17234769	18974140	18805970	18197893	21116052
17210681	17016446	16236521	18174920	24847263
17429401	12016162	12563308	16286565	29378302
14737109	18281482	16146838	12649599	25394486
15829979	15592518	12951584	17332888	31191253
12058064	16311603	16642045	19549592	27807401
17452456	17938208	15625120	15590995	19903023
15100412	20805357	11721960	12943727	19464758
15608677	16382050	12468433	11208606	12805290
18779318	17618270	16352814	17309947	15645263
17297452	12381414	12529654	10746853	26074767
18070364	18483247	12032780	12023610	24529521
18806801	17178890	10717473	17445565	26136647
12095419	20584981	19075268	14710779	20101724
17700529	12719586	12130514	8835634	18252769
18632634	18559513	12393465	10571983	15314242
16595783	18536717	11090075	10331992	2793832
17409455	15619625	15160934	10667806	8071227
17908789	14517420	11607818	12216939	8444803
18397753	14639605	19855079	12563298	9811644
17043662	19010928	17554387	17172287	10931327
18199530	15930281	15001769	15141068	10984043
17486072	12393520	10204118	17215350	11447132
18381945	16728703	10959047	16116284	12562791
18381452	11861292	15489912	15670154	14450717
18560354	17237761	11171368	14522973	15134748
16951684	18948956	11984870	15232608	15489439
16116430	18264109	12640114	15750290	15695810
12138103	12679051	14623871	18675468	15809294
18724390	17043644	11703922	9848086	16237198
11439330	18757440	12665527	18035450	16277754
17456585	17404513	10890911	25450339	16452451
33458693	18025224	15551862	29890840	16995900
17099727	18754011	15561584	25767489	17085508
16424387	12673210	15733744	11484003	17190829
15845533	9826724	12829246	15177383	17827295
16140955	17074815	15980064	17568632	18199744
12738660	15897880	10766253	22842534	18304669
17283147	21596316	15856064	21663966	11479276
16652150	22159717	12754525	16677790	28700839
19150350	9799792	11557972	14557245	28512398
18316609	18424520	14716305	24051203	30879475
18381416	18375759	9671776	30741722	30870681
12604609	10557362	10905472	10527810	23415570
15897875	11731493	12368907	31655116	16924260
17551146	16135226	11479627	18648507	16723044
19451226	11929526	15058305	20332425	28669745
16261164	18326572	14679176	16394273	32344665
16007176	16109926	9687539	11551913	16372325
12554760	17873039	16002531	28057414	16603792
18754008	17214741	11850323	27994051	17051205

16909017	23803157	16124859	14556242	18086859
14569202	15654015	17222174	11736900	12838335
15310460	15725726	15564123	16054233	11413485
12595144	15322527	10517845	11130178	15111104
17434459	12838338	16595667	28286127	11299192
27649160	19148180	17462988	25766766	11698232
19230774	22429822	14996803	16858867	8125957
16026864	25526085	12644495	12516863	15000521
11239414	15601572	11172168	19308322	9149541
11870681	9894605	11477111	10838567	10997573
16713195	23070002	10719286	17893748	9235901
30837838	18304445	17976144	17337257	7861177
29626651	20533903	11686879	21665970	14572802
26264610	15719025	17133123	11436317	10331643
28148298	24278747	18274560	16564093	11080247
28878620	21145844	18274559	12482854	10508738
17409386	18023214	12093011	12087109	12972622
17566607	16397578	17364144	11500517	14517314
26388731	21914490	10811837	16766224	21338471
25991442	22465226	15049952	17719544	22129055
32119873	26231733	15762980	24184513	22002090
28057298	22075987	9534029	20018209	19172794
24619348	18238895	11913070	22158834	19475418
27815720	12949498	12192541	28784662	19434403
29476642	14737178	11396701	20937905	26228554
21722302	9916983	17192582	21959040	20807542
16472115	11520933	15132715	19826053	17988210
18519638	24769728	11577986	22562166	11805843
21834058	18208375	14682358	11734571	16799263
31319884	19770840	15630019	17158541	18981594
29605155	20075940	12677003	16474624	16552170
25071440	14636671	15818466	16110317	10747208
29299811	15292194	15363605	11103787	16701909
30242016	15205388	11891122	12850530	16096350
23361386	14660610	11687826	17149381	12763850
15771591	14626496	12471249	15746962	11294886
15814328	14622984	15960985	15551095	15736582
11905820	11679404	15084255	12461567	14659803
15304636	11328810	25374504	10358768	19403607
16200082	10908295	17169551	15886119	28596175
12776207	9812980	26651291	15752555	14690046
12974477	5288244	15948971	12033741	11910893
15224093	16499623	12588368	15364057	9597126
16181333	15590624	15978322	15771577	16034094
15653312	19205777	15468170	15886112	12234363
11244040	17413274	16881963	15457440	15071551
15745856	12270949	16306523	15322145	14519386
15224094	11716909	11276000	15626471	10798271
15653315	16823481	15270885	12119152	9150551
20010787	18382419	14737121	9697839	12810109
26615431	16101641	15656799	11163197	14519398

15882774	15771581	25568220	19321146	9417034
11050419	17496928	26082779	19567913	17636923
11860281	9765254	21811895	19665965	11572082
10964748	15046611	25705421	16286919	12921709
12367625	12051620	23110467	19767723	12125070
10664064	17675482	17635716	23258413	12796473
14556710	14607270	26324800	35006429	7698997
15115723	15719031	23911436	34949589	10617638
15056568	11071626	24924409	32829024	15036264
15728677	14982876	20027185	34970269	15381249
12500939	10403855	23564577	34504660	11331578
15923648	15156182	23564578	18672201	12736199
7908906	16484590	25309880	19273156	15229478
8242749	9834202	NA	11805847	12845610
16936746	12801837	15102328	19017683	29079659
15838516	11160144	14551435	19246813	19007773
17568790	12244301	12022921	14522402	18647649
9832503	22286129	12670965	15146239	15261671
19020303	27990019	17169919	11524405	19325624
10385618	32226546	17030798	16817756	20110990
12809600	12015613	18625006	8087839	15687999
12925736	12397359	18391471	9759502	19924646
16212951	12724733	10613873	16038189	16203612
15838523	12507418	12644499	18043895	19696797
11099028	18802415	18520135	16550168	22193161
7973727	15000146	19270684	16393888	15947972
9889196	16555243	24892271	11226815	14747944
17018296	11078609	21106488	10575324	16508724
9029154	16699851	27577879	9698084	17028294
15876871	12621137	15500860	11504799	11431710
17426725	12951588	16034365	10901707	15284264
12573239	10505543	23847622	12399158	7585656
15568976	16042571	21349428	18463198	16248975
9618481	15349822	17530315	12386121	14700549
26650195	9196022	24381786	10859160	15694859
20935501	12737309	18926762	15788214	11389469
12495636	11459867	21155715	16048561	12197905
11872087	15573119	22410872	NA	14967450
12069927	15310786	12189384	15514566	11252954
17169327	11223406	26115564	10976516	16829981
11244042	15711891	23612460	15173095	17000658
8576262	11170304	22710174	11489791	16377102
17537990	16326109	19362700	11379777	14744244
18662984	15479695	19497760	24281184	12851486
10852130	11477132	18573338	27709007	15864276
14625285	19167459	19122387	27232112	16729045
17371236	18568040	18280611	20195289	16729043
16144840	24213116	18941233	20434429	10880430
18711432	20554751	19590578	26867495	9872057
19159344	26149458	18375758	22846601	15863494
15803148	26489954	18724932	12069966	10490623

9642287	18092812	1512203	11102867	29290584
11275267	12571226	10092508	8253761	24582806
11015622	21867484	10542045	14517336	27779865
15788404	12893295	12431977	10329735	15771582
869535	1902669	14663079	14734546	12415312
12360215	14506308	15522832	12802054	15156179
14630197	15033580	1649829	12807000	14985712
10586892	9535909	10938271	15623507	12360214
16470226	10751659	11823218	15635094	9927518
11079101	10873290	10473374	24364251	11905810
11449275	12566218	17636255	25509331	15738953
12145654	15094344	16707670	19837036	14670296
12488490	15955302	15470257	26563290	15629410
11708889	15033579	7639721	33112703	18086882
15466916	15033578	11341914	32156170	17571595
11244038	15639402	15520012	10978349	18430459
18322649	12154354	11042448	18065535	15902993
10376013	11253051	11274177	18819910	12589470
10577498	16700615	11099377	9596633	16935872
10224668	12762887	10473568	16407450	17395553
11228151	16095998	11551958	21252347	15459747
17050193	11057895	9506957	11513957	17041811
17308335	9643506	12512856	15350605	18951640
11304556	12084351	17253960	15613448	16288298
15485887	12675513	12716890	15982836	15336977
9545263	18482986	21138417	10707049	14526190
12821647	14583094	22899863	14596811	12372277
9417101	11157967	17928217	12943720	15242639
10601359	15610825	14568616	15080147	24739785
10755615	12049631	19661164	12963732	19525941
10586078	9677355	17239763	18708085	26920688
9581795	12750367	12847091	16412784	25191533
14756624	12967709	11390981	14735553	15695335
15179030	12121990	10318803	17900507	29427371
11573199	15537651	9756849	15882431	11807533
15459662	7813456	10742590	12359826	17081970
11229600	12751784	9151776	14556773	30941017
12235283	18395526	9988768	16147539	15996541
12640026	8892297	10520990	15372473	11738471
15688067	11150302	15304505	17784964	15996546
14519391	12949079	16099108	26875496	11839795
15108811	9851611	11030741	23719536	25270767
14744429	12446213	14706853	26077918	16522639
11082269	10713147	12511560	26064136	12844200
10698680	17922848	3161730	23799571	14711353
11882383	12466271	12716912	20708941	10615904
15053919	14982633	10837462	22534646	11371514
11146675	6798961	10748143	18583599	18056765
15034601	8662716	10854428	24925320	19136482
19675644	8995384	10854427	22056910	11225594
16632608	11294844	12655644	26366162	18572189

14993306	15721837	15987739	15319346	16939974
18310071	17543969	18635948	15987776	16856873
14635525	15983048	34249946	9107551	15499580
11282018	15322192	34646033	4395686	10812966
12867054	18070909	11749383	15298956	10764727
10196204	15039466	12191611	16612326	10985348
9748166	10586030	12947395	18157157	13475371
11742412	15831826	12676795	11920679	16894175
15624019	11298294	11369511	11436301	11514508
16288289	12438359	12390245	27304501	16321959
10567225	9504340	14597384	24556838	5835946
9445476	18793215	12566928	25242279	21953451
9609110	10462516	12849693	26269525	14702404
9609111	15639739	20602996	26185979	17408485
15229476	16848789	22367796	26658722	11004450
9346240	11728310	23085193	24698685	15528644
17344645	10608501	22296764	26046644	11921398
18291668	14762510	19483709	26700098	18021072
11343120	14976004	23664135	24842496	19247843
12791379	15604886	15298336	23465396	22221911
12511876	14977592	15604203	23421405	26610024
19079156	15629883	15028942	23694989	26610025
15971106	15173832	14614204	19812304	23124073
18549797	15265790	15383650	16756488	16493424
18243016	10975856	10841026	27027448	24718315
17182536	12540948	15016298	28283069	24707857
18838301	14757320	11023702	15607802	25526305
19079167	12909723	12894244	16109839	19767725
17161619	12324235	16822996	15567854	20643110
18483500	11302930	12753399	12205470	26655628
22383755	9694817	9263329	11062493	27291964
22339650	1634517	10643996	15607806	25879280
21372217	12226220	10347209	11895890	26549800
25217450	8765137	11602344	12530970	23846113
21034406	12415297	11041354	16177799	22430786
25587654	11427694	16246839	8942070	24840700
24069565	9735948	16750612	16002670	23702978
16204702	9602501	15841168	9143705	25145756
23735217	14509570	16001050	14710949	23215645
24606160	15652868	16001072	11905813	25330206
10851053	10652548	16899407	11836514	25879288
11114747	11378299	15009714	12766758	27012466
8682149	15803158	11224709	12752668	26121197
30267440	14715912	15721476	15618404	26482951
34492272	15857686	14695152	9862693	22819539
32203403	9697848	15557758	11390470	21575908
11890722	11802770	10843728	11399424	29148036
10770277	1682209	12487150	16699811	25816776
7591091	12628346	10725110	14704852	16971555
10417174	1883197	10570027	11529497	17589524
7916951	2943218	10490505	10851172	16895466

15578053	15987956	12459728	29477730	11741871
12215643	16878994	11407945	25588886	15697206
17506672	20145624	12946833	26104206	10572115
12379883	24733839	16702400	21488979	10913097
12741677	19652915	10740269	23927593	15901685
16870043	11557990	12648469	27606286	12700258
15948711	16269360	14586404	27617233	16788179
11902573	10944513	12545153	27732847	16428816
10944551	22875451	14580692	20300064	23279921
12867060	12488531	12079267	15318166	16458304
16112428	22735517	15774796	10647931	20023024
12407699	19941039	15823750	16557281	17055078
15942304	19306880	11707511	16551846	17055079
11895903	16760654	23340292	16946003	17010456
16189154	15182704	16495942	17062879	16631601
17625570	15136768	12938733	16242838	10537203
18593892	17480229	26501339	16189702	9603882
19629074	15182701	15767499	11165748	9098055
23201355	16518630	16972273	12094241	19016485
22154278	18631137	18047737	16339096	20660314
21855164	18598214	29090092	15122209	19580826
21753699	18550795	12814656	14634372	19439500
22736493	18946022	20495568	14685170	9461500
21850578	16554437	24781149	15611513	10217486
23733083	18096427	28468938	17211469	10518530
30941048	18311174	28838811	17287871	11007789
29128428	14691134	19663908	12351585	12196148
32326537	16469879	23352769	12878745	12195810
15288257	16584177	27103567	15724144	12055304
15300010	10580130	26297973	16551847	11283349
15288258	11821425	31100053	11900250	17506685
15288260	12626393	24548101	15082523	16245042
15585829	10350056	31587020	9422516	23042036
12464310	15466199	15189334	10835690	23922391
15272268	10207017	26487845	10453277	15828220
11896183	25727146	27546335	11750244	15828221
12417415	11439191	27581196	8069858	15828222
12015972	16557269	27829983	11527574	15828223
10028969	11747320	21674642	12711111	16476485
12681948	11313928	24522549	11720739	16601267
11436314	15116721	25761946	12616528	14999402
11436316	12505356	22926193	11807782	10529898
11436323	10714958	20736035	16064138	15673477
17276014	16697662	25611507	11463388	16511590
17884153	16915296	27911343	15109562	16360030
16922398	17409411	28823929	11559746	11921433
18018633	26037915	29147906	11905738	12543708
9111134	19584092	31054074	12243753	17346171
12200473	19240372	30370500	21193867	10198776
12684507	12135761	30937538	24508914	18081658
11278778	26646590	29720658	17251915	18488143

11067870	12045215	12700767	11856310	9763449
16299351	12084906	14657486	12867427	10213783
9292932	11730319	11082272	16042598	9565594
16261175	11063930	10574699	10476961	9553086
11941303	10497333	15325584	10476960	10459012
17960151	12374568	16099218	10497122	12893879
17952897	12477927	16200202	10937989	10085287
18093537	11148128	15325588	10937990	10397773
14699405	21747944	15928708	11014182	11445562
11058677	20478527	15978794	11014183	12853481
15661024	19399032	11099404	15208624	9446565
14647478	25915839	16125113	18272355	10359592
27055367	25485532	19653858	19120475	9614185
25584786	18172298	19802558	19539500	11208059
20889378	28968951	20034776	18549796	10379359
27060771	16338359	21187343	19615405	11029060
32565065	18497808	21801009	19120476	10788491
18245809	16919475	24343578	18591413	8621431
15695097	16181324	25948417	19076341	9647643
17051207	19483713	18358634	18439848	9427746
11592845	23495936	25048860	18984593	9843576
17085779	28583440	11313942	19224920	12600315
14705136	29071527	19422821	32668226	10097106
10398674	22215586	17229979	19239894	9214619
15890824	10087917	15549172	16968219	9647644
27581199	10652088	15549171	16939780	9452464
31618844	10747959	15833268	17471261	12669082
31795242	11007775	15109615	17447862	10873817
30710214	18046402	16555051	16766188	10982406
23439666	17368028	12793984	17560162	9817754
28643372	16212506	12419208	10499798	10564279
29194372	12475201	16286563	10784442	9707576
18420416	10924740	16581295	19880312	11739776
16844381	17259602	12505055	18573085	10557242
21980554	19299134	12372844	31835031	11251103
25766616	15063851	12019279	21190829	9305917
17466621	19948674	15173164	19051051	9710615
10195903	16914536	17362841	20383543	9813082
14532116	9550618	25332897	17451884	10359608
31417367	16352913	24273531	11782468	11208143
29261664	17557941	21951628	23070004	9852078
21196165	19721811	18793332	16882042	10683148
23479001	12138151	23692861	10839363	9507000
28507517	11826292	24760442	11737951	9628864
15528202	11385088	16968212	11115874	10036234
15640354	11045400	17618621	15342965	9199167
20205843	18056784	11093772	10872468	11278762
29998397	11533299	17893666	11306253	11555414
28714865	10601204	16688755	11104523	9614193
15342004	10986234	16428379	11237004	11035026
15611724	11152757	12136096	11252968	11323436

12068098	23959882	20134025	11171088	18431594
10397763	23467090	21283798	15122199	18566824
12011104	19284629	21781017	15928679	10073616
10652340	25820328	22896795	15084594	10070156
11305904	17287577	22928510	14656980	10099706
1495423	22570381	24548561	11905831	18655910
8457204	14715493	24651541	15286727	11773613
10366774	12471498	25152334	15343367	11001488
19239890	11082195	25469537	15099566	15522987
10978320	12358600	25483301	12669022	17956998
17537823	15073291	25757376	15489916	14586738
19733268	15340795	22892070	12133805	13678960
16531502	15184572	12042308	14647476	11741095
12777052	15883781	18094055	15308211	15602010
10998348	4297098	23885123	15345216	16104843
11469811	16262699	17275323	14617194	14641020
12604236	9858571	17457343	19132916	15338053
12832414	24375100	16932750	12195013	14724572
15642792	14534577	17395581	19290929	12391145
16413106	11252892	16497588	17395537	10942706
16547389	14557817	14698224	16799472	11032799
17459698	14769828	12734364	17187070	11292584
17475203	11252746	14751764	16922370	12102686
17926129	12163231	17637696	16922408	12403808
22982583	11836504	17507094	16922367	13679513
22633058	12466190	11101877	16816840	11953326
20143161	10021351	16924467	16405856	11207565
22092713	9847239	15749753	12944364	11349009
21833341	15063168	15823385	17588513	12671039
16452431	10519551	11078440	15292141	14659695
9695921	19758997	11272200	17458653	15204437
18929068	8093006	15759102	12689814	17156122
18305268	10934147	12480546	16339747	17464284
12835292	16642010	11390407	12693609	11454785
17943287	26735394	9011569	16870827	18039131
8048228	26160612	7491105	19120701	12920582
16932712	15975920	15258147	11165670	17200452
15459673	15671031	9974390	17158339	12439638
15972354	33925597	11325516	17200683	16845885
10430616	20495575	15585596	15269340	16413486
12965173	27353478	9719467	11773611	10904843
10025913	22169974	11507694	18040024	18357777
15380523	17525755	8072542	15026027	18545064
24607224	15820555	8786033	10221985	19357408
24385952	16650994	12796471	10911373	10051305
23261954	16771626	15889095	18552454	14635791
21481196	17182900	15889096	18703404	14745971
23587289	17950242	14617043	11709400	12446114
20696704	18005931	8550403	15725705	14530294
19439032	18946474	1194238	9458738	15102436
17108952	20094051	18435614	12060106	12967557

11433294	10068641	18458083	7927516	18426796
23256519	17368312	18287005	1702903	14742429
19482104	14634621	15485873	16181335	16687408
18838381	9390557	18772128	20171863	14630918
23151663	14679297	18094042	17157489	20022965
28522145	15502848	8327893	19218463	16931790
25808920	15210742	7641683	11742988	18840095
32888647	19817485	8997178	16413484	18048764
28242765	17562450	14514350	12049731	25821458
18832364	19941817	10449730	16982218	18038217
16291214	21642957	17900700	11163244	34611326
20103563	19276888	10903473	12209154	16391004
17709369	20933024	16098514	4561027	12704645
21039332	21543634	29053970	15218528	10205060
11048892	21757760	25561175	15723341	12440953
21352852	17283063	30844724	9422506	22094468
2825027	10934204	26378229	17599937	21347367
9450929	15657177	25567907	16107722	15231740
18691557	10777492	28066558	17961083	20603001
19481063	22986504	27648300	15924268	16756494
19126755	12192414	29236692	19448080	18160255
12161432	15691767	17470430	15901243	11809809
10879540	16459297	28686218	30056832	15592430
18237417	22981989	24529380	15920196	17698587
18043734	10966113	11724780	27558536	26393581
20418862	14614827	21427764	23686171	11034610
18296446	23079593	5661997	17514195	20708099
15965470	19906846	24469809	11160393	26545300
9819428	16339192	23348840	12008020	28630109
18077452	20395968	23530209	1350383	11429545
15652348	21356043	23351793	9495808	23931059
19567869	15564374	21816947	26045554	26339618
19524514	9069263	25047614	17997397	26772212
23064382	592399	13978319	7903167	22030238
24465591	641056	8918697	28303017	23000944
19671665	9732867	10648173	8139656	25060581
17991742	11779870	18187424	11823786	16603771
11751416	11323433	8306975	24003239	23667505
10486198	11744733	1081164	23795651	19074509
18066052	1998122	8163536	26139588	20859064
18816852	10497269	1376928	17716973	19149539
25560970	20525283	9468499	25689043	17572487
18412118	16698963	16849466	23187890	10519914
18394579	19416660	19065780	16762929	9646295
21497760	19075029	18596693	33298899	8387282
10662633	15152008	11606584	16549788	14706825
10668974	19741254	15489917	22751019	3317417
24529385	19737925	7929028	17230199	12941277
20435029	9487141	18818202	19379695	22013193
23973942	20705608	6335036	12724779	21878990
19575673	18195019	1550550	16495441	20533886

12819131	15837415	17221863	17533373	22547309
12941276	23746838	15146185	11238451	21312325
23482940	9282108	21924235	18228324	21478859
16382132	18555778	38372588	7629134	20858899
22326026	13905658	24191001	1396589	21799911
9362062	19597488	16760263	10454565	22363759
20399632	19818705	11136719	11907280	16446366
22389062	22048312	18977199	19362144	14684739
15998802	14615387	19608861	30354722	11970895
17574030	11282294	17356064	18839291	22826439
20145001	8578462	20703077	24179613	19494126
15314642	15118080	17525332	20522543	22986494
23567335	15664996	12086618	22382365	8034666
12169685	22653444	20622854	23706667	2344612
11384985	23945166	21498573	21533068	23212477
12620239	25040720	18367646	9878398	2188731
19851329	17955020	21478295	16169847	21413788
12620240	18786386	18079394	14744247	2188730
12011067	18775307	19962665	17254765	22134922
12598906	16746585	20009532	15557120	19410545
17254298	34884868	18317450	8861902	19660450
15788703	33321098	16339315	15494733	20080577
20816209	15987702	17363375	17395582	17339380
12670394	27049945	23637185	16214811	19410539
10849438	30844107	16702223	18989317	19629040
30621321	6773467	23873287	12408825	22196163
28350208	18926908	10469566	15525529	21453242
31113848	17145306	24119662	18632669	21473702
28029388	8717519	25364732	10898789	19061483
28571533	12495737	23732476	16510874	22286106
29858187	16280322	19854180	18573912	20064600
23406282	16717127	23378591	11080158	23268465
9566895	20554521	8001155	12408824	21375475
10712901	10430017	22130221	14749774	16325574
10339564	25062251	10562278	16141059	22201797
11046155	15007061	23783758	15475613	12944365
10995389	28146471	10825291	18608124	19243136
10801458	26872272	9288971	17052452	20418328
7641697	9736607	11044372	19521399	20074029
15304631	15339664	25303525	23606632	15571815
19339544	26042225	16799259	2738071	18234723
19704417	8953040	21438114	2775660	19619488
20728359	19092055	21711675	2713493	15459103
20965423	1177317	27245231	2592373	10647780
29078414	27092250	22664329	3009023	19759537
24732012	21445324	21679683	22183981	10581160
23216249	26829388	27974214	16151858	17318175
NA	12719478	10374692	15687324	10421629
28797581	17113138	12876557	21907012	11336703
20188139	16604071	16598076	16846473	25840006
28546457	19696148	24705298	9587028	23730680

12756558	1976634	11336673	12511868	23561633
20414203	12239347	15469835	21851428	23038248
31374202	21376233	15848799	26048987	11080164
22237395	25081058	15093606	23325218	15621527
26708048	17210635	17300218	18082604	16901784
31048490	10535941	20937854	25099582	17158953
25493225	11427533	20026667	17768402	8876179
28572265	11389444	17707230	21734703	22902626
17887919	17591695	23150253	16289108	25435140
26746812	19135894	23153826	12496284	22466610
27932076	21095583	23063748	12565875	32152397
31116084	16751101	18631128	12629551	17290000
29117491	22045334	15491402	19758793	12660172
22983739	11278251	10747205	18606873	20951344
21384229	11158580	8978277	17101212	22617422
2301971	14718519	25887776	11461910	11713476
22838182	20937913	8677749	17646408	11136974
22165913	10757800	11443118	15546612	10831834
21388382	20061380	19616933	17909001	11940650
14742321	16027725	20347046	17122878	1361170
10747039	22344298	15958495	19909365	15458387
15964013	9739761	22101327	16597617	12048180
22055184	11904382	15837422	12791257	16885393
12486230	24402227	18614053	12141425	24452601
8434121	16246722	18377699	9858549	25843800
15790808	19023283	20148673	10982855	18787071
9357549	23912815	28539435	15863030	18444242
15574587	27312108	25303530	17505008	16493026
8242752	24699078	25597633	18757403	21159781
9843217	19754430	17510434	11325814	15084259
10669743	26085183	23850489	9154000	11964479
2342578	23732108	10873660	20101236	11094066
19528227	23657496	19274700	19103595	23580232
8242751	19489725	23954741	11003564	21196578
16166375	19436320	19339991	9538690	23918930
2392154	23624913	27288742	18381441	14966295
17694070	21307119	17037979	11056689	24047697
22781841	11283727	21680538	17552943	26780985
7079182	15021893	17567461	9266968	21164521
24214338	26864683	10441588	21080029	17667983
23687512	26423937	3893466	18406375	26715362
24589714	10727776	18110463	20032467	11777915
16403636	26054742	2729894	11012776	28890329
23938249	15001665	224930	19799112	16943287
21243710	7938166	21917992	18698078	23707940
23587238	17450176	15496458	19276253	10102273
19657014	19345195	15956359	17475912	22412893
23166392	17613433	14534365	7692230	20978166
24059801	12482742	17916740	30552988	14576824
1536007	11208594	29980625	17668209	19276113
7623835	10551886	21354435	8104191	29533771

25425107	9662327	16533948	24141787	10684278
27730540	8050359	9458043	17996710	9188632
15824087	9020173	16102042	11376695	7474100
15866051	14990995	7997267	11242102	7474080
14701673	10228158	12005431	18285462	18839070
26700816	20655481	15601831	14976220	20156840
27869526	17364682	17488973	10930435	19356150
22511764	15269338	8548048	19442142	177353
27547294	20227374	17041586	22216413	11008000
22876196	26131740	25789972	9839614	8605876
16840535	8665925	16012167	15944408	17969444
11779849	8522583	25201414	9716487	8621447
29056338	11737941	25643323	14512302	15607020
26523980	11146276	25642960	12133942	19059436
24291305	9861669	19357644	9885206	18929502
25007762	19412884	20483915	21349098	14525967
10913147	17560368	25642963	23436998	19022706
21389279	19165215	18539610	20890289	18613828
26052839	21755468	9837724	26581522	19302047
12239572	19239886	17962301	14527418	22595692
24167160	25565029	12139939	19457864	20410258
24920680	15741316	12791985	20144995	7632928
10358014	21801010	17928296	18624796	16775004
20921137	14723851	17965729	22252131	16061793
18612045	10075717	21804533	15075377	16049494
24040102	17442700	15485900	11836526	16229832
27731402	2105456	10212258	19900460	11756463
24028821	17000779	21052091	16113677	25343031
21536589	15087129	22977173	11081636	24068461
16783365	9452474	16396909	19432801	21421921
27791111	17306374	8824192	20359876	16157178
17395749	11815463	18585355	17943301	19575639
18827341	9914469	21257310	11331907	25233431
11447289	12475787	22902404	20414202	21436057
16682955	17478680	11395778	27193484	23839779
23493540	16990140	15163793	20646998	23846822
23493538	8825650	23565096	25535373	25708460
26199742	9701566	15075292	25713138	23881164
24666553	21220505	9593755	20676083	15069402
13093749	21382416	21502959	23142665	24316379
18220710	20823909	16987893	28992441	16413926
8213607	21704024	26741528	16777091	19846511
25639270	19703651	29452096	25043001	20213925
5823111	20919643	9759480	10508650	16287098
9778043	29747811	10550206	17084981	18626067
8288595	11408587	20871616	19118207	19851335
10597240	10679020	19268590	28106780	9211984
15014447	18385516	19369211	14754902	17189949
15084262	11739401	23149936	17624957	20519495
7929342	20937827	9880493	8041786	16630821
11988738	11285137	20965415	10620603	8906794

21664576	21599891	19703008	19962410	12526811
16136078	25436572	15364901	24842111	11208076
8906787	21647776	15211354	23149385	9384576
8898207	21647775	17057362	14715917	9811606
22326621	10755665	8252621	15738989	9564030
17641202	22791193	19330724	15886331	19933846
8609415	23620664	23511973	14603310	21613978
17168669	17215883	24388755	34758268	21177493
19339967	15157936	24071720	15111103	7478553
28642194	12827356	28610953	17244524	8626428
23376167	16675045	23732472	9724731	19219045
21921683	16908340	16212495	1828394	12072434
20485518	14530973	29364559	9508775	19115315
22586411	17374385	31894645	9448290	24835590
25732612	19164095	17035353	8001816	23382213
23770565	17439925	29222186	8078588	20805990
12126930	16825786	15102545	1828392	25114211
9893266	12836025	16126164	8246996	17496916
11324705	16720382	19262508	23842645	23443682
21779496	19946895	15744310	10773440	15664191
22569528	10871282	20649548	9190208	9001234
19091303	24518591	22817841	8497257	23208509
22733995	16849525	21629705	7859739	14684163
22800433	12006644	22146081	1828393	12475979
18593949	8703035	19575678	12360190	24828152
21329875	15314156	22265687	20584987	9442023
21372320	9038378	19682549	22500797	9009191
21164013	24951116	19076454	21552327	8625412
16600872	8906793	20533901	15930137	12807427
21164014	18779368	19008463	18760361	14672936
34782609	9070211	18430751	15662836	25740432
34235568	12147692	24366263	15242646	22001063
34495539	11894096	20079335	17301054	11585921
18952054	10521801	25463276	18206966	21402876
26733807	8384622	19021507	17098746	20006983
24150225	11478803	20522547	23290524	21031079
18511691	24814316	20873749	18782863	10383463
23493374	16782899	17996737	16980297	18322270
19628041	15713485	19767391	25314029	12775774
20385708	16537482	19720617	10597293	11357136
22262897	24823486	28765093	12740388	15908424
23259945	23626807	20214493	16735510	11448771
18989361	15301554	28301740	19245792	12832477
26214522	7513258	20100523	10713156	16571882
25424712	20015050	24440477	20029837	15994232
22231481	8208547	24362565	9790970	19649571
25579386	20670691	24911204	23394999	23650620
15951480	11420688	22986124	23001041	1378856
18974038	21664574	25088301	22836579	20338884
1390320	8689559	19716793	11593029	16585540
15583024	9316394	22172991	12414957	16618810

12884866	23362348	10744726	24814062	17956313
18585455	11866432	11679632	24202393	9090381
18414735	11986231	16061658	22589270	27977397
19483673	21898486	15023535	24651010	23798008
22179609	18724371	23230144	22020331	25177298
22373579	23362349	14599765	19451217	24508507
10802669	10958687	10583946	17254968	10767286
23306437	24842903	11900249	11564866	21464226
21293379	19640841	19008000	16337592	29321502
11955432	15533827	1370859	15287722	3693402
23505375	25125655	18938244	17210787	27284042
23727112	25356737	17509148	12189133	22555564
10572166	20804727	27587538	7816143	20385596
22529269	18593473	18043707	9628874	15581572
8422676	16368887	2105931	9162092	12948489
12023295	12386158	19561302	22325352	16378245
10757784	18974129	9590179	12134156	1656221
19001859	11971902	24098485	7588633	10579907
23333306	16365048	24388663	8846784	19644473
11030616	27749937	33447881	9030721	21430697
10959836	27841855	11060288	8774846	10866302
17470781	10837024	25977809	12171911	9326929
25512557	11518718	20200447	9195981	17611497
15451442	24667410	16720576	8622688	18954143
11493912	10964583	16469703	9564042	17604717
19859661	23576639	17973576	8622669	19487573
17531985	18239137	1840504	15866172	9467011
21496649	17948123	7522482	11062067	10555148
20795950	15531924	14983052	19462008	16949365
17352659	12374742	16402120	15563468	17940011
17671086	12897056	12839923	17395589	22221002
12446846	14986688	26105537	10842357	21427766
12842859	16530045	10733595	11067851	23980886
16530044	16921404	9824166	12096136	12558974
15107403	16429119	11883935	22722607	10918587
8687460	11112321	10722742	16764946	12089343
12391150	10487760	14729966	19096775	19196647
8649374	16024779	9529249	17513417	20861159
18374649	11535832	8552081	19096781	20192774
8001118	12370315	15572696	9780000	20043944
18094723	19520913	27812880	12200445	20559318
12050117	20368433	9704925	9214649	17605038
10713164	24060861	22336108	10559261	16828287
16256737	18097461	20583212	19365405	18216768
15569487	29455642	11234019	17433286	18216767
20156974	27573755	7588608	19204114	18726176
10206645	22901806	18365017	21983142	17330069
10551863	21335236	8259215	9598355	15269334
21632553	12589057	8521522	20004982	16125054
18469519	18316408	25907612	23140633	2018971
10934472	16912174	21993244	18848778	8704248

12411949	11896061	18487508	29723737	16503871
1672265	21102611	17681183	25818528	19416966
12681240	17591783	19473982	31277234	10217147
16543958	14563852	21289308	26036474	10358075
9295288	17768054	19884659	32178475	15987244
15561706	12192048	26415504	23850828	11237865
11554294	11031238	16702404	32542921	24105466
1670772	7597053	18172008	11447113	17994015
16574391	23468639	25737250	11821947	26239616
18616426	22231403	24344134	24937142	20673872
16153702	25659039	26279303	2993863	19331822
17183653	11826263	16829519	23993095	22357867
16846370	17696772	17974977	11579234	18566290
20505756	19453276	26561776	15710605	13678582
16819296	26172624	25728608	23954936	22365832
15110706	25833379	15471878	21779505	18615013
24096405	25815272	10913193	18623629	12207013
23473600	26187467	17483331	24671035	23856247
21941108	22708562	15572663	18454133	20223231
19738629	26235645	22084245	8900285	22374676
21248841	24652853	15539407	10811826	21532586
25232742	25175772	20606012	10804209	22778133
15753315	21883762	20554524	24452471	17589498
18474358	24457024	11114724	11575290	26580584
18426888	19775879	23814047	12591178	8700862
20351093	23800009	22231447	18094957	16166372
21490058	19940261	29362479	15457548	15331439
10669757	23061719	28462526	19706539	22759635
9727485	24367090	33033617	23217742	26749280
20805998	25547115	27246931	18547144	21343615
21602795	16418168	14574414	18701067	19114653
19723635	20080666	23303910	21729787	25645944
20080834	11781575	7991565	27819291	22412390
10935637	26280536	21330364	24012004	19858498
20495621	11378386	25402849	20071533	11203699
21551231	11545734	20466531	19258013	8929538
19714243	18757404	22251903	7539918	20139099
19773441	19345329	22694141	9000051	19235904
1764995	26910647	23898197	10202154	25452107
9187150	18327259	16855591	19788888	25562167
8681805	26183061	20937701	9840812	20206228
19701457	18199536	8164745	9632793	26716895
20170518	26862215	27790713	18187047	16751105
8293978	19440552	28202288	14668441	21931630
7918097	12297295	32460002	11959977	26778333
9653148	25867063	28055293	10526574	23403054
20674646	23013792	32362820	16710296	15703179
16738315	11035045	29330095	11751371	25260729
16253240	25884169	25239922	12466191	18541707
16738331	22427670	25309333	17047378	22422618
20462400	23073177	31027363	18288277	12000792

16901655	21317932	26526991	18086875	9535855
11857736	14702041	16246168	24607366	11278486
16728642	12914926	27313038	24388711	16204230
10215629	10435622	7711290	25354898	10470034
10911365	19293287	15277680	31956375	22340718
10572046	21814038	20435355	27707839	21098037
18767071	16213212	18632300	33195688	19513348
18850007	17499002	21377628	33767988	18006505
26004068	11923872	23765164	20405038	10924361
23333304	19250907	21183348	23716662	9988689
27048832	21986495	9590177	23444367	19723632
23431171	8319905	19109890	22293177	10753910
16839880	12654245	17036197	9792683	18429822
22682249	7878469	27314390	16202622	12221077
20228809	17121812	32354068	16431929	17546647
11099047	15103385	29768694	14595335	10940294
20660729	8463288	31871319	8543060	8662891
17189187	1629222	19490898	15507212	22228765
22864287	9108399	22539766	9497258	15480428
18719709	15121853	27875099	21868368	12473120
20691894	20925119	27676292	16472745	10873388
25356872	11266540	11905808	17198702	15504364
17145718	8955148	29385717	15085137	8702756
18278067	17395641	24622787	12906795	8662509
20673990	11440997	12636908	21576392	8816443
15186775	7608159	12577064	24914801	17151359
17719541	19329425	28098758	10678165	8617235
11684014	11601988	32377503	16943183	11018042
22362889	15871698	9614181	20101212	20803696
15525938	18957201	18075254	21397845	26363959
11511361	16648486	27740624	15944396	28664531
21625555	19305001	28196644	11779461	27548350
16415881	16855289	11809807	16292343	22103495
20870725	20417600	18311041	10958683	31208035
22611192	22593209	19846717	17244649	16190977
17591690	21987589	19269969	11948177	16254241
17349958	15339662	10967094	8798539	22931484
15053879	9649503	27556037	9199170	15546864
15866171	15737063	11306516	21730051	29596304
10710310	14730319	24613967	8756646	31514269
10673501	17112726	25482645	23875749	22260703
10570149	21878504	12456725	19628035	23648569
12064478	2862839	31303019	16762923	27314616
20959462	29101239	22619228	1660188	31344837
11447225	11560503	24256248	16636067	17222083
10673500	18450746	31454517	16507994	12778124
10884347	23354059	30513825	11896053	10072071
12393879	28814160	26555387	11145705	24128008
11239457	11106749	17504809	17237347	12732141
11546806	27629041	29189096	7559638	24969695
12851404	15774759	15556869	10975528	18237546

12110184	25742728	11276226	30181355	21711248
16979770	11526392	11604131	11509732	21330321
11162552	19800266	18374575	12070128	20094046
8107774	12220513	16939971	12194817	21252999
12635176	10837065	18269210	10411507	27221039
19737400	11132103	20351173	15226403	15142631
8769096	12588352	8617720	33411331	36191627
11689693	20339092	21686281	11121404	10404636
19150423	20876458	23770676	29054983	10459005
9308965	18855616	11114305	23889475	12297041
3972795	21129147	18267069	29374774	30314968
1868835	18316480	20818427	17274988	29967253
1535687	21725049	19218920	12122009	21638049
9705341	12576332	18411265	11607814	31292270
9776743	22777353	8234276	30061385	18978815
20659008	2196092	21979375	23777859	26375550
18482001	16628190	8688489	32442405	22908275
3837185	7787779	16141325	24125182	30449325
18442490	25394790	12427531	16317043	19047365
18378697	25749719	12034743	33641239	12807903
9649439	22012064	12556884	34646012	25501036
16481321	18787208	20033988	9053841	17545517
18690213	11230154	32344597	23583981	9275162
10101234	19585539	25559847	22745804	24374327
9531558	9182763	32006019	25204415	24050403
15207230	15107406	32229307	24135138	12114214
15207236	22164214	14752036	18974108	26318331
17993265	12814943	28655883	20526349	18721752
20643813	18641354	32936320	24345920	10508857
22642575	21880786	20107438	17127394	19193720
12114023	27317874	16944206	7512720	1706616
24369424	23389849	27410728	20237318	22308027
25176256	26823759	24239741	18400509	24218551
3418702	26091038	22045002	26956865	7929212
26410597	7891679	10821263	26617336	22186019
8674114	14765127	27916698	16019425	8617750
23391857	19389811	28484373	16188692	16978839
24121435	11038178	26052920	8197119	27164096
22122054	25865393	30082454	23656643	28629526
24490730	12637528	26188105	12060844	24918289
19917616	16738314	30638948	7542747	29393890
22065625	20599712	30181353	17127320	26549969
17894445	16135801	30082452	23994464	25426117
15193314	26414766	30291150	22053285	27490140
25775268	10228168	26229154	9065403	22431075
11862453	18579750	29917041	11375957	21307776
20174677	8429029	27565685	9065402	19247306
21956940	11865982	30747242	11689702	15863038
25590339	12042069	22890707	10935490	15567848
10533284	2485589	16873410	21367659	7917292
16873067	19659435	25096182	20226962	16140923

24588013	20439427	15574878	19927149	23139211
18056464	17937601	12530964	12787574	8553070
26134678	22289780	15472075	12671646	2418960
23298836	21677426	803282	25619719	22710176
23175443	22249808	9207787	24034695	7524079
23558953	20198122	21378989	16062169	17052215
25535896	23579596	21378985	23254191	1375264
22837387	20553370	16773578	15207811	2011584
20566684	21527729	11187898	22089421	19249086
25294908	23565489	21681853	29408302	4589993
26425723	21331644	11466430	25880691	20192804
9207791	20029269	16508304	15207812	8580068
8845844	22211890	10880446	29964125	15585754
15448004	21713385	25164209	8940083	18710302
11830541	23562747	25080992	23352614	22952392
17525745	20600241	11891328	21205088	10858654
16012724	21622208	11182080	18719096	20576942
19809159	22448029	22030550	22547065	19530173
8640234	21147620	21890822	21847121	10700176
9539778	22991412	21349854	25019382	22438566
21160078	19158955	11925448	26247733	7749328
10471491	27170215	16778208	24788754	6318122
8798788	22578774	18339869	18172307	17143297
11429702	18483217	11438922	25033798	17510365
9865713	17555450	29146722	27301673	18311776
19066716	8168137	11344305	22895187	16339511
28751768	20889717	11119607	22575959	10818153
16761019	20164854	14701863	20495530	10608864
29025582	19996456	18836481	27578174	16597699
31066629	1846706	23237809	18363568	12883891
27795560	16461907	25511451	19741192	23506886
17268528	18614017	25041739	26586761	12568659
30559310	15180923	26801977	22399681	25819438
24854988	19881549	26387946	16475976	16981043
19808698	25992381	9856458	16483568	14624363
24608088	10734310	8752100	7688988	18523892
10698507	10648629	29162704	17350321	14579113
28470536	15070743	27654855	19648010	10334869
21359601	15218027	25385546	19762341	15715662
15178581	7854453	11520916	21550381	16211368
14984498	12948444	8621434	16636301	12719981
11290575	15035987	19573017	30184463	9096318
14759363	23352452	17686986	12150994	18568018
15549094	22510884	9078261	17936557	14657019
20868367	31059601	1653651	9809982	11274402
20452772	24211266	17967490	19834456	10022869
21224387	27264673	7961713	7777546	12592392
20412773	25137548	25196463	21358634	10934479
21233212	11044625	11253356	7761852	17716241
11278283	9244301	10833508	8752208	14770312
20951342	14980219	11114882	9214508	12750889

12759754	15774861	14744778	18391940	14729977
11279194	6305978	26832794	20378837	14623878
15522866	17597759	7891714	15059920	15126337
23453951	27002219	9687504	23236473	11463392
18725941	11969205	15870067	20531387	10733530
22438257	8078928	11100470	12897130	11511362
15498859	721806	1333888	9712883	11919562
23103841	25352115	7693660	18337750	19040420
15003515	23971743	11779505	19276069	11054413
26716518	164179	15564041	17146433	15377670
20807551	18718904	12482908	22252318	23059823
16219661	12359733	7737999	16140933	11382926
21932071	17170135	15761148	9671765	19615968
12907679	21173190	22074927	11896574	15781865
18391176	12216837	21350678	14968113	9858527
15728426	12221122	9821950	11814693	22012619
16460286	2332429	10653472	11559530	26773055
19783045	10805725	22074928	14688482	11350971
19278523	18524850	15384919	11278253	11988847
22277752	17556371	20577264	15580302	25429106
22934072	11733541	23236459	10973264	12771027
22394562	11279162	9724637	17284521	10984493
20890285	10559940	17332413	24739573	20096807
19926846	14527956	11191051	22802528	22988298
20871604	16399794	11566266	12692263	18187580
20107183	10224048	16923393	18431400	12170774
23604073	22647598	18365233	11574543	23139867
13175187	12646134	12612641	11812999	9063748
15173218	19896968	22421964	16772293	11971958
15173217	12536145	15814722	7784094	7834749
19923268	9113989	20019748	10891498	10807576
22732495	22984005	21737329	22802529	15607964
22918591	17928588	14739303	23485469	22172947
22812528	19074620	12181749	20460379	24096481
17031663	17263764	22017430	9780002	10524633
18031228	11564718	21173796	20817677	16249378
16095902	1499644	12893815	26122615	14614825
19616654	9989491	15950447	7566179	11070172
21892772	10375551	15976490	23703390	16341217
15209380	14685173	9926927	12606722	16857668
20164921	4754905	16728594	10777207	19521997
16608850	4507727	18198340	16135520	9799232
23441475	21550412	24366874	15577913	16144834
23026158	18321209	19822145	15273740	15226270
20551227	10727395	13298683	22766503	18420935
8162991	8988173	12203114	23365256	22085962
24868212	22371483	18344994	15289308	22884633
23736009	9162057	10716435	15735003	9501179
19037882	10648798	15665293	17442733	9704927
21736948	9850080	19439913	20353938	12004135
20565253	22735262	17540029	18676979	23954429

21596310	15824739	11672531	22721435	19531029
24916645	21637926	23166394	19170759	2295606
19843474	24384674	19480567	22772988	27155576
21829232	16936726	19128516	20417083	18436705
26344767	18852463	21044011	28602209	16365312
24636259	22961379	22909387	21405107	27067637
21716289	22722852	15493035	19389739	17335404
16080119	23150874	22463982	21216617	11104681
12473678	15643423	22443931	17289573	15353562
24555100	22445173	16978057	8404537	17313374
20392956	25131816	23306458	12734757	16336962
22679399	24468964	22083510	18690016	15298169
21425435	24680896	17068183	17581921	16806605
15696166	23882114	20207225	12845533	16011460
9620804	19771207	19755485	17693766	20430952
15156186	16420676	18029452	18195086	21111842
23045283	24469049	23248145	16461757	22658984
23442798	15475956	18035408	18400693	8382952
12732176	20028861	20027182	12110519	19153612
23613270	22606298	20300647	12838422	16537653
21906677	25633035	12923055	18767960	16782012
9566865	26208636	22394165	18682559	15220349
25123279	21679462	22122341	764868	10553003
12217689	20833819	28472341	5324173	17199046
19350569	10948449	19647095	24315934	12958364
23148227	11493559	9774680	25692999	16678095
11257229	8898218	11278460	24581449	21768372
22189971	18031719	24240231	24898039	16678096
11545733	30590909	22964823	25780173	19837038
15030764	10913181	30590466	25304425	21725318
26404249	9743535	16046443	25806043	17108000
26060017	24751955	18445271	25734984	23260141
18438928	16531405	20054179	22137969	22653731
9794229	29185068	8970730	21854988	17141155
19020999	27558325	17956727	21910628	21554866
24924170	22432088	17475546	21417719	22017973
17142326	19699815	16054042	20716516	10990458
27272193	20924107	7531665	25755220	19561074
10816326	20203268	17011499	16040803	18093802
22189423	15701714	17060461	22426497	9214626
15574200	24584857	18518822	21572561	15935773
16164020	25940801	16380219	16364630	11842111
24880459	21725048	10603305	15879697	18667602
18676844	22307729	15860371	20101632	26316498
16804544	29593731	21082419	12045094	11438699
23334789	17934488	3466163	17482885	17166836
21734270	16732331	10369688	37508007	12885641
19800882	19796237	24648516	32707086	31883789
21203558	20887958	11058110	38425362	26111660
18184866	29431622	11006276	11051212	24344280
21706051	20581311	12145297	34471290	11278378

8238642	10477748	15146457	25084529	20074548
28715957	9473669	30901624	32893956	32296183
10023774	17868694	11201742	10473610	9692923
26797144	9407135	11705709	10321247	18235502
29395074	9837938	20036641	10601307	17494760
17001303	17307141	32910366	19745164	20538072
21109473	12808044	12909588	16767699	18235501
11748222	12169098	16799563	30368668	14749374
23210908	10856295	12023038	27813118	11087735
11080163	10026224	19247433	12909369	12102554
12692302	8557660	12533514	23455478	15563450
9545647	8128486	7961848	9700206	26044572
9742218	28279197	10024240	24748541	23317503
27224062	10506173	16483939	29393141	16848710
11784712	10575001	9790763	9242461	31285543
9343427	16079250	18003636	11891283	20170512
29425100	12953056	11543634	31127867	24309898
11606575	9792688	23231787	9346935	16940157
12032137	26066539	19478182	19778628	23415904
9804823	28174279	19011233	28625565	30340023
30590535	9707407	9731536	21555521	10944470
9710638	29856954	12422217	24263804	15095019
28847827	23807634	29769719	12718547	25819840
31916624	16738128	12223416	29909984	31698146
25134449	12865409	15044604	11394904	20615952
10727212	10334923	29454968	12719432	24664998
9616160	26844272	15175244	1718748	21229319
9628887	21383261	29305086	15208306	23673625
19124460	30366904	7882974	25406032	21048217
18715871	12578837	19995937	9677374	21874273
24038671	17412710	16556915	20333297	21813643
31784983	23454892	22140376	30845223	14973125
23213474	16399079	31276219	18775313	12082086
23161582	30502085	12049641	22566699	26635000
12912983	17046230	17000704	16288044	25851604
11279118	23682772	16428860	23532176	8377829
26598493	20122914	9063746	11027676	9685409
11146551	24904170	24449907	25902869	18529014
18634786	15736167	16251970	28428259	9012831
27637550	15996652	24067371	7556058	11290329
27526206	8887666	23055042	11545740	23564352
15556292	24675081	11832950	8647288	26239904
16814779	16109395	9598310	28659385	10373551
15805470	14559152	9710624	21177766	18398435
11593007	10779340	15764604	1851997	31722427
25725067	15821743	18357469	22537386	24086678
23275342	29719408	26068709	9465039	16885160
7758584	11058098	23284264	19203578	10648599
32295885	15561719	18845538	15378014	19092802
12177006	32454406	16267379	25586960	31428936
19946469	23332760	7616957	23960073	9516461

17900573	29989768	11409944	164477	7681362
12963706	21865368	12067245	21802064	15544030
25393282	16826530	11440964	17386955	21908933
30318146	21639948	10506113	23423488	26235620
23091057	18419792	16815959	21131358	15610735
24308962	30742913	16385575	15295589	16244323
12493763	19956441	16970545	20486779	21310851
12930902	17660942	16584116	22285895	24852694
9751713	23475188	20681653	17612493	22569073
11698644	14747765	15590648	16529745	19158095
17432114	29279276	15039226	24797360	20412774
12514734	31034780	15020646	20417621	9228007
20375010	12379743	21829704	18829576	23802099
15543136	8985388	18579364	15864339	21453770
14530263	12089062	19455135	10830966	22927400
9384587	8591043	22678861	22921398	27410263
9334247	9108473	12581329	15363492	16493415
27509850	15371627	22790079	27313835	11875109
12393887	10567268	22302197	18782350	23261442
24222088	15818401	18547992	18434550	23516131
25569233	17116881	23437201	20048743	20117946
19001379	17195095	19857756	17055782	10692429
29604308	9616213	20067622	29343764	26569053
10546895	3027969	27754373	18328803	17234883
12239342	10196249	11329262	9472019	8702689
24488492	11779719	26668369	19245654	20698033
9228079	10867028	9452426	2567185	9094314
12766176	11679155	26855069	16364253	8826975
12192049	11762996	12917011	12740371	11803135
12171929	15803138	27339894	16105857	26490400
27789755	9727492	21159775	19596898	19683471
14623887	9036860	1238395	16479585	26387753
8093561	9971843	7217084	14651849	16916938
22792322	20447565	10898110	19098422	15581595
11438732	9321539	11294878	11069105	25720603
12764197	7511174	26545797	12392881	23150577
20588296	8181167	16216911	12502487	21440577
10642537	2871380	26587646	15661536	24379912
17255108	15546794	25577493	16027121	26499800
6867732	9770555	21636783	24135279	19324998
17376010	16170679	11360992	21569822	24262987
17464936	10756043	2339109	20033380	26254015
15134803	19762681	240819	11025665	16061178
18729003	11573093	19661994	18791498	10446062
16679330	15177891	26976652	12831062	19372258
15174896	22160858	1123350	15778410	8891199
16374430	28912259	21586563	12539168	17785634
18417113	19701189	8075637	9560344	22155301
18035185	11031233	18707589	17395877	33262481
18449520	18949601	14640697	20045007	32529116
12921235	12724730	23800242	11397779	32277040

32321524	7834621	3266253	18760377	9735376
31967327	10777214	16940415	16682204	19075563
28510041	9380030	10075741	10340377	16183742
20716671	16679317	15322122	7554031	11665719
33091573	21846477	15790560	22435563	20493860
16382109	956169	12727795	16360037	18075314
32142651	10074425	19617630	7948950	16582605
32283108	10224120	11991950	22435546	22854598
20600852	19474220	12573484	16101442	18804552
19617398	23512538	20399894	2147521	21737170
29737559	19203995	11120824	23425920	23416979
32438371	18607004	15541765	23150759	12726855
20457564	9704006	17901129	22435545	16360026
32680882	19366855	21124311	21541195	23296650
27825853	18089838	16545780	20004154	19181515
26551702	18336843	19106115	23263379	8617251
32711925	19255421	17952121	22083606	23239948
25904598	19158084	14978251	19230643	9108147
30101215	17934341	17595319	22260696	17301840
17404574	2843537	24261707	11859406	10887490
19079265	17709388	17266942	12214244	16314342
17914240	12223212	15340055	1653904	16258277
10618704	11114727	18288955	23238565	15603758
24743243	22167199	15501915	14599770	22263797
20808857	11502724	17284330	18424430	20679392
9821948	17223712	11903058	12214253	23002429
15078873	11259609	16197558	22588366	20354226
10224227	10395741	16289099	23721719	15254416
9799222	9504046	12573486	1535095	22980457
14512773	8910454	12611900	8985253	22410433
10934467	22903824	20619336	23747889	20061149
10982831	20395535	15598615	21807066	17581279
14555980	14597617	19471584	21187855	9363685
17164131	9712901	17944540	23638217	11595834
17494091	9488039	9099515	15947787	23802008
17975109	8937476	11343253	19125251	19075564
15180494	22659247	15004031	11089869	7488937
3619897	19574409	19129222	2142119	23303139
18216721	15837795	15897893	17935492	10037143
16258029	10398686	18848820	15865930	19586837
4051502	1844873	15489539	16115458	22160080
15748653	11470802	15496506	16082221	20515729
16399376	7557864	12120277	15686624	21903324
3977905	10620335	21226706	17151078	18677098
6548162	11097088	21420387	19582216	12207176
2834948	8396713	28760335	9261102	8948422
16179585	21856752	22073591	16398215	22522501
18383502	18827440	23150753	15136767	15147722
10759591	10961990	18047734	18508566	15082921
24467436	8120057	20813042	21722344	9681884
7889851	6095293	10362249	11832478	23519070

9466696	20305300	29653253	31540582	9822657
22038529	12963843	10893433	32850791	18184567
8301538	19890334	16376858	31843468	28473751
18796370	10526213	9218780	20945369	29249655
17622968	19683496	12481981	16984440	24741076
16998506	23788429	20301396	7485389	26103054
22911014	23308042	8930409	17644256	29126901
20023404	8782982	22116691	34017408	21193401
16024119	15254415	25514926	15296246	29038536
22743550	8666399	24122582	16621453	27499160
12655298	16397206	22417847	34149889	19073886
22265392	21034966	22899908	9588880	19426232
23388117	22084390	7741998	31974341	22116087
20224865	19581929	23142810	32102389	29339377
18940270	20006736	19369394	7999272	21904389
12045216	20445224	15621726	28771809	8596936
19400965	21535261	3798106	27648692	1191089
18497029	10092119	23220880	12618512	16551244
8533012	10582242	26028978	21538580	30511409
21089513	8533157	15805248	17869227	29347993
19707781	15670890	27251275	23792675	28990585
8939883	9418909	20453058	25087956	22437870
21963855	23200932	18676830	24972246	28643244
19686080	21593588	28348404	21602788	30345906
12660173	19023332	17397528	18184568	23108542
23717325	15723711	20570901	24225153	27106177
15483403	18243065	16385346	24859235	26187180
15865942	16903208	15086769	27449752	16672367
17626635	22844074	12373339	28634229	26218440
23554604	12963833	26794609	19812359	21704641
10713716	17611403	16217483	23027865	20392245
22704343	22515271	33073427	23103546	23748100
21326949	23808152	22092795	18555543	29587428
18368919	15254433	10469310	26071486	16936699
20465793	22761618	27477081	25530759	22226352
12171872	23208375	11573241	15689566	15680226
10673629	21391908	23226515	28608766	15922018
15496581	21615334	10550323	26585419	2203193
23070009	30081750	2656805	28624624	7720105
22991823	28933638	27256813	27922662	18423897
17251377	30501132	17576240	29133590	16112078
22150313	30140075	15953044	28642704	16402899
20158568	23877423	18597619	12386268	23951310
7606818	20186705	25837671	29431615	10640766
7672812	19590688	11685189	26824050	26244871
21329706	11952781	28182006	26750873	24161035
19364923	7889863	25876136	25616441	25212687
7787878	17702526	15625461	23313043	15212335
14593728	12510015	25998853	20357764	21795548
7587079	15660110	32104287	28624623	21173082
21196225	9118888	22848228	19073885	29127110

29427249	24979721	22403545	19834513	19898483
9733515	27694978	7753047	11875025	20463033
20621048	28329682	22715882	25475423	25080474
16005139	21613227	6321612	25176654	30587505
7564239	22306293	30886620	22652185	22083728
20847235	23792563	32116493	8123671	17380161
19524112	24058770	31439935	9694901	14730303
14239091	8841154	16525119	9582303	19111245
16602100	16778732	24077738	8672428	15545627
3769199	10516633	31175934	16854371	15668327
6774165	9010622	29606485	12011101	16921403
12235098	12811366	27408775	19014978	16805667
25646736	12492606	9741627	22724020	15343279
20923771	1867957	27852311	27547445	14993903
23558541	18187562	31126986	27047494	15688066
9291139	12950465	32094408	18787044	17943134
22156476	11186130	31351098	29100090	17128209
12821112	18781855	23443243	17522159	17060944
20420946	15886284	28476236	10340754	15339658
25736321	15051713	28588114	9719154	28339062
8898652	8875123	17126425	16396903	18004398
14715079	2049245	33203880	12766769	20610534
18316791	18622261	31708432	23064016	22941656
10479724	8818573	27009876	28566479	8513149
19679400	12895198	29908837	29194579	8621488
25236910	1782973	27879284	22510445	17715138
27173435	16920476	30328953	16880404	17765923
28698599	18719619	26239609	12414726	16436514
7697895	12920168	17186031	29100091	9199932
27380651	15625333	17382285	9675033	19525936
26161337	17724700	18483225	15838507	11809771
25322271	12185559	18277979	28739660	7915517
29553041	15530129	20117961	9361030	15377654
9405715	32760717	21305127	15327782	15225546
24790347	19793304	22387373	11203700	12919958
9046958	26923589	10969042	27597235	19168442
27068427	30479058	26979667	26604140	19202061
13211659	23901111	29149350	16971658	19586908
28423456	32451529	2143188	14656760	18509536
8001864	22885700	32344011	17537792	12086603
27047663	18179280	27363989	12060755	9765279
26956190	23661693	2856554	17785448	11877377
12788846	21505029	21742792	17462874	12364621
29224098	20720586	11460506	15141091	8175912
27163392	10904115	3021194	10021334	19793862
1594605	28345259	19008457	29945868	11027291
8839934	33670154	32339221	19809516	18619531
16024935	28258193	24611772	11138002	19854139
19129847	17051160	11076529	9425907	10608837
12050213	18560619	11731805	24357607	19652551
21325058	33599083	15122900	12783789	16617241

17460694	24104880	1840259	20360045	16633336
12447691	12215251	8524414	15886194	19996105
10556074	12011431	27067600	15190204	11865064
20871615	11369231	17525341	16810316	16009723
10499802	12814551	15640246	11239454	19111657
11916980	21283629	15870257	9733514	19151707
10884395	12676925	21044075	20413593	19995904
17525342	9705271	10364196	2882507	2564316
7961795	15317757	18006705	23007646	24293646
10078208	17049555	11537053	12354784	21978893
12717439	2050703	7606819	26675481	9590181
10373536	21659603	9716408	7526206	7700386
16862143	17478428	20079829	17621610	16530042
8146338	9564049	18319725	20023648	17939684
18001825	15205463	23166356	1578192	19328070
11157767	19193796	14976165	12199140	15279788
9278511	22154951	15680327	8665503	12147700
11051553	26512707	19567472	16899510	18212045
19966300	3025664	7958836	16628214	15064416
17828269	17194776	27723717	15574326	8632903
7939630	12766152	12402037	8991084	16712457
15897895	16547522	15221963	8769649	18596042
10078207	20171170	21857671	8640237	20364141
20061803	2946935	15064730	19652550	18172165
21419344	19338310	11202906	21475307	10999600
10713044	20064462	17558410	15671039	19473992
16980960	10373512	21639834	20348101	9828139
16707425	10205172	9435225	10744741	7585968
20020535	18172500	18206974	2406247	12939256
3142690	16189514	2195549	11573085	20445207
21120944	11432836	15527801	27342858	18172690
12724401	16360315	10981963	16199878	17704056
18082599	7969176	17412408	22508508	17312392
9660782	14734534	16236519	12228710	16260474
24235147	18443037	14695167	15199141	12890688
15806145	19344625	20826806	18974355	10679321
21872579	19203579	8170954	9518481	20173098
22867704	2142452	10910365	22442688	16787914
15899892	18001824	1332913	24627472	16153896
9822679	15356634	9836640	20643585	10660545
18519686	22237204	20634189	15485915	14636568
19661379	20231364	12192000	1811480	12781359
15940266	26196677	21172801	15272308	15201865
14695475	16943440	16531125	14636569	9637771
11986308	11971963	12384589	20102227	17227144
15122316	18298799	19261749	15299030	20639885
8521392	7838523	12419324	20046100	15454491
16116421	15195100	9030781	16205630	17599047
15115758	18644834	12470949	17643122	15661742
9685493	8843195	7961977	15314187	16698308
2573657	17396150	8964493	17608804	9388480

11032027	16814252	23680151	14566050	21042587
11157805	19907496	9636169	12209014	21161613
17616665	17611284	24811749	24100029	12526805
17959650	16087684	10608806	8247533	15117943
12019152	20729858	10448035	17118716	9590180
19109555	9052673	24220101	7905912	14729973
16908529	17088560	12191481	16595695	9207062
12414623	2243768	15377652	15451423	21319273
15096610	12824158	16142238	18660752	8640235
11163187	12857880	9889122	15096578	12205100
22357538	8622991	19033441	18156970	21504906
14500819	9766667	17954613	12419808	10839544
831811	12080054	18066086	9572863	15138768
21285353	10329681	14988723	6273595	11784855
16247472	23345434	19283071	8119945	19097996
12578958	12883740	15315825	12750383	20656690
11018012	21242293	12794064	15616588	20147522
21149266	17643121	9933573	10684855	10777662
9452416	12649176	23030715	19446481	7545954
16713580	26778126	14517836	11090622	23849169
21789020	18442975	18356527	18832153	11025664
14871897	17563354	11454867	15199523	27428775
17314514	23467123	22908299	16141202	17767920
8702565	10468606	7792600	23361318	20671765
19535328	11331310	10477523	15707391	18775730
11057907	18510930	9311737	4006916	20639400
25813721	23525106	12447382	17634560	7697716
9699634	10480872	18417535	17452773	19440044
16365875	16546998	19589784	11418864	17588522
7115720	14681192	24332808	17611581	11551919
10551855	8990123	17923702	12167711	16731526
11350926	10673031	15279789	11301010	17115032
17081985	18285803	21362556	11239453	2565339
12810625	15456891	17273969	15811850	17030982
22013166	8969240	24526736	11395493	21309033
12446782	12852856	8943031	9430682	17495531
21798247	19024604	16581787	6704953	18550849
21558276	25609649	7923193	26582912	21501958
10391891	10888888	16371510	10498869	12239151
8837778	23540691	16377563	9651580	19197159
15743907	12612651	19579266	22886304	14742437
25465621	11863428	15247280	22589541	14559997
23083810	26253028	19270065	22002537	22517901
9461304	15220350	16285702	16049003	15592449
10552928	20829486	10359610	9038370	18048416
11459832	23364835	20936109	8589730	21198351
16479174	10364235	19464297	12414651	8804307
12734188	18372919	14734805	11842105	15111055
9878247	9925639	22139841	11537315	9679063
12228248	11371615	11821419	15694335	16027118
12140561	20304803	26133775	24485656	16467875

12504096	15782130	12704184	11106734	17942393
11278446	18309293	7774019	19444312	10973490
7957065	18480403	8621570	12556559	15196461
8589678	20932174	15775963	15361825	20424263
6602790	12537559	1531147	8566796	12654198
25933514	14983014	10208430	19788416	14743218
9450543	12370410	16537486	17157251	7715730
18931676	16818604	15659650	19483192	11331603
12145306	18283122	19160488	11447121	15342490
18519640	15650047	2139805	11114745	11550094
15280377	20009512	18644861	9295282	11454856
11836499	14605214	19759395	11314038	10464290
16431910	16794254	17178852	16122425	14636574
9427750	15279791	10563794	15650050	9121459
20195506	10859164	16223874	15989956	18449195
11063725	17553757	12773400	19465921	12782307
15226314	16601680	19556969	14583606	11604499
12402044	17638878	11726552	18025084	9351817
10526407	15364927	15538388	7715731	10950869
16912045	18077418	20061386	7596430	21199877
12709442	15235112	24079363	12814430	17102637
27611684	18317453	20705237	20212043	11583998
20360007	11077446	17101782	9045680	19629043
12607004	19185524	21056556	11333291	16288057
20858735	20729856	16793542	23521171	8946918
9050866	19793861	16311512	11053413	11931755
20413589	16622405	11741547	7594449	16438930
19423707	11325820	16478997	10206961	26802432
14724280	10320477	12046007	15936993	9751706
18594563	11687627	20724660	9267021	15618521
18848520	18418389	15314022	15310756	9168116
17124492	23715498	1393148	12576443	12861053
18158334	18239466	15960976	16510573	11298456
12607005	17804464	9822680	18285460	8358790
9649500	19261748	20383123	16930133	11252893
16086026	12379650	21145460	10608812	19898529
18948756	22973052	11721054	18662573	19605351
19202191	18343821	8610130	18566590	18948948
23230272	15542852	11166174	8521816	11336668
20937773	21699228	8769132	11864614	18158288
12697768	17636252	8056767	15220930	17899380
9756909	19908865	20154705	15583028	11877376
8896563	9806842	9812896	11278964	17459151
18769153	11390642	17889669	9037071	11709054
15149598	17097061	14966270	8918887	10724175
23748380	17173041	18280240	9109492	17974916
3257477	16912307	17189255	14561771	19494828
17525340	12607003	16278218	15003516	15574335
17887956	12676583	21364637	16689829	10713175
16873062	2308592	17963495	19525978	19683501
10764811	18077395	15811628	18305112	15567177

17110379	32486270	16973150	15542856	12015981
9802988	23838442	19666824	18403408	14519196
10611322	31612241	23643939	20805471	24929628
21104395	12045100	23022961	19544440	27740627
10681541	11257218	19825829	24823357	24384374
15949439	24409201	23123965	24576030	25307053
19962312	23352243	23677624	28041631	29158817
10364241	17065211	16258936	1722028	21798082
11389439	21855102	16738056	32353859	26247089
12149244	26379663	19439460	31226023	24426196
16464007	25396298	21284982	21543844	30249788
10487762	25741013	19915529	11163209	30420806
9488720	23870315	11562345	22028656	28882953
15688006	23637409	20833363	19304306	32198194
9476899	24473128	26847180	30425656	25470695
14676842	25136083	27098840	21285253	20301485
10228148	25642836	27374873	29258163	22461740
19468298	19694547	23496208	32746883	31676439
15916964	25493356	15184648	26269761	14592533
21245467	16698996	17463250	23856032	23055695
16904321	11461707	22773810	24556840	31462513
15071507	16140752	23533263	23723077	29100061
10362363	17718913	11397666	23045246	28766509
11314011	26516900	25337673	25656897	32120838
20655466	19153231	22426421	10334664	30333156
9922454	11017109	23404247	9371269	9537421
11988839	22031933	19419954	20573930	18768914
25778702	NA	11751408	22334892	25110901
15574327	26771495	25115383	15607759	29298899
10203277	26246577	24535670	9380738	26725424
10097108	23573288	25984556	12587805	27809445
27257257	14673115	26139350	12727798	24404629
20932475	19625394	18761324	10963623	14673705
17488475	24478428	11729303	16280036	12522687
12024041	16051304	1597188	15535854	30307446
1055055	15103332	10622721	12714703	12910492
15122335	23555248	6182444	17380162	27457486
20019063	26487564	23746447	28431792	29057873
20841568	23342373	22902835	23519123	22474067
7671312	26861015	30030361	11040209	28098593
11076961	17005688	18682835	9585500	25328986
7983002	21866103	15867910	25429310	32557261
17428792	17339424	11602624	23619365	21811631
10672017	22383882	18220859	22327366	20657596
18174154	10975523	18374667	23085511	22281838
9660939	26468524	17659995	23376832	26561703
16122426	18089854	23977373	19996457	12655414
15468306	16446357	23592794	17307989	28404813
28356513	16477006	23154981	20603081	25418138
23565119	18052967	22960178	19555663	27995415
20713514	22007134	21741376	11967526	33685683

1406652	12724731	11044454	16951403	2992548
19841733	15548681	8440384	9683613	18203897
24369348	9849491	19951991	9099683	10720158
31035700	18498133	16858407	15940673	15189125
30871156	2656050	228272	7577714	11307174
21633178	16709241	6371429	1848582	18420277
21636835	15638735	9767110	11519011	17283068
10914538	19219653	8652655	15851485	2167130
18803879	18820913	190267	4774123	10471130
2899130	18267032	19747065	15536089	10833329
3790257	11956089	18583509	19740703	16547353
12856180	17549067	18522490	10048303	17046972
20354580	19093176	7649494	2870496	17389926
12479567	19302291	7278682	1652755	10100195
21504868	16510598	18198219	1708392	11839807
27477280	8996164	15668660	14726604	10216279
9654093	17283124	11325678	19628033	17508906
16424607	18992248	19854035	8846925	14976195
29352142	18390668	7639730	14642406	17912575
30042655	14555507	3918580	11878747	10101267
31101865	18837291	12432931	18424738	15704532
30110365	18075467	17439363	19024248	9442035
30925886	18607850	6884990	8325534	8769129
33268902	19144510	11447214	19625220	17916561
33043019	19402749	19706381	10980461	15063746
32760721	15814641	9325339	18938145	2181276
32015325	15901346	932041	10802064	15317910
32256352	16785472	18428149	14749265	12775843
30921410	18035049	10544287	17463087	9112289
31740582	17716232	19375431	18680158	11964182
33495651	17695509	6814427	8986768	1542667
30808384	22100631	8567691	6300970	3013986
27987249	11972054	16887964	11121721	16047261
9831565	18987785	18656701	17583675	11761328
12899623	16413410	15925209	10653827	11956665
15173886	9075838	14677018	10914032	18479189
11891120	11939906	10569628	9873062	18409172
20644576	8534261	14704851	15585321	15026176
26353940	19403638	12895592	1618328	19013211
9851930	6418841	18598592	15680219	17016550
20639876	6122208	10842581	15780594	12569109
17339406	19660687	16698314	2448410	19014349
19252480	9584338	16430221	10666321	16962588
32182061	12432932	11264458	18562168	19822456
16267625	9085163	9737710	11215511	17526492
18357466	18032380	10191291	11460480	8877730
19339911	11090610	12627223	10705969	15817466
19077464	17081103	218223	16011463	374662
18172246	16574427	9627909	230492	8617728
19383847	16174820	15215856	16120612	18927507
1272473	15689384	2602371	3790723	9587024

10064852	7276750	9462665	27889578	17895972
17713401	12231557	11412116	14570712	23576886
11017945	12062442	19415921	18332424	18215622
8386626	12831960	9242711	14585353	28334053
19674315	17392543	19413181	12368261	28859574
18354163	15314690	2246616	22267161	19436069
4355366	19268692	11001804	11237210	26287747
18498226	9370338	18164739	26670047	26287746
19464347	11716958	17927969	30447757	12040175
1730245	16339544	14993240	21167873	12164919
18718914	17296604	11533328	17298186	34244591
10880336	16219795	12815058	22483044	16435184
19403631	3919061	17534535	18184796	3126356
15670717	4387676	874074	17644144	16483879
17511631	7288293	9368067	16055064	24168112
9570154	19769461	9852097	24727796	15471987
1848655	3099851	3046314	24346713	17263796
458381	2497518	16716149	10473536	27041232
11251339	17049925	9789062	10613508	25405608
698243	6311078	17477829	20188650	17384686
8386433	17198385	19521349	20457613	1782695
17612623	19803417	10716626	14676271	19272414
14580199	19167960	16835236	19968948	27127795
5637427	10448523	15680232	24966374	25690510
17311055	2174886	2175712	25113167	24726665
10484769	15104204	21996254	23202739	21226711
16251722	9133619	20534694	16908410	21345369
2610352	2876046	12440980	18474609	20183829
11929995	8301225	14500912	24533017	23897649
16202558	9730949	16459311	12563289	28203483
15837518	14999406	12522256	11837891	17076662
4086942	4690137	3091479	30402088	9928160
15016776	12679481	16648838	27257628	17637475
6295699	10444342	26215792	27766264	15885916
19825219	11861418	29456534	17496911	10980593
17365173	6113262	30076771	9839441	24568968
6758698	16319061	15467723	28473628	27410235
9278416	16990795	33178221	27573877	27592409
18524888	17276401	31986264	25781681	22076657
8656081	8968737	32513989	15673435	27359210
8413223	8806705	20410124	23641197	17143559
7429333	15976321	30705620	30686771	22308501
19557870	18757362	20505079	27847432	29183756
18541719	1377680	17084704	22017584	25152404
19619139	4386961	24044036	19345194	15934937
9501215	12209373	30364226	26468183	25017118
11413152	19450180	22510460	22405502	18272487
12432933	18617006	17088211	27239350	16819983
16911361	12730456	19602257	21162125	28196718
17960332	18394665	28183735	15219735	24602615
18209571	9535873	29410531	25729352	15885774

19276245	22761566	23775697	12919045	18854196
25449850	19606501	9131163	7743460	7951064
33136286	12106454	192727	9299423	10692424
11445798	21953180	3753461	22877991	15172163
4152527	21278789	9526094	2163656	22809994
8127060	21078976	9370318	2153142	23022039
10515893	21934092	8063717	7768880	6088233
29483667	19035562	9370322	6257301	6145585
8099811	19244115	9838049	7574485	18791037
4152248	14988435	1718687	8037660	17917066
18515354	25415055	19250975	9561269	2988417
2294991	18425414	1311951	21554199	9131118
238530	1323041	21979151	1323040	11286640
15717202	11740936	9654085	9370334	8777586
16618936	8382477	8405660	10882337	15979148
7937585	9370317	2364071	8869881	330281
5289242	17095752	6712967	1362247	1897971
8810901	11171073	993673	21282087	476064
6113726	19401146	8883840	20559679	23240538
21942574	19362164	1628249	20503434	22750097
1711780	16859663	10208837	8499439	9370331
23727593	5581578	5580660	11324699	1323036
7680650	18204095	7378426	17412762	9553082
7215948	8866672	8274015	1128165	1495422
11893509	9386267	1495442	20178759	10396601
6995243	1989575	10893425	10601694	16339116
22539939	7107629	6354980	23054682	1495441
24584707	9370329	1546171	20167241	9370315
19112489	22136116	2498871	1329260	9144082
29357412	17673461	8200054	2499328	23010477
32030122	8468530	23143232	18955040	17171187
20960469	2505652	16899548	22319379	17558022
32715478	5277094	15821158	9370335	7215553
8402000	12824553	17318530	2663077	17940275
12833157	17294083	8699417	9468529	22215515
18087673	677084	3071807	19413994	17079146
15208781	8440692	1819750	9370312	2832400
16175503	1333282	22981911	8993541	8391253
17661906	7961445	11330037	6263342	19318427
15485997	22285183	7537411	22892679	15481814
2446635	23775696	23611148	9370321	9458812
15821736	14759225	8391435	10729607	205412
15790351	23712958	18525025	6251897	12743757
25167330	2545264	2143679	17881348	6113006
15731287	18204094	9765874	9370313	17132865
15986374	12749687	1550861	20176101	19324408
17010589	22014644	21214572	8382961	16015482
20378641	17157506	22374091	2118266	18341203
29459677	236033	6388643	10761925	23394527
21210154	2083674	16495223	10358924	1932343
29030052	9370323	1717219	8765148	9547571

21077828	15361348	23945590	16524428	29577047
19539604	19422785	12943534	20201954	12435631
19540930	10905630	27217160	19717472	10949293
3440872	23295697	29666272	27428965	15145825
12038971	8709678	7635144	28632878	11805083
2096696	6297743	23871722	7757071	11839797
4092051	2833930	17986282	31949887	24991833
19666474	9370319	8016100	2573431	32205204
6288375	22468920	25270028	20381640	30406125
23350810	18621144	10581255	28911859	17667842
14626658	9370326	16100110	32276449	18852458
9590628	8786821	6366476	29761529	19893639
639820	15878874	21334936	18828673	21953136
10396999	21303393	11058895	15695812	23008150
18508126	16719778	10944123	24051395	26253919
19450542	4005284	19576565	23631851	23943603
4369816	1323034	11733556	3103658	24011563
21703569	22960354	11901181	27789274	31863285
22103853	3707974	12872255	24942883	24458711
15922587	9370330	15945070	23133399	17503467
10191259	5334818	11228641	23153495	25883951
16717392	3037250	18452889	22399345	21835778
19657568	10759022	17166182	18593557	16485879
8488570	22910056	24685145	17376426	15833859
1495418	16246718	18455129	23847349	15573099
2936199	10395968	10980531	31695792	21163869
23765576	19328771	8808595	19393343	22710719
3707712	21818839	14973782	19557177	23940611
880944	2543404	28816422	17350578	20725040
14604010	20651826	29459785	20691899	25703554
6268930	20153716	29297247	16990131	22872737
17182612	6705975	12480927	19620969	25241263
1323038	18295604	5288798	16914500	10901230
22609101	479108	10788335	19578358	9483513
7031820	10427554	14973778	27234298	9378992
10215861	8382767	20080937	22617791	19028820
15893598	2160964	12217961	24766805	11929849
23220394	7492326	9525984	20549139	8156598
6264966	12573444	29874875	21139080	19008118
8389973	19889969	10835346	1451437	10673356
9370314	17063928	22304930	28042609	19324969
3818635	22960381	20637498	27148389	10937998
17012796	17069352	15148656	22128289	26830228
2833508	5083874	12887896	23633457	16858403
9083101	22628558	17273964	25564569	12453919
16838328	27304503	10527672	25018647	15317753
15134747	11687968	15741281	21355094	9233789
17928411	21336310	11836223	19490906	15165456
17646670	27591812	9399852	22088887	19342379
3300655	29362483	30705290	17304241	10959075
15273989	31920721	27720922	29565815	8175923

23016862	1280824	2227617	28826372	23399566
12006103	9196040	15065877	8839927	9054771
19135507	24696235	19528880	29882869	3283935
25451943	7988557	18022819	19689262	27013343
28844715	7665574	8681959	32633718	28930145
24243012	9374471	12949720	29354093	29163511
1890850	10514501	19840950	25748677	22824096
23400783	17968323	3218790	11831846	26926090
21359530	8232552	16274220	8016083	25254104
20086245	8181059	23793029	24363178	25326323
16125000	11574262	30843452	1653609	18755000
19461653	11574261	18950740	31790802	8634065
30578699	17244752	22002721	31954874	12446022
25311867	11242034	26305592	31676443	16132226
23162553	19302040	18694559	31812582	16888915
27622013	17442919	20512146	28118532	9831245
28477742	24550720	18376416	31616242	9286353
30504141	22307082	21266464	30980044	15491976
29660367	19229310	11980706	31736978	16047947
21654544	22465036	23150559	20811799	12115225
12355441	30065109	28800946	26186194	23380452
33363734	12893990	23532844	24438557	28527011
18803764	14646693	22179047	32111819	16838012
31134055	12730278	22152675	31819986	15817569
31088566	15608127	15224133	28888937	30054974
11698225	11990381	22863007	26165754	17267393
24780758	11602529	18772192	24627487	26447148
20536554	12810652	26982032	29945215	27105113
18794151	10815927	18413257	31602316	19851446
20064376	12960109	16682973	19430479	29925282
9872990	10197614	22121117	10799542	21525168
1496401	12181437	21422230	16497175	16600869
8628273	9466980	12403812	24239284	25762440
9857039	10220571	20375344	29597279	22922464
7608146	9950149	10764818	32469225	10100484
1638633	21566147	16760425	33664446	16278232
9724754	9242408	21725307	32159237	14615802
9326223	21036394	15107855	33377319	29286006
9295335	18794901	18371931	30654597	21800163
8496154	20685892	10806194	33571544	21936831
8524272	21543532	8798640	32155444	19509293
9287210	16227996	19625297	32422320	28870987
8780698	21346250	14623329	26271607	29072575
1468582	21252237	29186351	1608291	31867277
9305869	19923220	26411921	33505321	23395095
8550573	15755449	32273485	32130973	28327630
17604604	21699959	21664428	33170317	29371938
10022928	19682329	17054399	34281182	30073421
2153461	12547239	25985275	29167338	32768523
10428508	5075227	23596439	29256392	33329574
10644731	19381358	33454021	27180971	33462414

24780002	17112607	21423409	4061122	20303741
24607545	15781663	17573714	190272	23898905
34073720	10646883	25458568	6961921	28910500
12612292	21924373	23960241	12016260	23583456
27300434	28323937	21070191	18499582	27562463
27507853	9727023	20368621	24808179	25277212
30101371	10951588	20697302	15728179	29251630
23550303	3283656	28522374	26965621	23622250
9378538	25323927	23611944	19139765	17468766
32317288	10468914	20400852	3630977	10086361
12589646	29433126	22334035	17215125	20407820
31285550	16785999	20010955	25108285	9253712
27282309	18668205	16251272	12730697	9488659
28614305	15982921	19554514	11717312	19018142
26078352	3283542	22817889	18039658	11055975
33800494	29610148	26091043	23475612	18342376
34226685	17540175	22453014	22236406	10189350
28740119	3491291	19718025	27068984	8621626
32882916	6308607	22842228	26224785	10864911
23975423	26878173	22622578	6805319	10615383
21484256	12670889	21946352	168823	9568714
31283845	12866375	30987166	3477815	10827130
30642555	12114746	25059483	24288038	11101507
26358421	3043188	24732412	23066022	25903473
15686623	11278720	25603176	7730305	21205967
19753302	11278702	25738837	10672230	27568792
12771921	17726008	12618595	8187868	17488487
33232793	22539006	16399907	13018271	22193159
16216411	10970876	15616152	3052428	20031384
27915330	30210299	24628039	949837	26655797
24758178	11050117	22225631	15234968	25863248
19424592	10756053	27441728	7961626	27036018
14668814	25011106	21331042	12234803	18695042
17880691	22721863	22545159	7730304	19826040
16231422	15308747	15986483	8506365	19141645
17377501	19157421	10617567	7060582	20029046
21991364	27352031	19561075	9765290	19202062
22917536	10490655	11260262	3724458	15944709
15689376	24069510	21266536	17558466	19833767
9513715	16613900	7426196	10781873	18066065
23840669	18820302	3004475	19696787	18521080
23154416	18203756	2511019	20371350	20351064
18562482	23431031	8939939	8393662	19258499
12907752	24662006	21480869	16435220	20406979
11146549	24615633	6403642	6822523	17135268
23404106	20716963	7327552	16807786	17656095
16678816	21212100	16452169	26536169	17823410
18413260	23351786	194920	19496715	19221490
18681747	20452318	3944267	24812413	18538733
17540168	22615490	15071125	11114738	20018759
14602780	22141737	28538136	22042966	19597470

20404092	32464637	23238060	28438858	28086984
17135249	32839770	24622826	30084000	19166931
19377482	19692591	17086380	26116534	18466115
19034270	33019591	23176034	18469807	26678875
20080624	23321557	21778025	21460185	20505359
19273599	32574107	28003096	18253061	29409688
18949056	28956771	12163693	27856613	22221393
18701644	33937724	20446114	24528886	27416781
19536137	21703540	24959120	26039999	30619462
21177881	33015593	15693941	18719102	20083114
16111679	33116300	11701706	24472220	20356743
18579786	31115493	11854458	12062040	29233870
20212154	32995797	26785480	19211685	27412492
15077171	17451827	18429699	27882935	25012593
17942906	32818486	22971926	12598900	29158945
17784791	21187859	26890602	20693423	23630460
17569667	32728199	21646373	21238923	23087426
19120703	32979938	26686024	25288737	21779718
27903835	32726803	30778219	15729359	17440456
25403569	32699849	26009982	27034160	29898976
27720676	21597473	19052657	10366627	33510058
27623250	30449619	20725615	11387206	23853735
17383918	15504727	16628003	18216145	33511116
20816094	16261263	19470756	32555321	7615345
17557076	15103690	23751779	33422265	27187935
27226634	14645660	25770769	32691695	15291819
24564666	16803896	22209643	32376634	25598354
24838397	15155839	19643170	32307550	30459650
18179882	12805287	19206551	32492406	22287959
24089531	16452680	22064246	32733001	32932623
9837812	18614672	22107733	32838362	24742457
27626371	18317590	20398356	32228226	33239064
25678554	17941718	22720979	33845483	19909229
17344420	17005849	23428231	32048163	20842175
22982022	12629174	19516051	32619549	33061891
22356826	15963952	21593791	32132184	25078115
24344204	15774771	22098780	32511476	27443914
21057504	19179283	21974862	30190671	11403878
27215383	12196911	21979174	21108791	29018191
19463981	15728187	28438689	19575671	33050345
16200211	14566944	29991871	16402914	28603493
18614015	17032905	18391788	20083100	19903816
20858599	14697674	17229764	21784065	11908751
20818383	16452991	23520208	15731351	12827358
19752196	12361670	25667580	16845469	15734728
23260140	17188889	19273616	12815623	19184645
16218961	16055563	22231519	14973296	21784059
19028688	17067279	21172653	15944192	28167679
20226757	18674600	29375318	28561066	8074175
18391175	18445122	25483983	21628530	32604946
33106987	19494120	19543238	20542007	17186029

18161746	31495888	24382962	30862117	30578919
17110941	10854423	22652455	25823658	25092323
16823444	12034848	30207631	24459210	12077124
25501551	26627236	11299310	18311132	11013305
22705006	20368362	26170736	16403804	9660774
30315846	16473935	26064108	29693488	32696532
28892558	18079701	11549701	21964294	24288332
19352614	32358495	24068427	23644458	22465940
12891546	22492724	18599270	15328537	22366074
16364488	15210332	31091547	19587107	2335522
28983452	33010169	27237973	18847334	10748047
22570745	23906714	10884055	19917303	23273843
18242193	16751180	33613258	30936491	19805370
20303879	22453236	14764632	15864272	17872378
32584474	15882621	29413903	20016026	21796212
28341729	26865925	18198212	23858833	8790411
32685191	17643379	26220343	17607736	10588945
27117316	29069470	24333629	23882024	2384150
26950144	21855803	22550093	16456071	25104388
17328863	19029798	31667577	24724793	17873880
28572459	29955842	28667055	23224777	30471425
20418096	32235701	23349191	21353301	24735479
23322901	23901102	12673048	20713077	25271621
25871831	20887785	11435939	20628458	24491228
9245493	17872503	28487995	21793770	9013544
26485378	15610069	23284041	23322373	31009661
22819548	15907797	27667570	16697548	12569201
23434765	11038252	12748652	25739888	27647924
14570043	16459330	16012053	23865044	33246156
15923610	18505913	27543160	32700336	19996111
21189866	16547273	30992313	7654171	28923174
19321346	9388184	34501340	3881803	7925343
20104017	18430728	34786213	10600673	18621681
28618167	15012602	32403258	388618	10602018
27542226	21548952	10675335	15869601	19502589
26795388	12911774	17446932	11771674	11673457
22948112	11207308	20716577	4381909	28322867
34039996	17471233	16115815	11340051	29960034
31572065	10356359	17227891	15490415	9717719
26209534	12947119	16478798	4291593	21796211
17631638	18927239	11285237	14641005	16685654
19909241	8019414	11792809	10477257	10097111
30216699	12466265	12370805	21576599	10067858
17974973	10762064	21828285	23759795	10487205
33188728	9242908	14755334	23415802	10207619
16729019	17389618	22103516	11089551	10557099
21452186	22634633	18371421	10655068	11163208
23045548	13271447	27865926	16385454	10498912
21676658	8752328	10984438	9614081	10527909
22118460	11158046	24123709	10377398	32404993
11972036	24515297	12960086	17428920	29099489

33932560	9367159	11553680	9971736	23817417
29436617	10716940	21161717	27941249	20400695
12759238	15902656	21283639	28074012	12010778
15952880	10802647	12666113	8019699	29247122
15503154	16554828	16295699	29425059	32398875
16022590	29056325	19061375	27288456	26505736
12716939	31035587	17567803	12919684	30381825
12783850	18215151	20067580	19043080	19273625
17911161	26516076	19342987	19536196	32192578
19956200	26705830	11585624	25090446	15978547
19524505	20811577	19770513	23934149	32407669
12628165	10952417	21168495	16484492	28583370
15229644	11714087	21092735	20870751	16785567
18426756	10528039	16498630	21839365	21595737
12728264	24759575	21657851	20130528	26982353
19088304	25660022	21868473	23502960	21715307
15896322	11357143	16623828	20537521	29935220
18680555	15784165	23152062	21125407	25229003
15651302	17360849	22399755	22958489	9792917
15054086	18842113	17629414	22334613	22014308
15505146	9512493	23035104	31796734	17277314
23383003	21620960	22536415	18796515	12835412
22432004	16847462	16504406	26993153	11087758
15040260	15209374	20547125	27437668	18508192
23685749	12531180	19350383	27871366	23568971
12618436	12660731	22884909	32153505	20943776
7565688	21779440	17917587	25620207	15375155
12368238	12048182	23045704	25727005	11672424
27372738	21440011	20202079	27320729	10785607
2841963	9838078	21771809	29381406	22307589
21769672	16187290	11059815	32439107	11017125
2557116	15505410	21640756	7796919	16492677
22512244	17496910	23027386	33324646	31286677
2545232	19363522	19932745	29587204	1978318
1661577	28554312	22564823	32549377	25105124
1755382	29348254	19770517	15616590	12446672
2546599	21673720	17482149	33811820	12716889
20657170	10094148	9108119	28803844	26308067
30124109	22930444	18930956	21191810	26327396
27660392	12861073	15649943	30930090	26582078
29793167	22771695	22694955	31687975	25126564
28467092	9872457	29499229	20388082	11514575
29778464	17698296	9153397	28298525	11788602
9714779	16602711	27931246	15367596	18695242
9416770	20304963	25002992	23575689	18316376
26590421	17706365	21914775	22590492	26586472
27037577	18267965	23341784	16446378	9843703
18474859	20484625	25957321	21681369	16883319
4331040	21373950	8863824	20018632	15322102
8640223	21957482	28009282	24285838	24311738
16365287	18637713	22065085	20970516	19184091

15383652	22442671	15153069	2433278	15879121
21170887	9489617	15699391	15609003	15572664
23908456	18086946	17242337	25406093	10625698
17164835	32526773	20206331	15536068	11698415
28634454	33065209	17916383	19005037	10861044
17938243	32562843	16400613	15546853	10629034
17803961	33375371	18632736	22260796	11297525
18834332	10320667	18319072	2459516	1447167
16122728	1336455	24264046	16325348	9639556
16386727	10712923	25498144	21959103	15604236
9603781	27477490	19270536	20383137	12746452
17222338	26398287	11729304	20826676	9159118
17849436	19536897	33082294	8190100	12600988
16201968	19584267	9529250	11257115	12239223
18519637	22155636	33395426	10196191	15492006
17403901	29543893	33458558	12488455	10711350
10938085	23729401	33442700	11500364	11108712
30927255	15947886	32869019	12048245	11304531
2615857	19740705	32125455	11782488	14965240
15078215	19850741	32227760	15814901	9272731
27078878	15469821	23943763	12730329	15795236
12270951	7565706	32662421	15572660	11496823
3479638	9528081	32938769	7622446	9759836
22886028	16464983	32489508	12960148	14570902
15261132	15640247	32917722	11588191	10825383
31726893	12437990	33083157	11120810	9873047
15279543	19854871	32592996	10891480	8621725
30902655	18333962	33244168	11408617	9927426
3703020	27288453	32511376	10835426	9679149
18443746	27507650	32169673	15677321	12600999
28229088	22939629	33072893	12628924	11856330
8632302	21931736	32653452	10744767	12393899
3561384	19623215	33082293	11959661	10444401
15549298	22905747	32706371	10593906	NA
16141383	17500595	32404436	12242299	11916537
12919933	26153216	32970989	12495932	12070163
3588607	24058414	32944968	3141589	10716930
17077318	24164323	33801464	15488193	11701463
23539623	19262167	34127972	9328344	11082445
28939686	15136563	33391280	11708838	15826941
5323048	11238932	33372174	11080204	11454875
10989432	15752761	26579391	11292831	11245462
30823446	10611234	33335518	12167697	12896977
15093568	24920159	32529952	15064721	12855697
7936305	23752268	17490702	12496361	12181435
25030255	28295012	33101306	12351703	12446733
16541364	9885572	19430490	10783132	11859076
31744820	15917271	15580300	11907026	8900202
17462537	20463145	34788596	17492052	7797459
21508345	23116402	15681410	7731720	12738761
25595279	21110228	26260141	8633244	12414794

10942386	8622647	11032810	10751227	11053438
10226075	10022874	12498787	11751459	10431683
11673483	11839761	9607940	12690113	10196167
10781029	15156153	12782630	9528766	10753939
9169451	11441089	12588875	10958792	12970364
12595573	11058585	8887643	12226077	10706690
9379049	9218480	9727040	11400324	10827000
11245588	11038347	406657	9065414	12218141
8808177	10854065	11846562	8627578	9755064
9439849	11376011	7592979	12649265	9831561
10488088	11956220	9430721	15153095	12917107
10330161	8756648	12839832	8969228	11827958
9725212	14654098	11490018	15151905	12810613
11306453	10788495	10325235	12529253	15805288
10644746	11971024	9688849	7923353	15657416
11732999	9733801	8940180	9422727	12738797
11278709	14499342	11395491	11560921	10693929
9808624	8700529	11157082	15640156	10026206
8626650	11571230	9139689	11315998	9569230
11283246	9852158	12208764	12842874	14681544
11520792	11950876	11463794	12670941	8626761
15262987	12810757	8765993	10096607	9891015
9016798	11278353	15688010	7499206	9885231
12589052	12145292	17190838	14676298	9973202
10807788	14984580	9687510	12032150	11487658
9361191	9675184	11500497	11303030	12621039
10931853	11574474	15213298	10477597	12446726
11696358	12491767	7836388	15491994	10199807
9006914	11416000	10918063	11238126	11124968
9094716	10704466	11574420	11500363	9669024
8717044	8910314	7559496	14963045	14729955
11812784	14557275	10666409	11274965	12808055
15322221	7744823	14532295	12796499	12524221
10951185	12960165	10993748	8571671	10706116
14668344	15650183	12225966	10747974	14988405
12649327	9130707	11006268	10066767	8995385
7761832	14749369	11880369	11406578	16760378
9207092	11352924	10642513	11503142	15967790
8668348	11017917	11463795	7533300	12239105
7744815	11123233	11035106	12181454	11296227
10891587	8621389	8687465	10438924	12181434
8939929	11983682	9391009	15078882	11171588
9346921	15201137	12721328	12169624	14688255
10713965	7896797	10463587	12746434	12724418
11124982	14970203	10487749	12832465	10318869
12496377	10777559	10969079	8887554	12193412
8798679	11879201	11328854	1313322	10024371
9642252	11454682	11350959	14702343	10788492
15155836	11591753	12623839	14575867	8710867
10636870	11090049	10976102	12801936	9724043
12584202	10428835	8429046	8617731	10650934

9039124	12110168	15085196	18234890	17374610
9651336	25483003	17043353	7592771	10094440
10098484	16144902	14765107	19289301	22418790
11895866	18579680	10224053	19880519	17360901
9316442	18485326	20145241	15918042	16764822
12562867	15823095	19706601	22000980	16006741
1908778	11756451	11591434	20965158	18928334
8713997	16100120	15452130	9933599	18633768
10391889	26853464	16399501	16916793	16938762
7608156	17977534	10889017	10421804	18032528
11487632	33414460	20817729	20124538	15758173
17464208	30108193	22031849	10978336	18639564
8393279	34064003	20541520	21836131	18384375
15220929	12011103	23300015	16916659	19729586
11752209	34102611	10318878	20880945	22226696
7713937	32839400	22154484	19120039	16275016
9057106	29090099	10397761	19958302	19278673
12060777	4064003	22684109	20847275	7908738
8382677	30885289	21240525	21555452	17142880
1683468	30060809	15266058	20209124	17553693
10542136	27812877	16831124	10197817	18792810
15546818	19416851	21696544	12065627	14608012
11259513	19029065	22984430	20198379	9038229
14724213	18216770	20670887	20351659	9676432
16903857	17928229	15899885	15584216	8957963
9395450	17621255	11358865	17077377	9259266
7985086	22865681	9488713	17973628	9602135
14715266	17065982	19126544	18778441	2058745
11606615	17646659	18835813	14756806	18599790
11818496	20855588	18286597	17556193	18192214
12554779	17347648	14693395	17584746	11335745
16303171	18046414	17568567	18085326	20226894
10781004	17581637	18084013	17354259	10575032
11181532	20595232	17597646	8841406	21204411
16189297	20732877	15056938	16830328	19247214
10217295	17290267	22351881	19559077	9645961
17452640	21473868	14741405	14575242	18180303
9620655	19015276	21865888	17613521	16728402
28924389	10770267	19772970	11080175	18045290
30487431	9106493	15240857	22371606	18675942
27378692	8402897	18041756	16144830	19396608
12110169	20103737	20080142	21600882	7745608
15902259	21459323	18094064	16467535	19476549
17360776	19759400	17869437	12393272	18771483
29761784	10500120	19369586	19841066	22179534
16505380	21816276	17101276	20009079	21976671
16728472	21664250	15273283	18810510	15199055
16342160	22436747	17311278	18571009	18534229
19608404	15680331	20937660	17709376	16322073
18849530	10805775	19801649	12599191	11259344
12529645	17296605	19666841	22334710	12439736

21383205	31611699	26482876	30065554	11171046
22449172	17719247	19196800	21076395	28007913
29934975	8146197	28235572	20951942	12427739
16480962	7543139	28723567	28109176	9182576
17765940	35727133	27449104	24122795	23076213
17186017	16208375	21683758	23435428	24689035
15135306	17182562	17574003	18433497	28289053
19250197	1423621	31076567	19940144	12154000
8242225	26473606	18259611	28820180	25910937
16516475	11520798	27681418	17055431	21636859
17680028	23530057	26207953	21670757	16006559
17041621	11704864	16870170	15044469	11682481
19143648	11402335	16428285	25848864	19050761
16469695	29237129	30886364	33506952	12716911
25310982	28712664	221813	32726355	9450544
6998725	24324645	21455117	32695122	25956888
29608137	11057902	17974478	33596266	22124154
23791173	24069422	22649723	33337934	16893970
23334421	22383895	14751757	24493213	19295129
24449267	10964259	23634849	23483280	23830865
23685455	17452290	21047732	29180617	28535874
24799285	17666482	11830511	29107536	21192934
9489702	24872083	9774969	28658158	11387210
25435019	15964803	11423969	20301631	20844008
22066971	20167242	10391251	20301338	20826718
19375514	23045339	17139329	27132995	16966330
11861617	27351946	29892481	30337552	11744618
9784967	21511232	7799943	28293384	12588761
15246961	22653837	17167474	20301360	11161716
32726801	28322461	21807935	30158522	10938010
29517999	11149600	22969750	16940241	21148427
23405894	18329679	20530740	30588060	22592917
31996678	24463099	14739072	2722838	7657594
29568499	27693079	21193034	11465067	18206965
12172553	28448947	22079269	11532180	10067868
18991293	20802499	30456354	1988037	15928081
19143635	8222576	38260464	14597963	14525795
1847722	25330987	27641100	2112956	26221892
9429890	18467435	19932708	17687269	15247219
11395409	15971998	21189261	16945988	15180964
15778376	29997323	17161604	9353388	9858476
19710469	21317437	4400078	18838507	9169492
8676075	20378719	19826765	8168173	17686471
15546391	25390014	25860609	1676665	16627366
9973453	21448409	29902437	15761113	30086460
29915297	24820868	31825083	21843514	16336951
23922331	17574010	31258331	26655500	18450586
18515054	11533250	23797870	15448146	11494124
11406366	20051527	29468141	26136364	23718729
17015696	22353746	30619249	23000962	10688886
28851713	17720805	22688187	19181962	20507983

20332118	16974068	20813203	17276402	23592840
25644401	24785348	10331420	9405464	18723443
21212275	17178724	16484616	15548136	22145046
24662486	15207703	15184502	25387128	17658244
11108718	19494114	16679383	19923922	15692085
23029280	9393975	24746698	17993608	19715393
25331892	29222111	19228841	12490545	2656356
19435802	14514674	15920022	28183800	9453379
11090059	28418925	19718476	10022890	8878137
11335727	9461619	11228166	11678628	8948389
11704645	16403913	21586748	9804796	1444060
24931163	26063728	22689825	12244099	12806616
18952368	21169383	21245381	22264731	1595584
9299537	15545625	20432469	15854902	1416048
7896817	22101521	23851566	21873429	18289917
11696015	11278553	28946938	20422004	12401878
20400538	12844492	17065532	24966171	12937841
16479592	15026417	29051140	16787925	10227052
19965691	21653826	9722576	20434959	11585356
16909199	22854047	12086892	19914243	8017322
18787075	27052191	26846344	11927607	3372162
10544009	12654612	16760434	17060906	3019190
15051508	10347193	12214271	14525763	9627692
15016650	11719508	10048588	29401587	15358783
25157100	11278468	11238453	27695625	15911617
17004325	10961983	15371454	23341459	26364851
20129920	19038867	21524749	15637071	19700356
26217013	23732519	19883397	8769777	21114891
12529448	19325137	12717443	19834490	17892308
22763125	22287577	23401740	16373578	11215515
24300896	9580552	29545238	14656735	12860264
10327068	12480817	18509061	18936167	4390543
15308628	10196157	15918795	12771128	20110595
20463056	15261145	18059339	15131009	17045981
18840614	15659776	20631299	19661463	19707742
14564009	28927665	17519230	15919658	6885824
11513746	9393862	29880492	28271280	1492101
9398617	15217908	17371830	18617643	18226574
20110358	18440775	10102632	10865940	3954771
15466206	18451337	23401860	12714333	11279066
28278510	12855698	18559514	20445537	14695536
28536097	22762016	23805312	11280761	16690356
28851877	17303569	11525641	19661918	12571671
12407018	18332134	22773844	19995915	18563633
18981713	22438576	18440854	19478092	11832420
18180305	21711246	20413783	20061392	6107306
11015619	29065929	28478454	15001553	15117956
17721515	10336480	28424170	18805968	17163662
17908694	20966350	25582201	21905169	15631778
15342917	16456544	21653897	9756915	19940234
11122379	17372230	18377662	29100366	16472406

3134526	17372190
12419480	24416395
6668142	23506894
15782407	17344318
16239338	11831458
12663279	23506888
9159205	22289350
11472746	24710731
19373259	22057392
1597321	
10468579	
12816923	
10377254	
17964036	
11012887	
31825581	
16763894	
28202503	
31867158	
25071589	
15128933	
20541252	
20736230	
18689389	
20418485	
7690968	
11111101	
22782502	
23923049	
21870057	
15205382	
24247221	
16399349	
24202443	
16186133	
17721433	
24726990	
16002434	
24975273	
22213316	
24854954	
19667142	
23999061	
21872797	
25163748	
21459326	
18800146	
20091054	
18483421	
16881513	
18957414	
