# Supplementary material for: CENTRA: knowledge-based gene contextuality graphs reveal functional master regulators by centrality and fractality
Source: NAR Genom Bioinform. 2025 Dec 19;7(4):lqaf196. doi: 10.1093/nargab/lqaf196 (PMC12714692; doi:10.1093/nargab/lqaf196)
Supplement: lqaf196_Supplemental_Files [file lqaf196_supplemental_files.zip › Supplementary Table S1 Network Properties.pdf]

Table S1: Topological properties of the 27 topic-specific gene co-occurrence networks. Each network was constructed by identifying shared genes across all pairwise combinations of gene sets within a given topic.

Topic	Assigned Documents	Nodes	Edges	Avg. Degree	Clustering Coef.	Graph Density	Dia-meter	Modu-larity	Avg. Betweenness	Avg. Closeness	Avg. Path Length
Hematopoietic Stem Cell Differentiation and Leukemogenesis	168	8,956	1,174,574	262.299	0.704	0.029	7	0.547	6488.517	0.002	2.455
Neurodegenerative Diseases and Mitochondrial Dysfunction	39	605	44,237	146.238	0.938	0.242	6	0.286	346.969	0.032	2.241
Cancer Types, Metastasis, and Therapeutic Targets	276	12,922	2,531,635	391.833	0.444	0.030	6	0.444	8426.451	< 0.001	2.305
Hedgehog Signaling, Ciliopathies, and Developmental Disorders	51	743	29,085	78.291	0.714	0.106	8	0.548	627.747	0.024	2.817
VEGF Signaling, Angiogenesis, and Endothelial Function	47	592	13,543	45.753	0.663	0.077	6	0.547	412.260	0.032	2.510
Kidney Development, Congenital Syndromes, and Genetic Disorders	40	1,301	21,004	32.289	0.622	0.025	8	0.732	1458.610	0.014	3.311
microRNA Regulation in Cancer and PI3K/AKT Signaling	99	3,725	118,177	63.451	0.429	0.017	6	0.499	3206.227	0.009	2.758
p53-Mediated Apoptosis and Cell Cycle Regulation	107	5,524	781,842	283.071	0.751	0.051	6	0.538	3561.920	0.001	2.292
Tissue Injury, Fibrosis, and Regeneration in the CNS and Heart	40	935	8,921	19.082	0.677	0.020	8	0.638	888.759	0.048	3.218
Innate and Adaptive Immune Signaling in Infection and Inflammation	159	5,355	538,210	201.012	0.612	0.038	6	0.517	3693.405	0.003	2.389
Hormone Signaling, Metal Homeostasis, and Reproductive Cancers	99	4,037	209,396	103.738	0.728	0.026	8	0.618	3324.777	0.008	2.677
DNA Damage Response and Repair Mechanisms	70	1,736	142,020	163.618	0.928	0.094	6	0.369	1159.657	0.014	2.383
MAPK Signaling Pathways and Kinase Regulation	37	577	31,713	109.924	0.948	0.191	8	0.157	321.000	0.038	2.296
Epigenetic Regulation and Chromatin Modifications	121	7,549	574,897	152.311	0.523	0.020	6	0.593	5859.306	0.004	2.567
Insulin Signaling, Metabolic Regulation, and Energy Homeostasis	93	2,600	292,295	224.842	0.830	0.087	6	0.500	1785.952	0.002	2.387
Rho GTPases and Cytoskeletal Dynamics	62	1,397	189,459	271.237	0.818	0.194	5	0.420	726.631	0.002	2.044
SARS-CoV-2 and RNA Virus Infection Mechanisms	69	3,313	216,500	130.697	0.727	0.039	7	0.599	2905.826	0.008	2.784
Notch Signaling in Development and Differentiation	49	405	6,538	32.286	0.774	0.080	9	0.637	134.244	0.091	2.668
Neural Receptors, Synaptic Plasticity, and Neurodevelopment	58	1,428	177,918	249.185	0.827	0.175	7	0.257	868.653	0.009	2.261
Wnt and TGF-β Signaling in Cell Division and Development	76	2,301	75,362	65.504	0.626	0.028	7	0.674	1963.628	0.018	2.791
Drug Metabolism, Cytochrome P450, and Pharmacokinetics	53	300	5,133	34.220	0.740	0.114	8	0.470	220.933	0.032	2.603
Nuclear Receptors and Xenobiotic Metabolism	17	336	16,067	95.637	0.875	0.285	4	0.399	132.202	0.002	1.789
Lipid Metabolism and Membrane Phospholipid Biosynthesis	47	312	5,819	37.301	0.717	0.120	9	0.563	337.337	0.021	3.362
Lipoprotein Metabolism, Cardiovascular Risk, and Genetic Dyslipidemias	19	48	248	10.333	0.857	0.220	2	0.535	1.458	0.122	1.220
Intracellular Membrane Trafficking and Ubiquitin-Mediated Transport	51	789	23,158	58.702	0.774	0.074	8	0.702	886.009	0.013	3.404
Oxidative Stress, Lipid Metabolism, and Inflammation	12	75	1,490	39.733	0.964	0.537	4	0.098	24.960	0.009	1.675
Enzyme-Catalyzed Metabolism and Bacterial Biosynthesis	93	596	10,821	36.312	0.953	0.061	9	0.653	344.240	0.055	3.652
